# Design, Synthesis, and Activity Assays of Cyclin-Dependent Kinase 1 Inhibitors With Flavone Scaffolds

**DOI:** 10.3389/fchem.2022.940427

**Published:** 2022-08-08

**Authors:** Lanlan Fu, Jiajia Mou, Yanru Deng, Xiaoliang Ren, Shuang Qiu

**Affiliations:** School of Chinese Materia Medica, Tianjin University of Traditional Chinese Medicine, Tianjin, China

**Keywords:** cyclin-dependent kinase 1, flavone, derivative, anti-tumor, inhibitor

## Abstract

Cyclin-dependent kinase 1 (CDK1) plays an indispensable role in the whole cell cycle. It has become a new target for cancer therapy. According to the binding mode of a pan-CDK inhibitor, flavopiridol with CDK1, and our previous work, a new series of flavone derivatives were discovered. Among them, compound **2a** showed the best CDK1 inhibitory and anti-proliferative potencies in the *in vitro* activity investigation. The IC_50_ of **2a** against CDK1 was 36.42 ± 1.12 μM vs. 11.49 μM ± 0.56 of flavopiridol. In the anti-proliferation activity assays, **2a** exhibited better activity toward RAW264.7 than MCF-7 cells. The results indicated that flavone derivatives, besides inhibiting the growth of tumor cells, can also antagonize inflammatory response. Molecular docking results showed that conformation of **2a** can form hydrogen bonds and various hydrophobic interactions with the key amino acid residues of CDK1. It can be used as a promising lead compound for CDK1 inhibitor development.

## 1 Introduction

Tumor is a common malignant disease, being one of the main diseases endangering human health. It seriously affects the life quality of human beings. It easily causes complications that lead to the continuous decline of life quality. According to the latest global cancer burden data in 2020 released by the International Agency for Research on Cancer of the World Health Organization, China has 4.57 million new cancer patients, accounting for 23.7% of the world’s total. There are 3 million cancer deaths in China, accounting for 30% of the total cancer deaths in the world mainly because of the large number of cancer patients in China. The number of cancer deaths ranks first worldwide (Sung et al., 2021). Gene mutations lead to endless cell proliferation, which induces tumor formation (Lapenna and Giordano, 2009). Traditional chemotherapy has several disadvantages, such as side effects, drug resistance, and so on (Bradner et al., 2017; Solaki and Ewald, 2018). It is of great urgency to find new targets and new anti-tumor drugs with high efficiency, selectivity, and low toxicity.

Since the discovery of the cell cycle regulation mechanism in the early 1970s, cell cycle checkpoints have become important targets for anti-tumor drug research (Sánchez-Martínez et al., 2015). There are three main cytokines involved in the regulation of the cell cycle: cyclins, cyclin-dependent kinases (CDKs), and cyclin-dependent kinase inhibitors (CDKIs) (Malumbre and Barbacid, 2005, 2009). CDKs belong to the serine/threonine kinase family. They are the key kinases in cell cycle regulation and the core of cell cycle regulation networks. They can form complexes with their cognate cyclins to exert their kinase activities. They can also be deactivated by their endogenous CDKIs (Denicourt and Dowdy, 2004; Deshpande et al., 2005). Their over-expression leads to uncontrolled cell proliferation, which is the main cause of tumors (Uziel et al., 2006). Therefore, inhibition of these abnormal CDKs is an important way for tumor therapy. CDK1 controls the entry from the G2 phase to the M phase in mammalian cells (Shapiro, 2006). It plays an indispensable role in the whole cell cycle. In the absence of inter-phase CDKs (CDK2, 3, 4, and 6), CDK1 can still drive all the events that are required in the cell cycle (Shapiro, 2006; Vassilev et al., 2006; Santamaría et al., 2007). Therefore, CDK1 has become a new target for selective CDK inhibitors.

**FIGURE 1:**
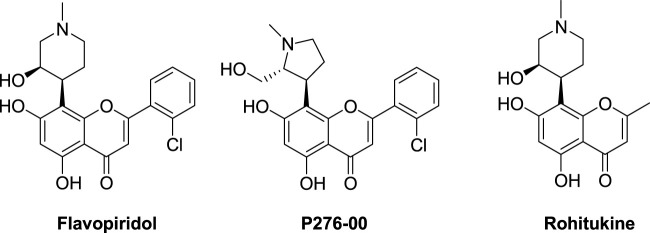
The structures of flavopiridol, P276-00, and rohitukine.

Flavonoids are polyphenolic compounds with planar aromatic heterocyclic scaffolds and are widely distributed in nature. They have various structural classifications and have a variety of pharmacological effects, such as antioxidant, anti-inflammatory, liver-protective, antibacterial, antiviral, and anti-tumor effects (Perez-Vizcaino and Fraga, 2018). In recent years, their anti-tumor effects have received much attention. One of their anti-tumor mechanisms is that they can directly or indirectly reduce the levels of CDKs, cyclin, and CDK-related proteins to inhibit the growth of tumor cells (Parajuli et al., 2009). It is worth mentioning that two flavone compounds, flavopiridol, a pan-CDK inhibitor, and p276-00 (Figure 1), derived from the natural product rohitukine, have very strong CDK inhibitory effects and are currently in clinical trials for tumor treatment (Cassaday et al., 2015; Blachly et al., 2016). This further confirms the feasibility of developing flavone derivatives as CDK inhibitors. In the co-crystal complex of CDK1/cyclin B-Cks2 with flavopiridol (PDB: 6GU2), the chromone core of flavopiridol is sandwiched between A31 and L135, forms two hydrogen bonds with the main-chain amide of L83 and carbonyl of E81 in the hinge region, and forms hydrophobic interactions with the gatekeeper residue F80 of CDK1. The piperidinol moiety forms a network of interactions with K33 and D146. Finally, the chlorophenyl group of flavopiridol forms hydrophobic interactions with V18 and I10 (Wood et al., 2019). The binding mode of flavopiridol with CDK1/cyclin B-Cks2 provides a basis for the design of CDK1 inhibitors with flavone scaffolds.

**FIGURE 2:**
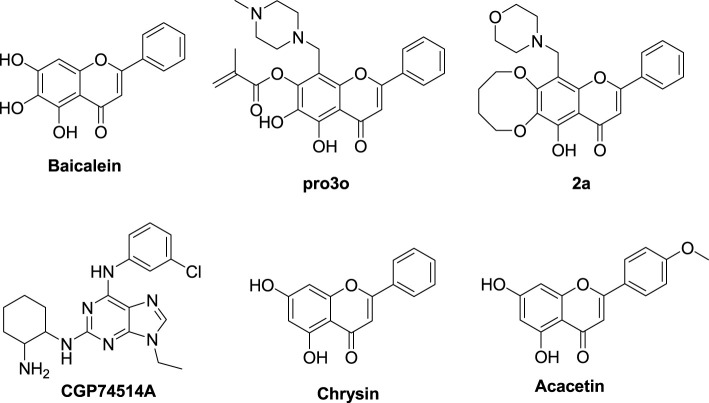
The structures of baicalein, baicalein’s derivatives, CGP74514A, chrysin, and acacetin.

**FIGURE 3:**
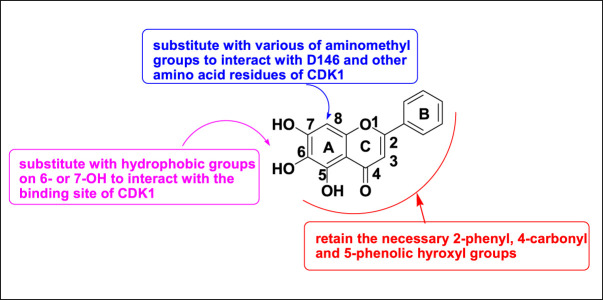
The strategy of baicalein modification.

Baicalein (Figure 2) is a natural flavonoid isolated from the root of *Scutellaria baicalensis* Georgi or from baicalin by hydrolysis (Shen et al., 2003). It can induce cell apoptosis and cell cycle arrest by down-regulating CDK1, CDK2, cyclin D2, and cyclin A, as well as upregulating CDKIs in G1 and G2 phases, and by downregulating the expression of CDK4/cyclin B and D (Eichhorn and Efferth, 2012). In our previous work, baicalein was modified as a CDK1 inhibitor according to the binding mode of flavopiridol with CDK1. The key chromone core and phenyl group of baicalein were retained, while hydrophobic groups were introduced to position six or seven of baicalein’s A ring, and various aminomethyl groups were introduced to position eight of baicalein’s A ring (Figure 3) (Mou et al., 2021a).

The baicalein derivatives remarkably inhibited the activity of CDK1/cyclin B kinase and the proliferation of MCF-7 tumor cells. Among them, compound pro3o (Figure 2) possessed the best activity against CDK1/cyclin B kinase (IC_50_ = 1.26 μM). Compound 2a (Figure 2) displayed a better inhibition rate (96.7%) than flavopiridol (90.0%) against MCF-7 tumor cell proliferation at a concentration of 50 μg/ml, and comparable to CGP74514A (Figure 2), a selective CDK1 inhibitor (96.7%) (Mou et al., 2021a). Like baicalein, chrysin, and acacetin (Figure 2) also belong to flavones. Chrysin can block rat C6 glioma cells in the G1 phase in a dose-dependent and time-dependent manner. It can also increase the expression of p21 and reduce the activities of CDK2 and 4. Its mechanism may be that chrysin can activate the P8 MAPK pathway, increase p21 expression, and inhibit the activity of proteases related to cell proliferation (Kasala et al., 2015). Acacetin shows a strong G1 and/or G2/M blockade effect, reduces the levels of CDK2, 4, and 6 in tumor cells in a dose-dependent and time-dependent manner, increases the level of p21, reduces the levels of cdc25c, CDK1, and cyclin B 1 protein, and makes cells stagnate in the G1 and G2/M phases (Singh et al., 2005). In general, baicalein, chrysin, and acacetin all have inhibitory effects on CDKs. Compared with other natural flavonoids, chrysin, and acacetin are similar to baicalein in structure. That is, there is no hydroxyl substitution in the B ring (this does not affect the structural modification of the hydroxyl group and position eight of the A ring), and there is no active substituent in the C ring. While there are still hydroxyl groups in the A ring that can be modified. Therefore, we can refer to the design, synthesis, activity, and selectivity evaluation strategies of baicalein derivatives, take CDK1 as the target, as well as carry out structure modification on chrysin and acacetin, along with some new baicalein derivatives to obtain more flavone derivatives. Through activity evaluation and structure-activity relationship (SAR) analysis, we can know the relationship between the compounds and their activities. This will provide a basis for guiding further research on CDK1 inhibitors with flavone scaffolds.

## 2 Materials and Methods

### 2.1 General

All solvents were analytical reagents, commercially available, and used without further purification. Thin-layer chromatography with silica gel pre-coated glass and fluorescent indicator was used to monitor the reactions. CDKs/cyclin kinases were provided by BPS Bioscience Inc. (California, United States). Kinase-Glo^®^ luminescent kinase assay platform was purchased from Promega Corporation (Madison, United States). MCF-7 cells and RAW264.7 cells were purchased from the Cell Bank of the Chinese Academy of Sciences, Shanghai, China. Dulbecco’s modified Eagle’Fdatas medium (DMEM) was purchased from Thermo Fisher Scientific (Grand Island, NY, United States). The Cell Counting Kit-8 (CCK-8) was purchased from Biodragon Immune technologies Co., Ltd. Beijing, China. ^1^H- and ^13^C-NMR spectra were recorded on a Bruker AV-III-600 instrument. High-resolution mass spectral (HRMS) data were determined on an Agilent 6540 UHD accurate mass Q-TOF MS instrument in low-resonance electrospray mode (ESI). A 96-well plate was measured on a microplate reader of Flex Station 3 (Molecular Devices, United States). Molecular docking was conducted on Discovery Studio, version 5.

### 2.2 Synthesis Procedures

#### 2.2.1 Synthesis of Compounds **1**

Compounds **1** were prepared as described in our published article (Mou et al., 2021b). And the spectral data of newly synthesized compounds **1** are consistent with the previous synthesized ones.

#### 2.2.2 Synthesis of Compounds **2**

Compounds **2** were prepared as described in our published article (Mou et al., 2021a).

##### 2.2.2.1 12-Hydroxy-7-(morpholinomethyl)-9-phenyl-2,3,4,5-tetrahydro-[1,4]dioxocino[2,3-g]chromen-11-one (2a)

Yellow crystal, yield 9.7%, mp 213.8–214.3°C. ^1^H-NMR (DMSO-*d*_*6*_ and 600 MHz) δ: 12.93 (s, 1H, and 5-OH), 8.10–8.11 (m, 2H, and H-2′, and 6′), 7.59–7.64 (m, 3H, H-3′,4′, and 5′), 7.02 (s, 1H, and H-3), 4.64 (t, *J* = 4.8 Hz, 2H, and H-10), 4.15 (t, *J* = 4.8 Hz, 2H, and H-13), 3.71 (s, 2H, and H-9), 3.53 (brs, 4H, H-15, and 16), 2.46–2.51 (m, 4H, H-14, and 17), 1.95–1.97 (m, 2H, and H-11), 1.73–1.75 (m, 2H, and H-12); ^13^C-NMR (DMSO-*d*_*6*_ and 150 MHz) δ: 183.1 (C-4), 163.9 (C-2), 158.0 (C-7), 153.6 (C-8a), 151.2 (C-5), 132.6 (C-6), 131.4 (C-4′), 131.1 (C-1′), 129.7 (C-3′ and 5′), 126.9 (C-2′ and 6′), 106.0 (C-4a), 105.0 (C-8), 73.5 (C-10), 72.2 (C-13), 66.8 (C-15 and 16), 53.5 (C-14 and 17), 50.2 (C-9), 28.7 (C-11), 24.7 (C-12); HRMS (ESI) m/z [M + H]^+^ Calcd for C_24_H_26_NO_6_ 424.1760 found 424.1814.

##### 2.2.2.2 12-Hydroxy-9-phenyl-7-(thiomorpholinomethyl)-2,3,4,5-tetrahydro-[1,4]dioxocino[2,3-g]chromen-11-one (2b)

Light yellow crystal, yield 21.6%, mp 202.3–203.6°C. ^1^H-NMR (DMSO-*d*_*6*_ and 600 MHz) δ: 12.93 (s, 1H, and 5-OH), 8.09–8.10 (m, 2H, H-2′, and 6′), 7.59–7.64 (m, 3H, H-3′,4′, and 5′), 7.02 (s, 1H, and H-3), 4.63 (t, *J* = 5.4 Hz, 2H, and H-10), 4.15 (t, *J* = 5.4Hz, 2H, and H-13), 3.71 (s, 2H, and H-9), 2.73 (brs, 4H, H-14, and 17), 2.51–2.57 (m, 4H, H-15, and 16), 1.94–1.96 (m, 2H, and H-11), 1.73–1.75 (m, 2H, and H-12); ^13^C-NMR (DMSO-*d*_*6*_ and 150 MHz) δ: 183.1 (C-4), 163.9 (C-2), 158.0 (C-7), 153.6 (C-8a), 151.2 (C-5), 132.6 (C-6), 131.4 (C-4′), 131.2 (C-1′), 129.7 (C-3′,5′), 126.8 (C-2′,6′), 107.2 (C-4a), 106.1 (C-3), 105.0 (C-8), 73.4 (C-10), 72.3 (C-13), 54.8 (C-14.17), 50.6 (C-9), 28.7 (C-11), 27.7 (C-15 and 16), and 24.7 (C-12); HRMS (ESI) m/z [M-H]^-^ Calcd for C_24_H_24_NO_5_S 438.1375 found 438.1458.

##### 2.2.2.3 7-((Dimethylamino)methyl)-12-hydroxy-9-phenyl-2,3,4,5-tetrahydro-[1,4]dioxocino[2,3-g]chromen-11-one (2c)

Yellow crystal, yield 0.66%, mp 208.5–210.6°C. ^1^H-NMR (DMSO-*d*_*6*_ and 600 MHz) δ: 12.93 (s, 1H, and 5-OH), 8.10–8.12 (m, 2H, H-2′, and 6′), 7.54–7.62 (m, 3H, H-3′,4′, and 5′), 7.06 (s, 1H, and H-3), 4.39 (s, 2H, and H-9), 4.19–4.20 (m, 2H, and H-10), 4.04–4.05 (m, 2H, and H-13), 2.09 (s, 6H, H-14, and 15), 1.50–1.58 (m, 4H, H-11, and 12); ^13^C-NMR (DMSO-*d*_*6*_ and 150 MHz) δ: 183.2 (C-4), 164.0 (C-2), 157.6 (C-7), 152.9 (C-8a), 150.2 (C-5), 132.7 (C-6), 131.4 (C-4′), 130.4 (C-1′), 129.7 (C-3′ and 5′), 126.9 (C-2′ and 6′), 108.8 (C-4a), 105.5 (C-3), 104.9 (C-8), 73.4 (C-10 and 13), 70.8 (C-9), and 28.8 (C-11 and 12).

#### 2.2.3 Synthesis of Compounds **3**

To a solution of **1a** or **1d** (4 mmol) in 30 ml DMF was added bromobenzyl or 1,4- dibromobutane (5.2 mmol), K_2_CO_3_ (12 mmol) and KI (12 mmol) successively. The mixture was stirred for 7 h under nitrogen atmosphere at 60–70°C. After that, the mixture was filtered under vacuum and a few drops of formic acid were added. The filtrate was then evaporated under vacuum and dispersed in cold water to form a suspension. The suspension was neutralized and filtered to obtain the crude product. The crude product was purified by silica gel column chromatography and recrystallization (Mou et al., 2021a).

##### 2.2.3.1 7-(Benzyloxy)-5,6-dihydroxy-8-(morpholinomethyl)-2-phenyl-4H-chromen-4-one (3a)

Yellow crystal, yield 2.24%, mp 218.3–219.8°C. ^1^H-NMR (DMSO-*d*_*6*_ and 600 MHz) δ: 12.91 (s, 1H, and 5-OH), 9.34 (s, 1H, and 6-OH), 8.11–8.14 (m, 2H, H-2′, and 6′), 7.61–7.64 (m, 5H, H-3′,4′, and 5′), 7.53–7.59 (m, 2H, H-3″, and 5″), 7.42–7.44 (m, 2H, H-2″, and 6″), 7.35–7.38 (m, 1H, and H-4″), 7.05 (s, 1H, and H-3), 5.25 (s, 2H, and H-10), 3.68 (s, 2H, and H-9), 3.50 (brs, 4H, H-12, and 13), 2.40 (brs, 4H, H-11, and 14); ^13^C-NMR (DMSO-*d*_*6*_ and 150 MHz) δ: 183.5 (C-4), 164.0 (C-2), 153.1 (C-7), 148.0 (C-8a), 147.9 (C-5), 137.9 (C-1″), 134.8 (C-4′), 132.6 (C-1′), 131.5 (C-6), 129.7 (C-3′ and 5′), 128.8 (C-3″ and 5″), 128.50 (C-4″), 128.4 (C-2″ and 6″), 126.9 (C-2′ and 6′), 107.2 (C-4a), 104.9 (C-3), 90.74 (C-8), 74.8 (C-10), 66.7 (C-12 and 13), 53.8 (C-11 and 14), and 50.8 (C-9).

##### 2.2.3.2 7-(4-Bromobutoxy)-8-((dipropylamino)methyl)-5,6-dihydroxy-2-phenyl-4H-chromen-4-one (3b)

Yellow crystal, yield 2.66%, mp 216.2–221.8°C. ^1^H-NMR (DMSO-*d*_*6*_ and 600 MHz) δ: 13.24 (s, 1H, and 5-OH), 8.68–8.70 (m, 1H, and 6-OH), 8.14 (brs, 2H, H-2′, and 6′), 7.62–7.68 (m, 3H, H-3′,4′, and 5′), 7.09 (s, 1H, and H-3), 4.83 (brs, 2H, and H-9), 4.55 (brs, 2H, and H-12), 4.20 (s, 2H, and H-13), 3.01–3.13 (m, 4H, H-14, and 17), 2.03–2.05 (m, 2H, and H-10), 1.71–1.78 (m, 6H, H-11,15, and 18), 0.82–0.83 (m, 6H, H-16, and 19); ^13^C-NMR (DMSO-*d*_*6*_ and 150 MHz) δ: 182.8 (C-4), 164.7 (C-2), 158.6 (C-7), 156.7 (C-8a), 152.0 (C-5), 132.8 (C-4′), 131.2 (C-1′), 129.6 (C-6), 129.5 (C-3′ and 5′), 127.5 (C-2′ and 6′), 106.0 (C-4a), 105.6 (C-3), 99.0 (C-8), 74.1 (C-9), 71.7 (C-13), 54.5 (C-14.17), 45.7 (C-12), 31.2 (C-10), 28.9 (C-11), 23.2 (C-15 and 18), 11.2 (C-16 and 19); HRMS (ESI) m/z [M + H]^+^ Calcd for C_26_H_33_BrNO_5_ 518.1505 found 518.1542.

#### 2.2.4 Synthesis of Compounds **4, 5**, and **6**

Compounds **4, 5,** and **6** were prepared by the method mentioned above for the preparation of compounds **1**.

##### 2.2.4.1 5,7-Dihydroxy-2-phenyl-6-(pyrrolidin-1-ylmethyl)-4H-chromen-4-one (4a)

Light yellow crystal, yield 39.7%, mp 195.2–196.3°C. ^1^H-NMR (DMSO-*d*_*6*_ and 600 MHz) δ: 8.24 (s, 1H, and 7-OH), 8.03–8.05 (m, 2H, H-2′, and 6′), 7.55–7.62 (m, 3H, H-3′,4′, and 5′), 6.89 (s, 1H, and H-3), 6.42 (s, 1H, and H-8), 4.08 (s, 2H, and H-9), 3.02–3.03 (m, 4H, H-10, and 13), 1.88–1.91 (m, 4H, H-11, and 12); ^13^C-NMR (DMSO-*d*_*6*_ and 150 MHz) δ: 181.7 (C-4), 169.2 (C-2), 164.4 (C-7), 163.0 (C-8a), 157.9 (C-5), 132.3 (C-4′), 131.4 (C-1′), 129.6 (C-3′ and 5′), 126.7 (C-2′ and 6′), 105.3 (C-4a), 103.8 (C-3), 102.2 (C-6), 94.9 (C-8), 53.4 (C-10 and13), 48.5 (C-9), 23.4 (C-11 and 12); HRMS (ESI) m/z [M + H]^+^ Calcd for C_20_H_20_NO_4_ 338.1392 found 338.1369.

##### 2.2.4.2 5,7-Dihydroxy-6-((4-methylpiperazin-1-yl)methyl)-2-phenyl-4H-chromen-4-one (4b)

Yellow crystal, yield 4.8%, mp 179.5–180.1°C. ^1^H-NMR (DMSO-*d*_*6*_ and 600 MHz) δ: 8.06–8.08 (m, 2H, H-2′, and 6′), 7.57–7.63 (m, 3H, H-3′,4′, and 5′), 6.98 (s, 1H, and H-3), 6.54 (s, 1H, and H-8), 3.76 (s, 2H, and H-9), 2.64 (brs, 8H, H-10,11, 12, and 13), 2.33 (s, 3H, and H-14); ^13^C-NMR (DMSO-*d*_*6*_ and 150 MHz) δ: 182.4 (C-4), 165.9 (C-2), 163.5 (C-7), 159.6 (C-8a), 157.0 (C-5), 132.5 (C-4′), 131.2 (C-1′), 129.6 (C-3′ and 5′), 126.9 (C-2′ and 6′), 105.5 (C-4a), 105.0 (C-3), 103.7 (C-6), 94.6 (C-8), 54.1 (C-11 and 13), 51.5 (C-10 and 12), 51.0 (C-9), and 45.0 (C-14); HRMS (ESI) m/z [M + H]^+^ Calcd for C_21_H_23_N_2_O_4_ 367.1658 found 367.1641.

##### 2.2.4.3 5,7-Dihydroxy-2-phenyl-6,8-bis(pyrrolidin-1-ylmethyl)-4H-chromen-4-one (5a)

Luminous yellow crystal, yield 49.9%, mp 194.9–196.6°C. ^1^H-NMR (DMSO-*d*_*6*_ and 600 MHz) δ: 8.06 (dd, *J*_1_ = 1.7 Hz, *J*_2_ = 5.2 Hz, 2H, H-2′, and 6′), 7.57–7.59 (m, 3H, H-3′,4′, and 5′), 6.88 (s, 1H, H-3), 3.91–3.92 (m, 4H, H-9, and 10), 2.77–2.78 (m, 4H, H-11, and 14), 2.61 (brs, 4H, H-15, and 18), 1.79–1.81 (m, 4H, H-12, and 13), 1.68 (brs, 4H, H-16, and 17); ^13^C-NMR (DMSO-*d*_*6*_ and 150 MHz) δ: 181.7 (C-4), 169.7 (C-2), 162.3 (C-7), 158.2 (C-8a), 155.2 (C-5), 132.1 (C-4′), 131.7 (C-1′), 129.6 (C-2′ and 6′), 126.6 (C-3′ and 5′), 105.0 (C-4a), 104.8 (C-6), 102.7 (C-8), 101.4 (C-3), 53.4 (C-15 and 18), 53.2 (C-11 and 14), 49.4 (C-10), 47.2 (C-9), and 23.6 (C-12,13, 16, and 17); HRMS (ESI) m/z [M + H]^+^ Calcd for C_25_H_29_N_2_O_4_ 421.2127 found 421.2201.

##### 2.2.4.5 6,8-Bis((2H-pyrrol-1(5H)-yl)methyl)-5,7-dihydroxy-2-phenyl-4H-chromen-4-one (5b)

Light yellow crystal, yield 6.0%, mp 172.7–174.3°C. ^1^H-NMR (DMSO-*d*_*6*_ and 600 MHz) δ: 8.07–8.08 (m, 2H, H-2′, and 6′), 7.57–7.62 (m, 3H, H-3′,4′, and 5′), 6.93 (s, 1H, and H-3), 5.85 (brs, 2H, H-12, and 13), 5.82 (brs, 2H, H-16, and 17), 4.14 (s, 2H, and H-10), 4.05 (s, 2H, and H-9), 3.65–3.67 (m, 8H, H-11,14, 15, and 18); ^13^C-NMR (DMSO-*d*_*6*_ and 150 MHz) δ: 182.0 (C-4), 168.8 (C-2), 162.6 (C-7), 158.6 (C-8a), 155.3 (C-5), 132.3 (C-4′), 131.5 (C-1′), 129.6 (C-3′ and 5′), 127.7 (C-2′ and 6′), 127.1 (C-12 and 13), 126.7 (C-16 and 17), 105.1 (C-4a), 104.9 (C-3), 102.1 (C-8), 101.9 (C-6), 59.3 (C-11 and 14), 59.2 (C-15 and 18), 49.5 (C-9), and 47.7 (C-10); HRMS (ESI) m/z [M + H]^+^ Calcd for C_25_H_25_N_2_O_4_ 417.1814 found 417.1860.

##### 2.2.4.6 5,7-Dihydroxy-6,8-bis(morpholinomethyl)-2-phenyl-4H-chromen-4-one (5c)

Yellow crystal, yield 28.0%, mp 199.2–199.6°C. ^1^H-NMR (DMSO-*d*_*6*_ and 600 MHz) δ: 13.35 (s, 1H, and 5-OH), 8.08–8.10 (m, 2H, H-2′, and 6′), 7.58–7.63 (m, 3H, H-3′,4′, and 5′), 6.99 (s, 1H, and H-3), 3.79 (s, 2H, and H-10), 3.76 (s, 2H, and H-9), 3.62–3.63 (m, 4H, H-12, and 13), 3.57–3.58 (m, 4H, H-16, and 17), 2.50–2.55 (m, 8H, H-11,14, 15, and 18); ^13^C-NMR (DMSO-*d*_*6*_ and 150 MHz) δ: 182.6 (C-4), 165.2 (C-2), 163.3 (C-7), 158.6 (C-8a), 155.1 (C-5), 132.5 (C-4′), 131.4 (C-1′), 129.7 (C-2′ and 6′), 126.8 (C-3′ and 5′), 105.4 (C-4a), 104.3 (C-6), 103.5 (C-8), 101.6 (C-3), 66.6 (C-16 and 17), 66.4 (C-12 and 13), 53.4 (C-15 and 18), 52.8 (C-11 and 14), 52.0 (C-10), 50.9 (C-9); HRMS (ESI) m/z [M + H]^+^ Calcd for C_25_H_29_N_2_O_6_ 453.2026 found 453.2131.

##### 2.2.4.7 5,7-Dihydroxy-2-phenyl-6,8-bis(thiomorpholinomethyl)-4H-chromen-4-one (5d)

Yellow crystal, yield 27.7%, mp 211.0–212.4°C. ^1^H-NMR (DMSO-*d*_*6*_ and 600 MHz) δ: 13.38 (s, 1H, and 5-OH), 8.08–8.10 (m, 2H, H-2′, and 6′), 7.58–7.64 (m, 3H, H-3′,4′, and 5′), 6.99 (s, 1H, and H-3), 3.81 (s, 2H, and H-9), 3.77 (s, 2H, and H-10), 2.78–2.82 (m, 8H, H-11,14, 15, and 18), 2.66–2.68 (m, 4H, H-12, and 13), 2.61–2.62 (m, 4H, H-16, and 17); ^13^C-NMR (DMSO-*d*_*6*_ and 150 MHz) δ: 182.6 (C-4), 165.5 (C-2), 163.3 (C-7), 158.6 (C-8a), 155.1 (C-5), 132.5 (C-4′), 131.4 (C-1′), 129.7 (C-3′ and 5′), 126.8 (C-2′ and 6′), 105.4 (C-4a), 104.4 (C-3), 103.5 (C-6), 101.8 (C-8), 54.6 (C-11 and 14), 54.2 (C-15 and 18), 52.3 (C-9), 51.3 (C-10), 27.6 (C-12 and 13), and 27.4 (C-16 and 17); HRMS (ESI) m/z [M + H]^+^ Calcd for C_25_H_29_N_2_O_4_S_2_ 485.1569 found 485.1558.

##### 2.2.4.8 5,7-Dihydroxy-6,8-bis((4-methylpiperazin-1-yl)methyl)-2-phenyl-4H-chromen-4-*one* (5e)

Light yellow crystal, yield 8.5%, mp 174.0.3–175.3°C. ^1^H-NMR (DMSO-*d*_*6*_ and 600 MHz) δ: 8.19 (s, 1H, and 7-OH), 8.09–8.10 (m, 2H, H-2′, and 6′), 7.59–7.64 (m, 3H, H-3′,4′, and 5′), 6.99 (s, 1H, and H-3), 3.86 (s, 2H, and H-9), 3.81 (s, 2H, and H-10), 2.29–2.32 (m, 8H, H-11,13, 16, and 17), 2.22–2.25 (m, 8H, H-12,14, 18, and 19), 2.20 (s, 6H, H-15, and 20); ^13^C-NMR (DMSO-*d*_*6*_ and 150 MHz) δ: 182.4 (C-4), 166.6 (C-2), 163.2 (C-7), 158.8 (C-8a), 155.3 (C-5), 132.5 (C-4′), 131.4 (C-1′), 129.7 (C-3′ and 5′), 126.8 (C-2′ and 6′), 105.4 (C-4a), 104.17 (C-3), 103.0 (C-6), 101.3 (C-8), 55.4 (C-12), 54.3 (C-14), 54.1 (C-18), 54.1 (C-19), 51.7 (C-11), 51.5 (C-13), 51.5 (C-16), 51.3 (C-7), 50.3 (C-9), 46.1 (C-10), 44.8 (C-15), and 43.2 (C-20); HRMS (ESI) m/z [M + H]^+^ Calcd for C_27_H_35_N_4_O_4_ 479.2658 found 479.2663.

##### 2.2.4.9 5,7-Dihydroxy-2-phenyl-8-(pyrrolidin-1-ylmethyl)-4H-chromen-4-one (6a)

Yellow crystal, yield 5.2%, mp 192.9–193.0°C. ^1^H-NMR (DMSO-*d*_*6*_ and 600 MHz) δ: 12.91 (s, 1H, and 5-OH), 8.23 (s, 1H, and 7-OH), 8.08–8.10 (m, 2H, and H-2′,6′), 7.58–7.63 (m, 3H, H-3′,4′, and 5′), 6.94 (s, 1H, and H-3), 6.15 (s, 1H, and H-6), 4.24 (s, 2H, and H-9), 2.96–2.98 (m, 4H, H-10, and 13), 1.83–1.88 (m, 4H, H-11, and 12); ^13^C-NMR (DMSO-*d*_*6*_ and 150 MHz) δ: 181.9 (C-4), 168.4 (C-2), 164.4 (C-7), 162.8 (C-8a), 155.7 (C-5), 132.3 (C-4′), 131.5 (C-1′), 129.7 (C-3′ and 5′), 126.9 (C-2′ and 6′), 105.5 (C-4a), 102.9 (C-3), 100.0 (C-8), 99.8 (C-6), 53.4 (C-10.13), 48.7 (C-9), and 23.4 (C-11 and 12); HRMS (ESI) m/z [M + H]^+^ Calcd for C_20_H_20_NO_4_ 338.1392 found 338.1373.

##### 2.2.4.10 5,7-Dihydroxy-8-(morpholinomethyl)-2-phenyl-4H-chromen-4-one (6b)

Yellow crystal, yield 25.6%, mp 217.6–219.5°C. ^1^H-NMR (DMSO-*d*_*6*_ and 600 MHz) δ: 12.89 (s, 1H, and 5-OH), 8.09–8.10 (m, 2H, H-2′, and 6′), 7.59–7.64 (m, 3H, H-3′,4′, and 5′), 6.99 (s, 1H, and H-3), 6.26 (s, 1H, and H-6), 3.84 (s, 2H, and H-9), 3.59–3.60 (m, 4H, H-11, and 12), 2.54–2.55 (m, 4H, H-10, and 13); ^13^C-NMR (DMSO-*d*_*6*_, and 150 MHz) δ: 182.6 (C-4), 164.7 (C-2), 163.4 (C-7), 160.9 (C-8a), 155.8 (C-5), 132.5 (C-4′), 131.4 (C-1′), 129.7 (C-3′ and 5′), 126.9 (C-2′ and 6′), 105.5 (C-4a), 104.3 (C-3), 101.5 (C-8), 99.2 (C-6), 66.6 (C-11.12), 53.2 (C-10 and 13), and 51.4 (C-9); HRMS (ESI) m/z [M-H]^-^ Calcd for C_20_H_18_NO_5_ 352.1185 found 352.1205.

##### 2.2.4.11 5,7-Dihydroxy-2-phenyl-8-(thiomorpholinomethyl)-4H-chromen-4-one (6c)

Luminous yellow crystal, yield 80.8%, mp 218.3–219.5°C. ^1^H-NMR (DMSO-*d*_*6*_ and 600 MHz) δ: 12.89 (s, 1H, and 5-OH), 8.08–8.10 (m, 2H, H-2′, and 6′), 7.59–7.61 (m, 3H, H-3′,4′, and 5′), 6.99 (s, 1H, and H-3), 6.25 (s, 1H, and H-6), 3.86 (s, 2H, and H-9), 2.82 (t, *J* = 4.8 Hz, 4H, H-10, and 13), 2.64–2.65 (m, 4H, H-11, and 12); ^13^C-NMR (DMSO-*d*_*6*_ and 150 MHz) δ: 182.6 (C-4), 164.8 (C-2), 163.4 (C-7), 160.8 (C-8a), 155.8 (C-5), 132.5 (C-4′), 131.4 (C-1′), 129.7 (C-3′ and 5′), 126.9 (C-2′ and 6′), 105.5 (C-4a), 104.3 (C-3), 101.5 (C-8), 99.3 (C-6), 54.6 (C-10, and 13), 52.0 (C-9), and 27.6 (C-11.12); HRMS (ESI) m/z [M + H]^+^ Calcd for C_20_H_20_NO_4_S 370.1113 found 370.1103.

##### 2.2.4.12 8-((Dimethylamino)methyl)-5,7-dihydroxy-2-phenyl-4H-chromen-4-one (6d)

Yellow crystal, yield 1.3%, mp 241.9–242.3°C. ^1^H-NMR (DMSO-*d*_*6*_ and 600 MHz) δ: 13.85 (s, 1H, and 5-OH), 7.80–7.82 (m, 2H, H-2′, and 6′), 7.61–7.65 (m, 3H, H-3′,4′, and 5′), 6.91 (s, 1H, and H-3), 6.14 (s, 1H, and H-6), 4.16 (s, 2H, and H-9), 2.75 (s, 6H, H-10, and 11); ^13^C-NMR (DMSO-*d*_*6*_ and 150 MHz) δ: 182.4 (C-4), 166.9 (C-2), 163.1 (C-7), 159.8 (C-8a), 155.3 (C-5), 132.0 (C-4′), 131.4 (C-1′), 129.2 (C-3′ and 5′), 126.3 (C-2′ and 6′), 106.5 (C-4a), 105.3 (C-3), 103.8 (C-8), 101.0 (C-6), 51.8 (C-9), and 42.8 (C-10 and 11); HRMS (ESI) m/z [M + H]^+^ Calcd for C_18_H_18_NO_4_ 312.1236 found 312.1230.

#### 2.2.5 Synthesis of Compounds **7**

Compounds **7** were prepared by the method mentioned above for the preparation of compounds **3**.

##### 2.2.5.1 7-(Benzyloxy)-5-hydroxy-2-phenyl-4H-chromen-4-one (7a)

Yellow crystal, yield 46.0%, mp 172.7–174.5°C. ^1^H-NMR (DMSO-*d*_*6*_ and 600 MHz) δ: 12.82 (s, 1H, and 5-OH), 8.08–8.10 (m, 2H, H-2′, and 6′), 7.62–7.64 (m, 3H, H-3′,4′, and 5′), 7.58–7.59 (m, 2H, H-2″, and 6″), 7.38–7.43 (m, 2H, H-3″, and 5″), 7.36–7.37 (m, 1H, and H-4″), 7.03 (s, 1H, and H-3), 6.90 (s, 1H, and H-8), 6.48 (s, 1H, and H-6), 5.25 (s, 2H, and H-9); ^13^C-NMR (DMSO-*d*_*6*_ and 150 MHz) δ: 182.6 (C-4), 164.8 (C-2), 164.0 (C-7), 161.7 (C-8a), 157.8 (C-5), 136.6 (C-1″), 132.6 (C-4′), 129.6 (C-3′ and 5′), 129.0 (C-3″ and 5″), 128.6 (C-1′), 128.4 (C-2″ and 6″), 128.0 (C-4″), 126.9 (C-2′ and 6′), 105.9 (C-4a), 105.5 (C-3), 99.2 (C-8), 94.1 (C-6), and 70.5 (C-9); MS (ESI, positive) *m/z*: 345.55 [M + H]^+^.

##### 2.2.5.2 5-Hydroxy-7-methoxy-2-phenyl-4H-chromen-4-one (7b)

Yellow-white crystal, yield 49.4%, mp 168.6–170.1°C. ^1^H-NMR (DMSO-*d*_*6*_ and 600 MHz) δ: 12.81 (s, 1H, and 5-OH), 8.09–8.10 (m, 2H, H-2′, and 6′), 7.57–7.64 (m, 3H, H-3′,4′, and 5′), 7.03 (s, 1H, and H-3), 6.80 (d, *J* = 2.2 Hz, 1H, and H-8), 6.39 (d, *J* = 2.2 Hz, 1H, and H-6), 3.88 (s, 3H, and OCH_3_); ^13^C-NMR (DMSO-*d*_*6*_ and 150 MHz) δ: 182.6 (C-4), 165.8 (C-2), 163.9 (C-7), 161.7 (C-8a), 157.8 (C-5), 132.6 (C-4′), 131.1 (C-1′), 129.6 (C-3′ and 5′), 126.9 (C-2′ and 6′), 105.8 (C-4a), 105.4 (C-3), 98.6 (C-8), 93.3 (C-6), and 56.6 (OCH_3_); MS (ESI, positive) *m/z*: 269.11 [M + H]^+^.

##### 2.2.5.3 7-(2-Bromoethoxy)-5-hydroxy-2-phenyl-4H-chromen-4-one (7c)

Yellow crystal, yield 8.6%, mp 169.3–170.1°C. ^1^H-NMR (DMSO-*d*_*6*_ and 600 MHz) δ: 12.81 (s, 1H, and 5-OH), 8.09–8.11 (m, 2H, H-2′, and 6′), 7.60–7.65 (m, 3H, H-3′,4′, and 5′), 7.04 (s, 1H, and H-3), 6.86 (s, 1H, and H-8), 6.42 (s, 1H, and H-6), 4.46–4.48 (t, *J* = 5.4 Hz, 2H, and H-9), 3.85 (brs, 2H, and H-10); ^13^C-NMR (DMSO-*d*_*6*_ and 150 MHz) δ: 182.6 (C-4), 164.4 (C-2), 164.0 (C-7), 161.7 (C-8a), 157.8 (C-5), 132.7 (C-4′), 131.0 (C-1′), 129.6 (C-3′ and 5′), 127.0 (C-2′ and 6′), 105.7 (C-4a), 105.4 (C-3), 99.0 (C-6), 93.9 (C-8), 69.0 (C-9), and 31.3 (C-10); HRMS (ESI) m/z [M + K]^+^ Calcd for C_17_H_13_BrKO_4_ 398.9634 found 398.1955.

##### 2.2.5.4 7-(4-Bromobutoxy)-5-hydroxy-2-phenyl-4H-chromen-4-one (7d)

Luminous yellow crystal, yield 5.4%, mp 162.6–163.6°C. ^1^H-NMR (DMSO-*d*_*6*_ and 600 MHz) δ: 12.79 (s, 1H, and 5-OH), 8.08 (d, *J* = 4.0 Hz, 2H, H-2′, and 6′), 7.59–7.64 (m, 3H, H-3′,4′, and 5′), 7.01 (s, 1H, and H-3), 6.78 (d, *J* = 2.2 Hz, 1H, and H-8), 6.37 (d, *J* = 2.2 Hz, 1H, and H-6), 4.10–4.13 (m, 2H, and H-9), 3.63 (t, *J* = 6.7 Hz, 2H, and H-12), 1.97–1.99 (m, 2H, and H-10), 1.86–1.87 (m, 2H, and H-11); ^13^C-NMR (DMSO-*d*_*6*_ and 150 MHz) δ: 182.5 (C-4), 165.1 (C-2), 163.9 (C-7), 161.7 (C-8a), 157.8 (C-5), 132.6 (C-4′), 131.1 (C-1′), 129.6 (C-3′ and 5′), 126.9 (C-2′ and 6′), 105.8 (C-4a), 105.4 (C-3), 98.9 (C-6), 93.7 (C-8), 68.1 (C-9), 35.2 (C-12), 29.3 (C-10), and 27.6 (C-11); HRMS (ESI) m/z [M-H]^-^ Calcd for C_19_H_16_BrO_4_ 387.0232 found 387.1165.

#### 2.2.6 Synthesis of Compounds **8**

Compounds **8** were prepared by the method mentioned above for the preparation of compounds **1**.

##### 2.2.6.1 5-Hydroxy-7-methoxy-6-(morpholinomethyl)-2-phenyl-4H-chromen-4-one (8a)

Light yellow crystal, yield 22.3%, mp 187.0–188.6°C. ^1^H-NMR (DMSO-*d*_*6*_ and 600 MHz) δ: 13.14 (s, 1H, and 5-OH), 8.11–8.12 (m, 2H, H-2′, and 6′), 7.58–7.65 (m, 3H, H-3′,4′, and 5′), 7.06 (s, 1H, and H-8), 6.92 (s, 1H, and H-3), 3.90–3.92 (m, 3H, and OCH_3_), 3.50–3.53 (m, 6H, H-9,11, and 13), 2.40–2.45 (m, 4H, H-10, and 12); ^13^C-NMR (DMSO-*d*_*6*_ 150 MHz) δ: 182.6 (C-4), 164.7 (C-2), 163.8 (C-7), 159.8 (C-8a), 157.2 (C-5), 132.6 (C-4′), 131.1 (C-1′), 129.6 (C-3′ and 5′), 126.9 (C-2′ and 6′), 108.4 (C-4a), 105.9 (C-3), 105.0 (C-6), 91.2 (C-8), 66.7 (C-11 and 13), 56.9 (OCH_3_), 53.5 (C-10.12), and 48.9 (C-9); HRMS (ESI) m/z [M + H]^+^ Calcd for C_21_H_22_NO_5_ 368.1498 found 368.1478.

##### 2.2.6.2 5-Hydroxy-7-methoxy-2-phenyl-6-(thiomorpholinomethyl)-4H-chromen-4-one (8b)

Yellow-white crystal, yield 42.5%, mp 210.3–212.0°C. ^1^H-NMR (DMSO-*d*_*6*_ and 600 MHz) δ: 13.17 (s, 1H, and 5-OH), 8.10–8.13 (m, 2H, H-2′, and 6′), 7.58–7.65 (m, 3H, H-3′,4′, and 5′), 7.06 (s, 1H, and H-8), 6.92 (s, 1H, and H-3), 3.92 (s, 3H, and OCH_3_), 3.53 (s, 2H, and H-9), 2.68–2.72 (m, 4H, H-10, and 12), 2.54–2.57 (m, 4H, H-11, and 13); ^13^C-NMR (DMSO-*d*_*6*_ and 150 MHz) δ: 182.6 (C-4), 164.8 (C-2), 163.8 (C-7), 159.8 (C-8a), 157.2 (C-5), 132.6 (C-4′), 131.1 (C-1′), 129.6 (C-3′ and 5′), 126.9 (C-2′ and 6′), 108.6 (C-4a), 105.9 (C-3), 105.0 (C-6), 91.2 (C-8), 57.0 (C-10 and 12), 54.7 (OCH_3_), 49.4 (C-9), and 27.6 (C-11 and 13); HRMS (ESI) m/z [M + H]^+^ Calcd for C_21_H_22_NO_4_S 384.1270 found 384.1241.

#### 2.2.7 Synthesis of Compounds **9, 10**, and **11**

Compounds **9, 10,** and **11** were prepared by the method mentioned above for the preparation of compounds **1**.

##### 2.2.7.1 5,7-Dihydroxy-2-(4-methoxyphenyl)-6-((4-methylpiperazin-1-yl)methyl)-4H-chromen-4-one (9a)

Yellow crystal, yield 9.9%, mp 172.3–173.4°C. ^1^H-NMR (DMSO-*d*_*6*_ and 600 MHz) δ: 13.48 (s, 1H, and 5-OH), 8.03 (dd, *J*_1_ = 6.8 Hz, *J*_2_ = 2.2 Hz, 2H, H-2′, and 6′), 7.10–7.12 (m, 2H, H-3′, and 5′), 6.87 (s, 1H, and H-3), 6.52 (s, 1H, and H-8), 3.86 (s, 3H, and OCH_3_), 3.75 (s, 2H, and H-9), 2.63 (brs, 8H, H-10,11, 12, and 13), 2.32 (s, 3H, and H-14); ^13^C-NMR (DMSO-*d*_*6*_ and 150 MHz) δ: 182.3 (C-4), 165.6 (C-2), 163.7 (C-7), 162.8 (C-8a), 159.6 (C-4′), 156.9 (C-5), 128.8 (C-2′ and 6′), 123.3 (C-1′), 115.1 (C-3′ and 5′), 104.8 (C-4a), 103.9 (C-3), 103.5 (C-6), 94.4 (C-8), 56.0 (OCH_3_), 54.2 (C-12 and 13), 51.6 (C-10 and 11), 51.0 (C-9), and 45.0 (C-14); HRMS (ESI) m/z [M + H]^+^ Calcd for C_22_H_25_N_2_O_5_ 397.1763 found 397.1754.

##### 2.2.7.2 6-((Dipropylamino)methyl)-5,7-dihydroxy-2-(4-methoxyphenyl)-4H-chromen-4-one (9b)

Yellow crystal, yield 19.5%, mp 180.6–181.3°C. ^1^H-NMR (DMSO-*d*_*6*_ and 600 MHz) δ: 8.18 (s, 1H, and 7-OH), 8.01 (dd, *J*_1_ = 6.8 Hz, *J*_2_ = 2.2 Hz, 2H, H-2′, and 6′), 7.09–7.11 (m, 2H, H-3′, and 5′), 6.82 (s, 1H, and H-3), 6.38 (s, 1H, and H-8), 3.92 (s, 2H, and H-9), 3.86 (s, 3H, and OCH_3_), 2.60–2.63 (m, 4H, H-10, and 13), 1.55–1.60 (m, 4H, H-11, and 14), 0.87 (t, *J* = 7.3 Hz, 6H, H-12, and 15); ^13^C-NMR (DMSO-*d*_*6*_ and 150 MHz) δ: 182.0 (C-4), 168.2 (C-2), 163.4 (C-7), 162.7 (C-8a), 159.0 (C-4′), 157.2 (C-5), 128.7 (C-2′ and 6′), 123.4 (C-1′), 115.0 (C-3′ and 5′), 104.0 (C-4a), 103.8 (C-3), 102.7 (C-6), 94.8 (C-8), 56.0 (C-10 and 13), 55.0 (OCH_3_), 49.6 (C-9), 18.8 (C-11 and 14), and 11.9 (C-12 and 15); HRMS (ESI) m/z [M + H]^+^ Calcd for C_23_H_28_NO_5_ 398.1967 found 398.1949.

##### 2.2.7.3 5,7-Dihydroxy-2-(4-methoxyphenyl)-6,8-bis(pyrrolidin-1-ylmethyl)-4H-chromen-4-one (10a)

Luminous yellow crystal, yield 9.4%, mp 207.1–207.3°C. ^1^H-NMR (DMSO-*d*_*6*_ and 600 MHz) δ: 13.47 (s, 1H, and 5-OH), 8.03 (d, *J* = 8.8 Hz, 2H, H-2′, and 6′), 7.12 (d, *J* = 8.8 Hz, 2H, H-3′, and 5′), 6.87 (s, 1H, and H-3), 3.86 (s, 3H, and OCH_3_), 3.79 (s, 2H, and H-10), 3.76 (s, 2H, and H-9), 2.78–2.80 (m, 8H, H-11,14, 15, and 18), 2.66–2.68 (m, 4H, H-12, and 13), 2.60–2.62 (m, 4H, H-16, and 17); ^13^C-NMR (DMSO-*d*_*6*_ and 150 MHz) δ: 182.5 (C-4), 165.1 (C-2), 163.4 (C-7), 162.8 (C-8a), 158.6 (C-4′), 154.9 (C-5), 128.7 (C-2′ and 6′), 123.5 (C-1′), 115.1 (C-3′ and 5′), 104.2 (C-4a), 103.7 (C-6), 103.3 (C-8), 101.7 (C-3), 56.0 (OCH_3_), 54.7 (C-15 and 18), 54.2 (C-11 and 14), 52.3 (C-10), 51.2 (C-9), and 27.4 (C-12,13, 16, and 17); HRMS (ESI) m/z [M + H]^+^ Calcd for C_26_H_31_N_2_O_5_ 451.2233 found 451.1507.

##### 2.2.7.4 5,7-Dihydroxy-2-(4-methoxyphenyl)-6,8-bis(thiomorpholinomethyl)-4H-chromen-4-one (10b)

Yellow crystal, yield 10.9%, mp 214.2–215.5°C. ^1^H-NMR (DMSO-*d*_*6*_ and 600 MHz) δ: 13.48 (s, 1H, and 5-OH), 8.03–8.05 (m, 2H, H-2′, and 6′), 7.11–7.14 (m, 2H, H-3′, and 5′), 6.88 (s, 1H, and H-3), 3.87 (s, 3H, and OCH_3_), 3.79 (s, 2H, and H-9), 3.76 (s, 2H, and H-10), 2.79–2.81 (m, 8H, H-11,12, 15, and 16), 2.66–2.68 (m, 4H, H-13, and 14), 2.60–2.62 (m, 4H, H-17, and 18); ^13^C-NMR (DMSO-*d*_*6*_ and 150 MHz) δ: 182.5 (C-4), 165.1 (C-2), 163.5 (C-7), 162.8 (C-8a), 158.6 (C-4′), 155.0 (C-5), 128.7 (C-2′ and 6′), 123.6 (C-1′), 115.2 (C-3′ and 5′), 104.3 (C-4a), 103.8 (C-3), 103.3 (C-6), 101.7 (C-8), 56.0 (C-11 and 12), 54.7 (C-15 and 16), 54.2 (OCH_3_), 52.3 (C-9), 51.2 (C-10), 27.6 (C-13 and 14), and 27.4 (C-17 and 18); HRMS (ESI) m/z [M-H]^-^ Calcd for C_26_H_29_N_2_O_5_S_2_ 513.1518 found 513.1540.

##### 2.2.7.5 5,7-Dihydroxy-2-(4-methoxyphenyl)-6,8-bis((4-methylpiperazin-1-yl)methyl)-4H-chromen-4-one (10c)

Yellow crystal, yield 22.4%, mp 161.8–161.9°C. ^1^H-NMR (DMSO-*d*_*6*_ and 600 MHz) δ: 8.21 (s, 1H, and 7-OH), 8.04 (dd, *J*_1_ = 6.7 Hz, *J*_2_ = 2.2 Hz, 2H, H-2′, and 6′), 7.12–7.14 (d, *J* = 8.9Hz, 2H, H-3′, and 5′), 6.87 (s, 1H, and H-3), 3.86 (s, 3H, and OCH_3_), 3.80 (m, 4H, H-9, and 10), 2.62 (brs, 8H, H-11,13, 16, and 18), 2.49 (brs, 8H, H-12,14, 17, and 19), 2.20–2.26 (m, 6H, H-15, and 20); ^13^C-NMR (DMSO-*d*_*6*_ and 150 MHz) δ: 182.4 (C-4), 166.0 (C-2), 163.3 (C-7), 162.8 (C-8a), 158.6 (C-4′), 155.1 (C-5), 128.7 (C-2′ and 6′), 123.6 (C-1′), 115.1 (C-3′ and 5′), 104.0 (C-4a), 103.7 (C-3), 102.9 (C-6), 101.4 (C-8), 56.0 (C-12.14), 55.5 (OCH_3_), 54.3 (C-17 and 19), 51.9 (C-11 and 13), 51.8 (C-16), 51.7 (C-18), 51.4 (C-10), 50.3 (C-9), 46.1 (C-15), and 43.2 (C-20); HRMS (ESI) m/z [M + H]^+^ Calcd for C_28_H_37_N_4_O_5_ 509.2764 found 509.2745.

##### 2.2.7.6 6,8-Bis((dimethylamino)methyl)-5,7-dihydroxy-2-(4-methoxyphenyl)-4H-chromen-4-one (10d)

Yellow crystal, yield 21.9%, mp 191.6–193.7°C. ^1^H-NMR (DMSO-*d*_*6*_ and 600 MHz) δ: 8.01 (d, *J* = 8.9 Hz, 2H, H-2′, and 6′), 7.12 (d, *J* = 8.9 Hz, 2H, H-3′, and 5′), 6.79 (s, 1H, and H-3), 3.86 (s, 3H, and OCH_3_), 3.76 (s, 2H, and H-9), 3.68 (s, 2H, and H-10), 2.37 (s, 6H, H-11, and 12), 2.26 (s, 6H, H-13, and 14); ^13^C-NMR (DMSO-*d*_*6*_ and 150 MHz) δ: 181.9 (C-4), 168.6 (C-2), 162.7 (C-7), 162.6 (C-8a), 158.3 (C-4′), 155.3 (C-5), 128.5 (C-2′ and 6′), 123.8 (C-1′), 115.0 (C-3′ and 5′), 104.1 (C-4a), 103.4 (C-6), 102.5 (C-8), 101.8 (C-3), 56.0 (OCH_3_), 53.2 (C-10), 51.4 (C-9), 45.1 (C-13 and 14), and 43.8 (C-11 and 12); HRMS (ESI) m/z [M + H]^+^ Calcd for C_22_H_27_N_2_O_5_ 399.1920 found 399.1679.

##### 2.2.7.7 5,7-Bihydroxy-2-(4-methoxyphenyl)-8-(thiomorpholinomethyl)-4H-chromen-4-one (11a)

Yellow crystal, yield 47.3%, mp 211.0–213.4°C. ^1^H-NMR (DMSO-*d*_*6*_ and 600 MHz) δ: 12.98 (s, 1H, and 5-OH), 8.01–8.05 (m, 2H, H-2′, and 6′), 7.10–7.14 (m, 2H, H-3′, and 5′), 6.86–6.88 (m, 1H, and H-3), 6.22 (s, 1H, and H-6), 3.86 (s, 3H, and OCH_3_), 3.73 (s, 2H, and H-9), 2.79–2.89 (m, 4H, H-10, and 11), 2.63–2.67 (m, 4H, H-12, and 13); ^13^C-NMR (DMSO-*d*_*6*_ and 150 MHz) δ: 182.5 (C-4), 163.7 (C-2), 163.5 (C-7), 162.8 (C-8a), 160.8 (C-5), 159.5 (C-4′), 128.8 (C-2′ and 6′), 123.5 (C-1′), 115.1 (C-3′ and 5′), 104.9 (C-4a), 103.8 (C-3), 101.4 (C-8), 99.1 (C-6), 56.0 (C-10 and 11), 54.6 (OCH_3_), 52.0 (C-9), and 27.6 (C-12 and 13); HRMS (ESI) m/z [M + H]^+^ Calcd for C_21_H_22_NO_5_S 400.1219 found 400.1196.

#### 2.2.8 Synthesis of Compounds **12**

Compounds **12** were prepared by the method mentioned above for the preparation of compounds **3**.

##### 2.2.8.1 7-(Benzyloxy)-5-hydroxy-2-(4-methoxyphenyl)-4H-chromen-4-one (12a)

Yellow crystal, yield 3.7%, mp 181.6–182.7°C. ^1^H-NMR (DMSO-*d*_*6*_ and 600 MHz) δ: 12.92 (s, 1H, and 5-OH), 7.97–8.04 (m, 2H, H-2′, and 6′), 7.35–7.62 (m, 5H, H-2″,3″,4″,5″, and 6″), 7.06–7.11 (m, 2H, H-3′, and 5′), 6.91 (s, 1H, and H-3), 6.86–6.88 (m, 1H, and H-8), 6.45 (s, 1H, and H-6), 5.24 (m, 2H, and H-9), 3.85 (s, 3H, and OCH_3_); ^13^C-NMR (DMSO-*d*_*6*_ and 150 MHz) δ: 182.4 (C-4), 164.1 (C-2), 163.0 (C-7), 161.7 (C-8a), 159.5 (C-4′), 157.6 (C-5), 136.6 (C-3″ and 5″), 128.8 (C-1″), 128.2 (C-2′ and 6′), 128.0 (C-4″), 127.3 (C-2″ and 6″), 123.1 (C-1′), 114.9 (C-3′ and 5′), 105.3 (C-4a), 104.2 (C-3), 98.6 (C-6), 95.0 (C-8), 70.5 (C-9), and 52.5 (OCH_3_).

##### 2.2.8.2 5-Hydroxy-7-methoxy-2-(4-methoxyphenyl)-4H-chromen-4-one (12b)

White crystal, yield 56.1%, mp 181.0–183.5°C. ^1^H-NMR (DMSO-*d*_*6*_ and 600 MHz) δ: 12.91 (s, 1H, and 5-OH), 8.03–8.05 (m, 2H, H-2′, and 6′), 7.10–7.12 (m, 2H, H-3′, and 5′), 6.91 (s, 1H, and H-3), 6.76 (d, *J* = 2.2Hz, 1H, and H-8), 6.36 (d, *J* = 2.2Hz, 1H, and H-6), 3.86–3.87 (m, 6H, 7-OCH_3_, and 4′-OCH_3_); ^13^C-NMR (DMSO-*d*_*6*_ and 150 MHz) δ: 182.4 (C-4), 165.6 (C-2), 164.0 (C-7), 162.9 (C-8a), 161.7 (C-4′), 157.7 (C-5), 128.8 (C-2′ and 6′), 123.2 (C-1′), 115.0 (C-3′ and 5′), 105.2 (C-4a), 104.1 (C-3), 98.5 (C-6), 93.2 (C-8), 56.5 (7-OCH_3_), and 56.0 (4′-OCH_3_); MS (ESI, positive) *m/z*: 299.15 [M + H]^+^.

##### 2.2.8.3 7-(2-Bromoethoxy)-5-hydroxy-2-(4-methoxyphenyl)-4H-chromen-4-one (12c)

Yellow crystal, yield 10.1%, mp 175.2–175.7°C. ^1^H-NMR (DMSO-*d*_*6*_ and 600 MHz) δ: 12.92 (s, 1H, and 5-OH), 8.04–8.07 (m, 2H, H-2′, and 6′), 7.10–7.13 (m, 2H, H-3′, and 5′), 6.93 (s, 1H, and H-3), 6.83 (s, 1H, and H-8), 6.40 (s, 1H, and H-6), 4.45–4.47 (m, 2H, and H-9), 3.87 (s, 3H, and OCH_3_), 3.84–3.86 (t, *J* = 5.6Hz, 2H, and H-10); ^13^C-NMR (DMSO-*d*_*6*_, and 150 MHz) δ: 182.4 (C-4), 164.2 (C-2), 164.2 (C-7), 162.9 (C-8a), 161.7 (C-4′), 157.7 (C-5), 128.9 (C-2′ and 6′), 123.1 (C-1′), 115.1 (C-3′ and 5′), 105.5 (C-4a), 104.2 (C-3), 98.9 (C-6), 93.8 (C-8), 68.9 (C-9), 56.1 (OCH_3_), and 31.4 (C-10); MS (ESI, positive) *m/z*: 392.14 [M + H]^+^.

##### 2.2.8.4 7-(4-Bromobutoxy)-5-hydroxy-2-(4-methoxyphenyl)-4H-chromen-4-one (12d)

Light yellow crystal, yield 7.5%, mp 178.9–179.8°C. ^1^H-NMR (DMSO-*d*_*6*_ and 600 MHz) δ: 12.91 (s, 1H, and 5-OH), 8.03–8.05 (m, 2H, H-2′, and 6′), 7.10–7.12 (m, 2H, H-3′, and 5′), 6.91–6.93 (m, 1H, and H-3), 6.77 (s, 1H, and H-8), 6.35–6.38 (m, 1H, and H-6), 4.06–4.34 (m, 2H, and H-9), 3.87 (m, 3H, and OCH_3_), 3.29–3.37 (m, 2H, and H-12), 1.79–2.01 (m, 4H, H-10. and 11); ^13^C-NMR (DMSO-*d*_*6*_ and 150 MHz) δ: 182.4 (C-4), 164.1 (C-2), 162.9 (C-7), 162.9 (C-8a), 161.7 (C-4′), 157.7 (C-5), 128.8 (C-2′ and 6′), 123.2 (C-1′), 115.1 (C-3′ and 5′), 105.2 (C-4a), 104.2 (C-3), 98.8 (C-6), 93.6 (C-8), 67.9 (C-9), 56.1 (OCH_3_), 30.1 (C-12), 29.9 (C-10), and 28.7 (C-11).

### 2.3 Activity Assays

#### 2.3.1 *In Vitro* Kinase Activity

*In vitro* anti-CDK1/cyclin B1 and CDK4/cyclin D3 assays were performed as described by the manufacturer. All test compounds and controls were dissolved in dimethyl sulfoxide (DMSO) to prepare 50 μM stock solutions. The stock solutions were diluted by double-distilled water and buffer to 40, 30, 20, and 10 μM working concentrations and stored at −20°C. Buffer, ATP, CDK substrate peptidase, and distilled water were mixed, and then 25 μl of the mixture was added to a 96-well plate. Five microliters of 20 μM test compounds were sequentially added. CDK1/cyclin B1 was diluted to 1 ng/μl, and CDK4/cyclin D3 was diluted to 10 ng/μl using a buffer. Twenty microliters of the kinase solutions were incubated with the test compounds at 30°C for 45 min for CDK1/cyclin B1 and for 60 min for CDK4/cyclin D3. Finally, 50 μl of Kinase-Glo Max reagent was added to each well. The 96-well plate was covered with aluminum foil and incubated at room temperature for 15 min. Luminescence of the test compounds was measured with a microplate reader. The inhibition rate can be calculated from the following formulation:

$$\text{Inhibition}\;\text{rate}==\left(1-\frac{L_{\text{sample}}-{\text{L}}_{\text{blank}}}{{\text{L}}_{\text{kinase}}-{\text{L}}_{\text{blank}}}\right)\times 100\%,$$

where L_sample_ is the luminescence of the test compound, L_blank_ is the luminescence of the blank, and L_kinase_ is the luminescence of the total kinase. The IC_50_ values were regressed according to the inhibition rates of the corresponding 50, 40, 30, 20, and 10 μM concentrations of the test compounds (Mohammed et al., 2021).

#### 2.3.2 Cell Growth Inhibition Assays

All test compounds and controls were dissolved in DMSO to prepare 250 μM stock solutions. The stock solutions were filtered through a 0.2 μM filter and diluted into five concentrations (0.1–250 μM) with DMEM medium. MCF-7 and RAW264.7 cells were inoculated at a concentration of 5,000 cells/100 μl/well into a 96-well plate for 24 h at 37°C in a humidified atmosphere containing 5% CO_2_. After 24 h of culture, the primary medium was discarded and replaced with 100 μl of fresh DMEM medium containing different concentrations of test compounds in triplicate. After another 24 h of culture, the medium was replaced with 100 μl of fresh DMEM medium containing 10% (v/v) CCK-8 reagent in each well for 2 h at 37°C. The OD value was measured at a wavelength of 450 nm. Cell viability was determined using the following equation:

$$\text{Cell}\;\text{viability}=\left(\frac{\text{ODsample}-\text{ODnegative}\;\text{control}}{\text{ODpositive}\;\text{control}-\text{ODnegative}\;\text{control}}\right)\times 100\%$$

where OD _positive control_ is the absorbance at 450 nm obtained from untreated cells. OD _negative control_ is the absorbance obtained from a blank well containing the cell culture medium and CCK-8 reagent. The IC_50_ values were calculated from the plot of activity vs. inhibitor concentration by using the GraphPad Prism 5 software (Yu et al., 2020).

### 2.4 Molecular Modeling

The molecular docking study was accomplished with the CDOCKER program of Discovery Studio to explore the predicted binding mode of compound **2a** with CDK1. The co-crystal structure of CDK1/cyclin B-Cks2 with flavopiridol (PDB: 6GU2) was obtained from the PDB database. The edge water molecules were removed, and hydrogen atoms were added to the protein by a clean protein module. The corresponding amino acids were then protonated, and energy minimization was performed. The structure of **2a** was introduced. Hydrogen atoms and CHARMm field were added. According to the binding position of flavopiridol in CDK1, a sphere with a radius of 9.0 × 10^–10^ m was set, and then flavopiridol was extracted from the complex. Flavopiridol was re-docked to the original protein by the CDOCKER program. The RMSD of the docking conformation and crystal conformation were calculated to determine the credibility of the docking results. According to the above conditions, **2a** was docked to the flavopiridol binding site of CDK1. Other docking parameters in the program were kept at default. The docking simulation using CDOCKER was prepared according to the user instructions. The binding pose figure was prepared by PyMOL (Mou et al., 2021a).

## 3 Results and Discussion

### 3.1 Chemistry

**SCHEME 1:**
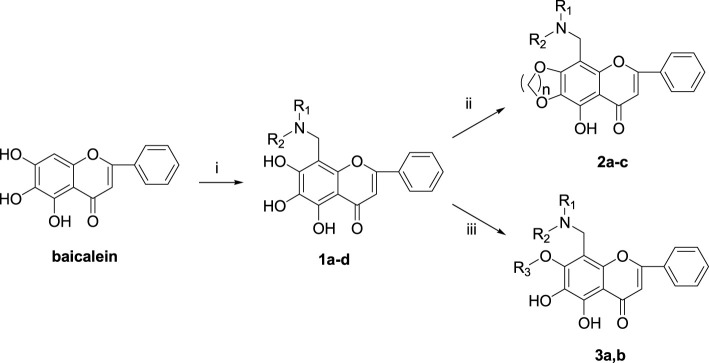
Reagents and conditions: (i) HCHO, NHR_1_R_2_/MeOH; (ii) BrCH_2_CH_2_Br, K_2_CO_3_, KI/DMF; (iii) R-Br, K_2_CO_3_, KI/DMF.

The synthetic route of baicalein derivatives is outlined in Scheme 1. In our previous work, the ether derivatives of baicalein showed good activity against MCF-7 tumor cell proliferation. Hence, more 7-substituted or 6,7-disubstituted ether derivatives of baicalein were synthesized. Compounds **1a-d** were synthesized by the Mannich reaction of baicalein with formaldehyde and four secondary amines in methanol. Compounds **2a-c** were produced by etherification of compounds **1a-c** with 1,4-dibromobutane, with the catalysis of potassium carbonate (K_2_CO_3_) and potassium iodide (KI) in *N*,*N*-dimethylformamide (DMF). Compounds **3a** and **3b** were, respectively, obtained by etherification of **1a** with bromobenzyl and **1d** with 1,4-dibromobutane in a method similar to the synthesis of **2**.

**SCHEME 2:**
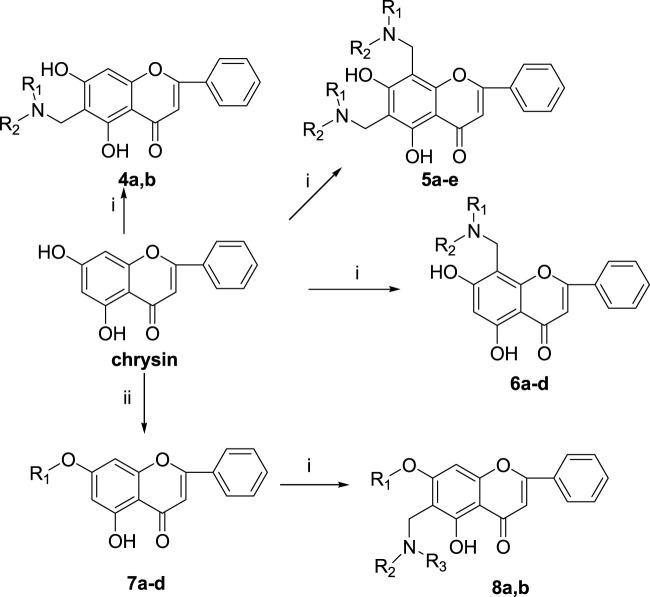
Reagents and conditions: (i) HCHO, NHR_1_R_2_/MeOH; (ii) R-Br, K_2_CO_3_, KI/DMF or (CH_3_O)_2_SO_2_, K_2_CO_3_/EtOH

The synthetic route of chrysin derivatives is outlined in Scheme 2. Compounds **4a**, **b**, **5a-e,** and **6a-d** were synthesized by the Mannich reaction of chrysin with formaldehyde and six secondary amines in methanol under different equivalent ratios. Compounds **7a**, **c**, and **d** were obtained by etherification of chrysin with three halohydrocarbons under the catalysis of K_2_CO_3_ and KI in DMF. Compound **7b** was synthesized by etherification of chrysin with dimethyl sulfate under the catalyst K_2_CO_3_ in ethanol. Compounds **8a** and **b** were synthesized by the Mannich reaction of **7b** with formaldehyde and two secondary amines in methanol.

**SCHEME 3:**
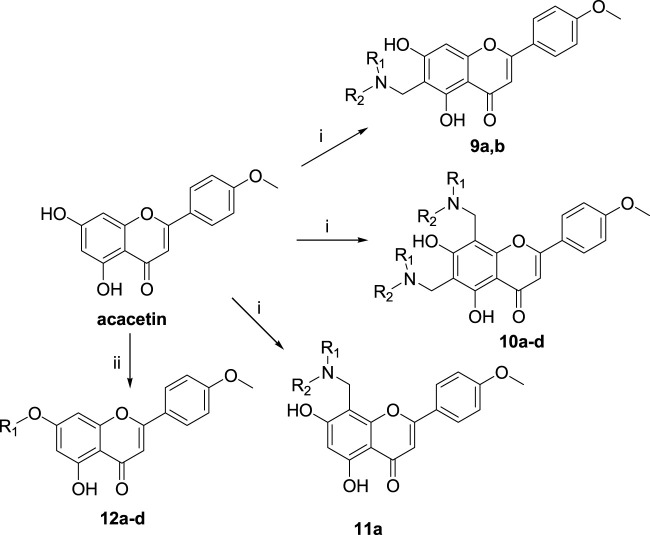
Reagents and conditions: (i) HCHO, NHR_1_R_2_/MeOH; (ii) R-Br, K_2_CO_3_, KI/DMF or (CH_3_O)_2_SO_2_, K_2_CO_3_/EtOH

The synthetic route of acacetin derivatives is outlined in Scheme 3. Compounds **9a**, and **b**, **10a-d,** and **11a** were synthesized by the Mannich reaction of acacetin with formaldehyde and five secondary amines in methanol at different equivalent ratios. Compounds **12a**, **c**, and **d** were obtained by etherification of acacetin with three halohydrocarbons under the catalysis of K_2_CO_3_ and KI in DMF. Compound **12b** was synthesized by etherification of acacetin with dimethyl sulfate under the catalyst K_2_CO_3_ in ethanol.

According to the former schemes, 4-carbonyl and 5-hydroxyl groups of flavone were retained. Thirty-seven 6-, 7-hydroxyl-, 8- and 4′-substituted flavone derivatives were synthesized (Table 1).

**TABLE 1:** The structures of flavone derivatives.

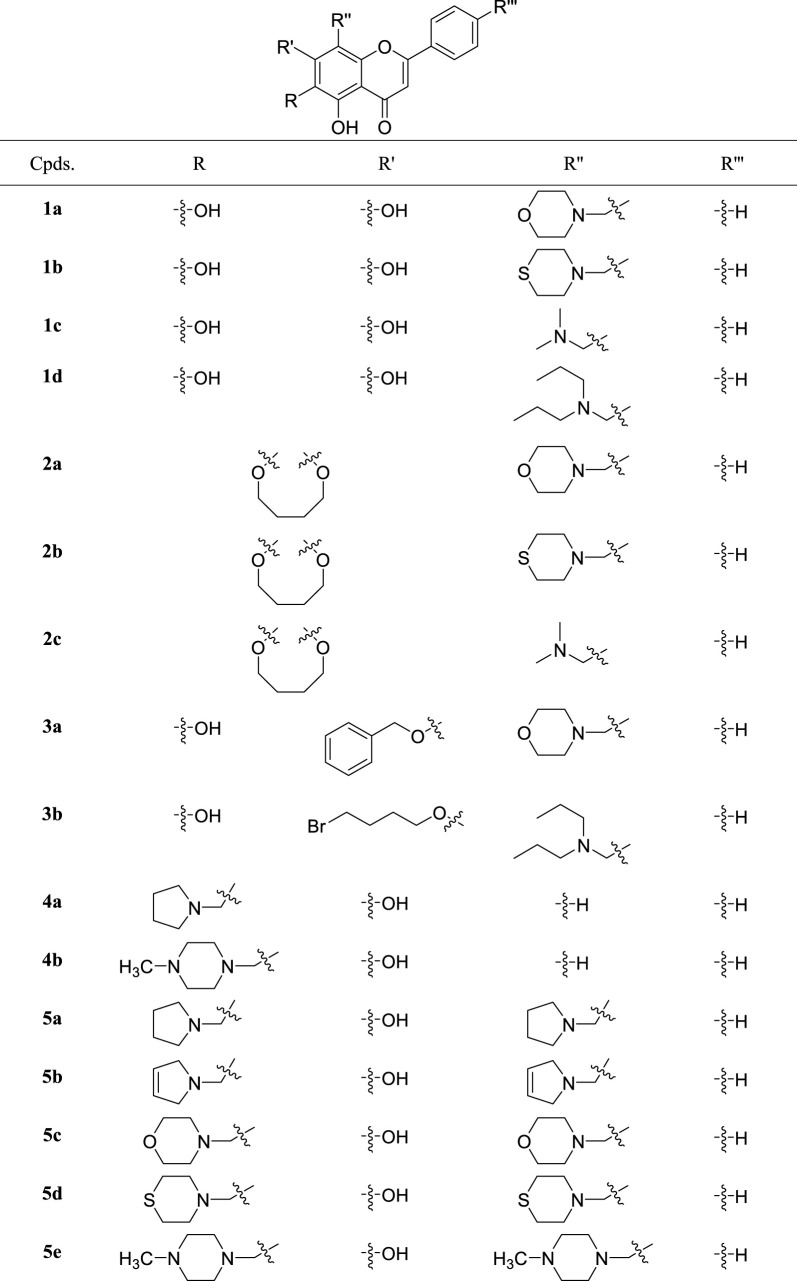	
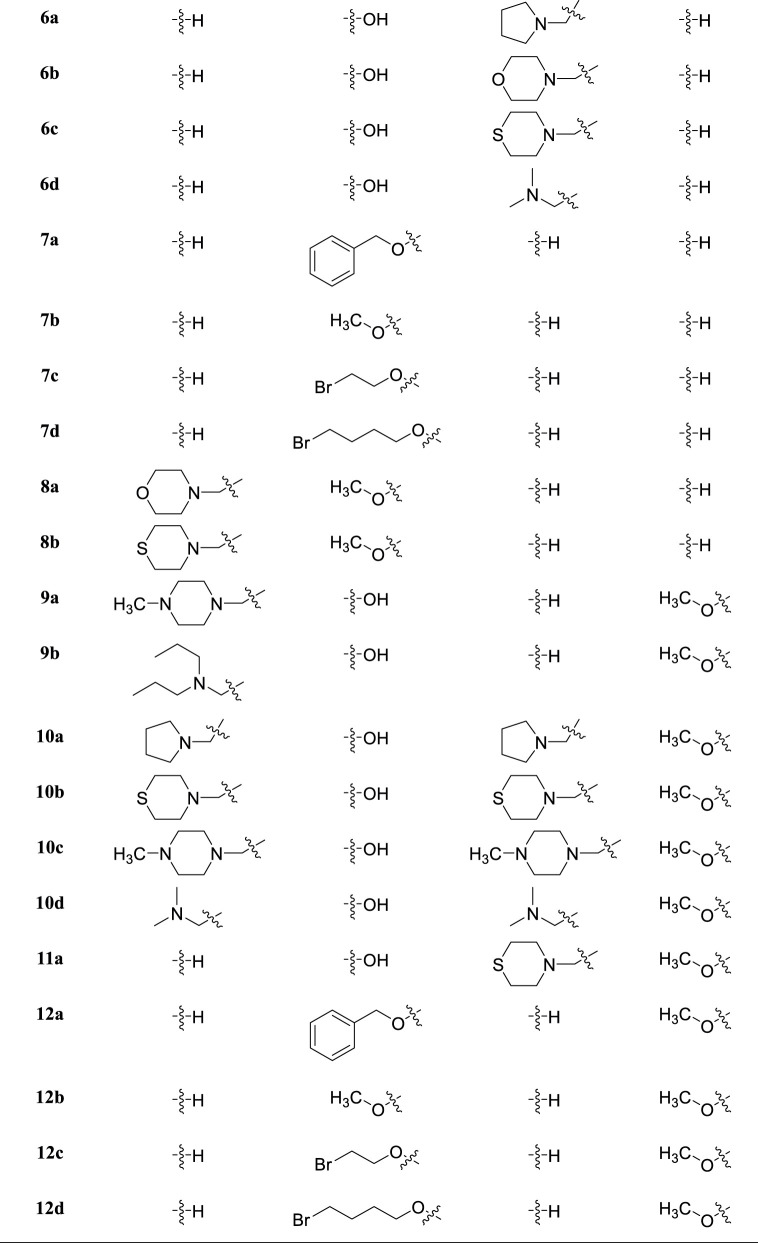	

### 3.2 Biological Evaluation

#### 3.2.1 Anti-Kinase Activity of All Target Compounds

All target compounds were evaluated for their inhibitory activities against CDK1/cyclin B1 kinase. In order to determine the compounds’ selectivity, their inhibitory activities against CDK4/cyclin D3 were also evaluated. Three lead compounds, baicalein, chrysin, and acacetin; two positive controls, flavopiridol, and CGP74514 A were used as controls. Initially, we detected the kinase inhibitory activities of all test compounds at a concentration of 20 μM. The inhibition rates are summarized in Table 2.

**TABLE 2:** Inhibition rates of all test compounds for CDK1 and CDK4.

Cpds	Inhibition rates (%)		Cpds	Inhibition rates (%)	
	CDK1	CDK4		CDK1	CDK4
**1a**	25.48 ± 0.11	6.71 ± 0.07	**7b**	21.27 ± 0.09	1.76 ± 0.05
**1b**	31.71 ± 0.08	0.09 ± 0.03	**7c**	22.74 ± 0.11	ND
**1c**	29.28 ± 0.11	ND^a^	**7d**	19.74 ± 0.11	1.83 ± 0.06
**1d**	31.11 ± 0.10	2.47 ± 0.08	**8a**	31.04 ± 0.10	ND
**2a**	33.05 ± 0.12	ND	**8b**	20.47 ± 0.08	ND
**2b**	27.19 ± 0.07	10.18 ± 0.08	**9a**	26.36 ± 0.09	5.43 ± 0.06
**2c**	29.40 ± 0.09	10.96 ± 0.09	**9b**	30.69 ± 0.10	8.55 ± 0.09
**3a**	38.11 ± 0.09	2.73 ± 0.06	**10a**	24.07 ± 0.10	4.68 ± 0.07
**3b**	25.33 ± 0.11	6.01 ± 0.07	**10b**	29.54 ± 0.11	ND
**4a**	31.72 ± 0.10	ND	**10c**	27.70 ± 0.12	8.57 ± 0.09
**4b**	28.14 ± 0.12	ND	**10d**	35.53 ± 0.08	2.73 ± 0.08
**5a**	29.00 ± 0.12	7.22 ± 0.08	**11a**	26.12 ± 0.10	ND
**5b**	28.66 ± 0.11	ND	**12a**	32.38 ± 0.11	6.00 ± 0.04
**5c**	38.34 ± 0.08	8.15 ± 0.06	**12b**	32.40 ± 0.11	7.93 ± 0.08
**5d**	30.39 ± 0.09	ND	**12c**	37.60 ± 0.12	1.43 ± 0.06
**5e**	26.75 ± 0.10	ND	**12d**	36.91 ± 0.08	3.47 ± 0.04
**6a**	35.95 ± 0.09	6.30 ± 0.07	baicalein	18.85 ± 0.09	ND
**6b**	34.47 ± 0.12	9.36 ± 0.09	chrysin	32.77 ± 0.09	ND
**6c**	34.19 ± 0.10	9.87 ± 0.09	acacetin	34.64 ± 0.11	10.44 ± 0.07
**6d**	31.24 ± 0.08	7.46 ± 0.06	flavopiridol	86.79 ± 0.04	89.71 ± 0.03
**7a**	39.51 ± 0.06	ND	CGP74514 A	62.50 ± 0.05	48.52 ± 0.04

a ND, means not determined.

Bold values are the number of the target compounds.

For the three lead compounds, the activity in descending order is acacetin, chrysin, and baicalein. Acacetin has a 4′-methoxy group on the B ring and shows the best activity compared with chrysin and baicalein. Thus, 4′-methoxy substitution is beneficial to inhibitory activity. The reason may be that the methoxy group can form hydrogen bond and hydrophobic bond with V18 and I10 in CDK1. V18 and I10 are amino acids that interact with the chlorobenzene moiety of flavopiridol. Baicalein has a 6-hydroxy group on the A ring and shows the lowest activity compared with chrysin and acacetin. It can be concluded that the existence of the 6-hydroxyl group has little effect on the inhibitory activity of CDK1. The possible reason is that 6-hydroxyl group can form an intramolecular hydrogen bond with the oxygen atom of 7-hydroxyl group and interfere with the formation of the hydrogen bond between 7-hydroxyl and E81 and L83 of CDK1.

All flavone derivatives showed better activities than baicalein, ten showed better activities than chrysin, and seven showed better activities than acacetin. However, no compounds were more active than the positive controls.

For position 6 on the flavone scaffold, the compounds with no substitution or 6,7-cyclobutyl diether possessed good activities. The compounds with Mannich derivatization at positions 6 and 8 at the same time also showed good activities. Among them, Mannich derivatization with morpholine and dimethylamine was more effective. The compounds with Mannich derivatization only at position 6 exhibited poor activities. Therefore, for improvement of CDK1 inhibitory activity, position 6 of flavone should not be modified or be modified with other positions at the same time.

For the 7-hydroxyl group on the flavone scaffold, the derivatives with etherification of the 7-hydroxyl group or simultaneous Mannich derivatization at position 8 have good activities. When position 4′ is a methoxy group, the compounds with bromoalkyl etherification in position 7 have better activity. Etherification is necessary for position 7.

For position 8 on the flavone scaffold, Mannich derivatization could markedly improve its activity. This is consistent with our previous experimental results (Mou et al., 2021a).

We selected seven flavone derivatives with good inhibition rates of CDK1, namely, three chrysin derivatives, two baicalein derivatives, and two acacetin derivatives. The anti-CDK activity assays were repeated, and IC_50_ values were calculated and listed in Table 3.

**TABLE 3:** IC_50_ values of representative compounds on CDK1 and CDK4.

Cpds	IC_50_ (μM)		Selectivity	Cpds	IC_50_ (μM)		Selectivity
	CDK1	CDK4			CDK1	CDK4	
**2a**	36.42 ± 1.12	ND^a^	ND	**7a**	54.97 ± 1.45	130.04 ± 2.84	2.37
**5c**	39.56 ± 1.43	508.50 ± 5.56	12.85	baicalein	62.67 ± 1.52	354.27 ± 5.67	5.65
**3a**	42.23 ± 1.62	543.78 ± 4.83	12.88	chrysin	36.63 ± 1.23	224.52 ± 3.46	6.13
**12c**	43.64 ± 1.53	ND	ND	acacetin	31.93 ± 1.08	206.09 ± 3.61	6.45
**12d**	51.43 ± 1.56	ND	ND	flavopiridol	11.49 ± 0.56	ND	ND
**6a**	51.43 ± 1.68	228.61 ± 4.55	4.45	CGP74514 A	10.86 ± 0.89	35.58 ± 1.02	3.28

a ND, means not determined.

Bold values are the number of the target compounds.

All of the compounds exhibited obvious selectivity with CDK1 compared with CDK4. Compound **2a** showed the best activity (IC_50_ = 36.42 ± 1.12 μM vs. 11.49 ± 0.56 μM of flavopiridol). The compounds with Mannich derivatization on position 8, along with cyclic etherification on positions 6 and 7, or Mannich derivatization on position 6, or etherification on position 7 exhibited good anti-CDK1 activities.

In summary, for the structural modification of flavone, the introduction of 4′-methoxy, Mannich derivatization at position 8, Mannich derivatization, and etherification at positions 6 and 7, respectively, or cyclic etherification at the same time, will help to improve the activities of the target compounds to CDK1.

#### 3.2.2 Anti-Proliferation Activity Assays of Representative CDK1 Inhibitors

Three representative compounds with excellent CDK1 inhibitory activity were chosen for the *in vitro* anti-proliferation assays against MCF-7 and RAW264.7 cells. The results were summarized in Table 4.

**TABLE 4:** IC_50_ values of the test compounds on MCF-7 and RAW264.7 cells.

Cpds	IC_50_ (μM)	
	MCF-7	RAW264.7
**2a**	79.72 ± 1.81	2.54 ± 0.12
**5c**	76.86 ± 1.47	59.43 ± 1.23
**12c**	130.65 ± 2.35	68.36 ± 0.99
flavopiridol	294.13 ± 3.13	0.55 ± 0.05

All of the test compounds showed better activities toward RAW264.7 than MCF-7 cells. To MCF-7 tumor cells, all of the representative compounds showed better activity than flavopiridol (IC_50_ = 294.13 ± 3.13 μM). Compounds **2a** and **5c** with morpholine ring in their structures had comparative anti-proliferation activities to MCF-7 cells (IC_50_ = 79.72 ± 1.81 and 76.86 ± 1.47 μM, respectively). This fact further verifies the importance of the morpholine ring in flavone derivatization. In RAW264.7 cells, all of the representative compounds showed excellent activities, but their activities were still weaker than that of flavopiridol (IC_50_ = 0.55 ± 0.05 μM). Compound **2a** had the best activity (IC_50_ = 2.54 ± 0.12 μM) compared with the other two derivatives and considerable activity to flavopiridol.

From the above results, flavone derivatives can inhibit the proliferation of both MCF-7 and RAW264.7 cells. This indicates that besides preventing the growth of tumor cells by inhibiting CDKs, flavone derivatives can also inhibit the growth of tumor cells by participating in the inflammatory response. Compound **2a** exhibited satisfactory activity in CDK1 inhibition and tumor cell proliferation in our previous and current work. Furthermore studies on it will be conducted soon.

### 3.3 Molecular Docking Study

**FIGURE 4:**
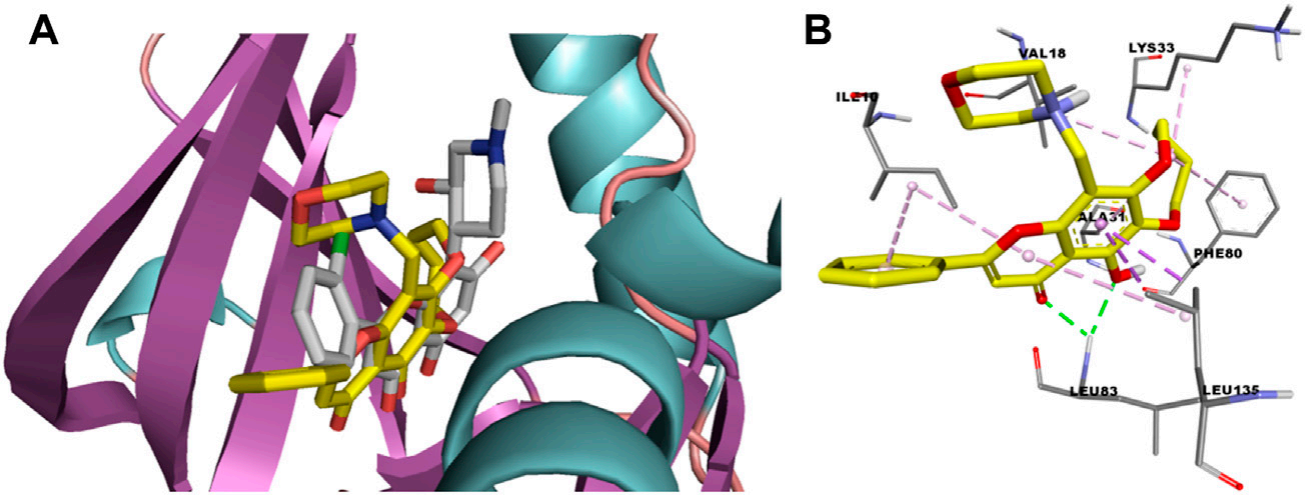
**(A)** Compounds **2a** and flavopiridol bind to the ATP binding site of CDK1 (**2a** in yellow, while flavopiridol in pale). **(B)** Binding mode of **2a** with the key amino acid residues of CDK1.

Compound **2a** showed excellent activities in the anti-kinase activity assay and anti-proliferation activity assay. In order to explore the binding mode of the flavone derivatives, molecular docking of compound **2a** with CDK1/cyclin B-Cks2-flavopiridol co-crystal complex (PDB: 6GU2) was carried out. A random conformation search was done to identify predicted ligand-protein binding conformations that are closer to the crystal ones. The RMSD of flavopiridol docking conformation compared with its crystal conformation was 1.3209. The CDOCKER energy and docking score of **2a** were -9.7 kcal mol^−1^ and 140.61, respectively. These results indicated that **2a** can bind to 6GU2 steadily. From the docking results, it can also be found that **2a** is sandwiched between A31 and L135, just like flavopiridol. But the binding conformation of **2a** with CDK1 exposed slightly more outward than that of flavopiridol (Figure 4). 4-Carbonyl and 5-phenolic hydroxyl of **2a** forms two hydrogen bonds with L83, while the corresponding functional groups in flavopiridol form a hydrogen bond with L83 and E81, respectively. 6,7-Cyclobutyl diether forms hydrophobic interaction with F80, and in flavopiridol, the A ring of the chromone core does so. 6,7-Cyclobutyl diether could also form networks of interactions with K33 and V18, while the piperidinol moiety of flavopiridol forms interaction with K33 and the chlorophenyl group forms interaction with V18. The B ring of **2a** interacts with I10. In flavopiridol, the chlorophenyl group not only interacts with V18 but also interacts with I10. Finally, the 8-morpholinomethyl group of **2a** can interact with V18. Because **2a** can form hydrogen bonds and various hydrophobic interactions with the key amino acid residues of CDK1 (Figure 4), it shows an excellent inhibitory effect on CDK1.

## 4 Conclusion

According to the binding mode of flavopiridol with CDK1, a series of CDK1 inhibitors with the flavone scaffolds were designed and synthesized. In the *in vitro* anti-kinase activity assay, all compounds exhibited excellent selectivity for CDK1 compared with CDK4 but no compounds were more active than the positive controls. Compound **2a** showed the best activity. For the compounds with a flavone scaffold, the introduction of 4′-methoxy, Mannich derivatization at position 8, Mannich derivatization, and etherification at positions 6 and 7 or cyclic etherification at the same time, will help to improve the activities of the parent compounds to CDK1. In the *in vitro* cell viability assay, all three compounds showed better activity toward RAW264.7 than toward MCF-7 cells. To MCF-7 cells, all of the representative compounds showed better activity than flavopiridol. Compounds **2a** and **5c** had comparable activities. In RAW264.7 cells, **2a** showed the best activity, but the activity was still weaker than that of flavopiridol. Anti-cell proliferation assays indicated that our target compounds, besides preventing the growth of tumor cells by inhibiting CDKs, can also inhibit the growth of tumor cells by participating in the inflammatory response. Molecular docking results of **2a** with CDK1 suggested that **2a** can form hydrogen bonds and various hydrophobic interactions with the key amino acid residues of CDK1. It can be used as a promising lead compound for CDK1 inhibitor development.

## Data Availability

The original contributions presented in the study are included in the article/Supplementary Material; further inquiries can be directed to the corresponding author.
