# Supplementary material for: Design, Synthesis, and Activity Assays of Cyclin-Dependent Kinase 1 Inhibitors With Flavone Scaffolds
Source: Front Chem. 2022 Aug 8;10:940427. doi: 10.3389/fchem.2022.940427 (PMC9393241; doi:10.3389/fchem.2022.940427)
Supplement: Supplementary file 1 [file DataSheet1.PDF]

Supplementary Material

Contents

1. Experimental Procedures………………………………………………..2-6

2. Analytical data ………………………………………………………9-43

1. Experimental Procedures

1.1 Synthesis of compounds 1

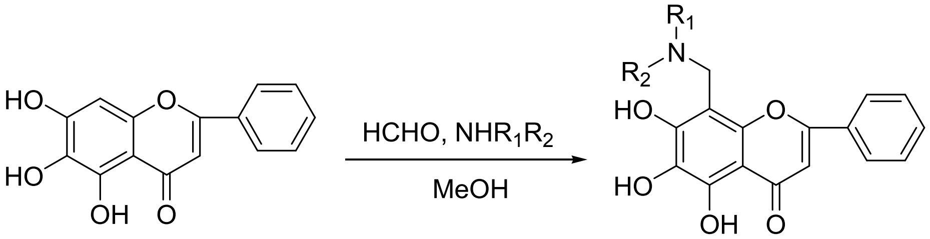

baicalein 1

Formaldehyde solution (37%, 6 mmol) was added to baicalein (4 mmol) in methanol (30 mL), followed by one of the four secondary amines (4.8 mmol). The mixture was stirred at 30–70 °C until a large amount of yellow precipitate appeared. The precipitate was filtered under vacuum, washed with a small amount of methanol and dried in a vacuum oven to obtain one of the 5,6,7-trihydroxy-2-phenyl-8-aminomethylene-4H-chromen-4-ones (**1**).

1.2 Synthesis of compounds 2

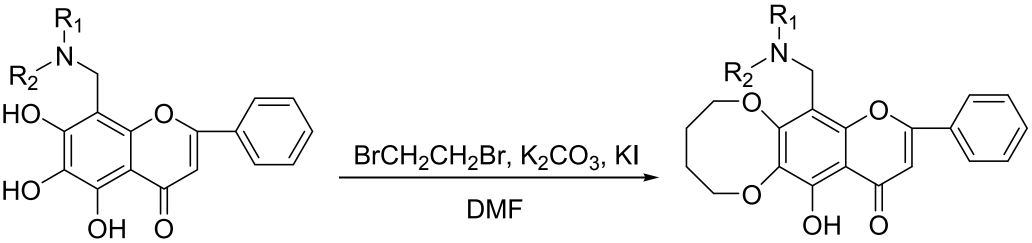

1 2

To a solution of compound **1a**, or **1b**, or **1c** (4 mmol) in 30 mL DMF was added 1, 4-dibromobutane (5.2 mmol, 0.62 mL), potassium carbonate (12 mmol) and potassium iodide (12 mmol) successively. The mixture was stirred for 7 h under nitrogen atmosphere at 60-70 °C. After that, the mixture was filtered under vacuum and a few drops of formic acid were added. The filtrate was then evaporated under vacuum and dispersed in cold water to form a suspension. The suspension was neutralized and filtered to obtain the crude product. The crude product was purified by silica gel column chromatography and recrystallization to get compounds **2**.

1.3 Synthesis of compounds 3

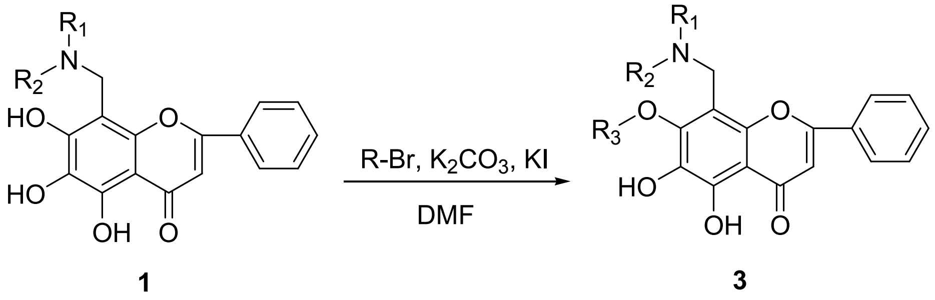

To a solution of **1a** or **1d** (4 mmol) in 30 mL DMF was added bromobenzyl or 1,4-dibromobutane (5.2 mmol), K_2CO_3 (12 mmol) and KI (12 mmol) successively. The mixture was stirred for 7 h under nitrogen atmosphere at 60-70°C. After that, the mixture was filtered under vacuum and a few drops of formic acid were added. The filtrate was then evaporated under vacuum and dispersed in cold water to form a suspension. The suspension was neutralized and filtered to obtain the crude product. The crude product was purified by silica gel column chromatography and recrystallization to get compounds **3**.

1.4 Synthesis of compounds 4, 5, and 6

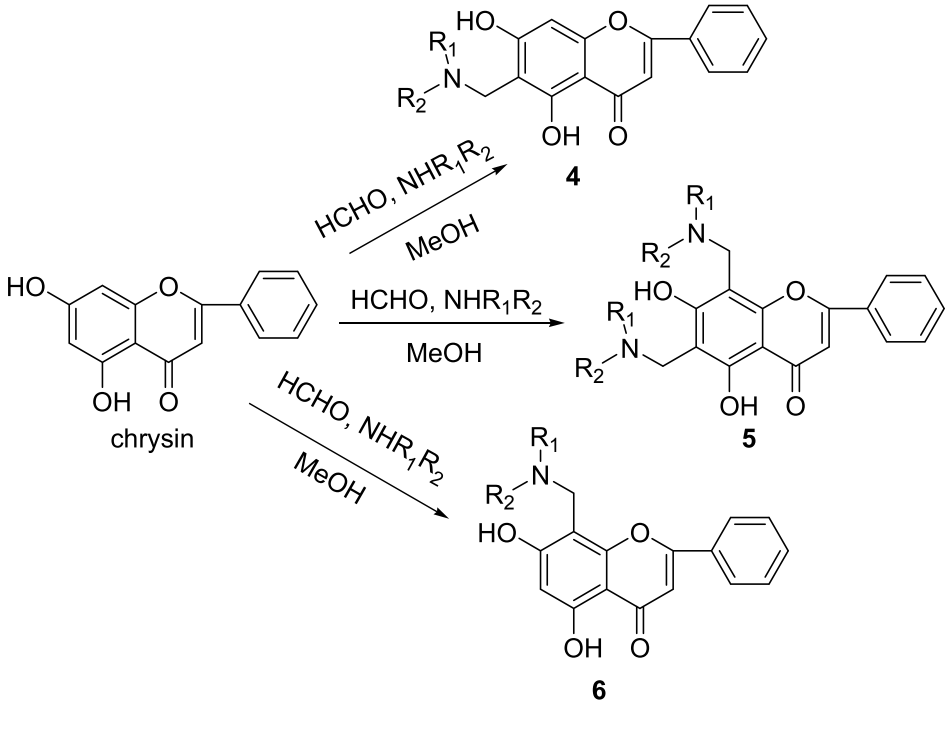

Formaldehyde solution (37%, 6 mmol) was added to chrysin (4 mmol) in methanol (30 mL), followed by one of the six secondary amines (4.8 mmol). The mixture was stirred at 30–70 °C until a large amount of yellow precipitate appeared. The precipitate was filtered under vacuum, washed with a small amount of methanol and dried in a vacuum oven to obtain the crude product. The crude product was purified by silica gel column chromatography and recrystallization to get compounds **4**, **5**, and **6**.

1.5 Synthesis of compounds 7

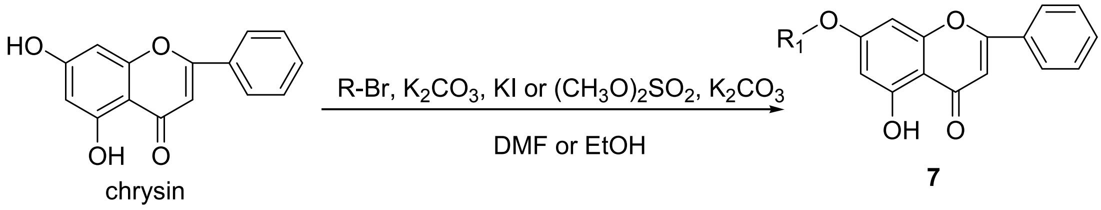

To a solution of chrysin (4 mmol) in 30 mL DMF was added bromobenzyl or 1,2-dibromoethane or 1,4-dibromobutane (5.2 mmol), K_2CO_3 (12 mmol) and KI (12 mmol) successively. The mixture was stirred for 7 h under nitrogen atmosphere at 60-70°C. After that, the mixture was filtered under vacuum and a few drops of formic acid were added. The filtrate was then evaporated under vacuum and dispersed in cold water to form a suspension. The suspension was neutralized and filtered to obtain the crude product. The crude product was purified by silica gel column chromatography and recrystallization to get compounds **7a**, **c**, **d**.

To a solution of chrysin (2 mmol) in 100 mL ethanol was added dimethyl sulfate (4 mmol), K_2CO_3 (10 mmol), successively. The mixture was stirred for 4 h under nitrogen atmosphere at 65°C. After that, the mixture was filtered under vacuum and a few drops of formic acid were added. The filtrate was then evaporated under vacuum to obtain the crude product. The crude product was purified by silica gel column chromatography and recrystallization to get compounds **7b**.

1.6 Synthesis of compounds 8

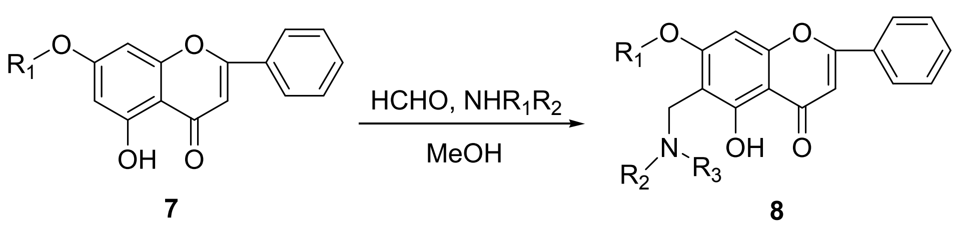

Formaldehyde solution (37%, 6 mmol) was added to compound **7b** (4 mmol) in methanol (30 mL), followed by one of the two secondary amines (4.8 mmol). The mixture was stirred at 30–70 °C until a large amount of yellow precipitate appeared. The precipitate was filtered under vacuum, washed with a small amount of methanol and dried in a vacuum oven to obtain compounds **8**.

1.7 Synthesis of compounds 9, 10, and 11

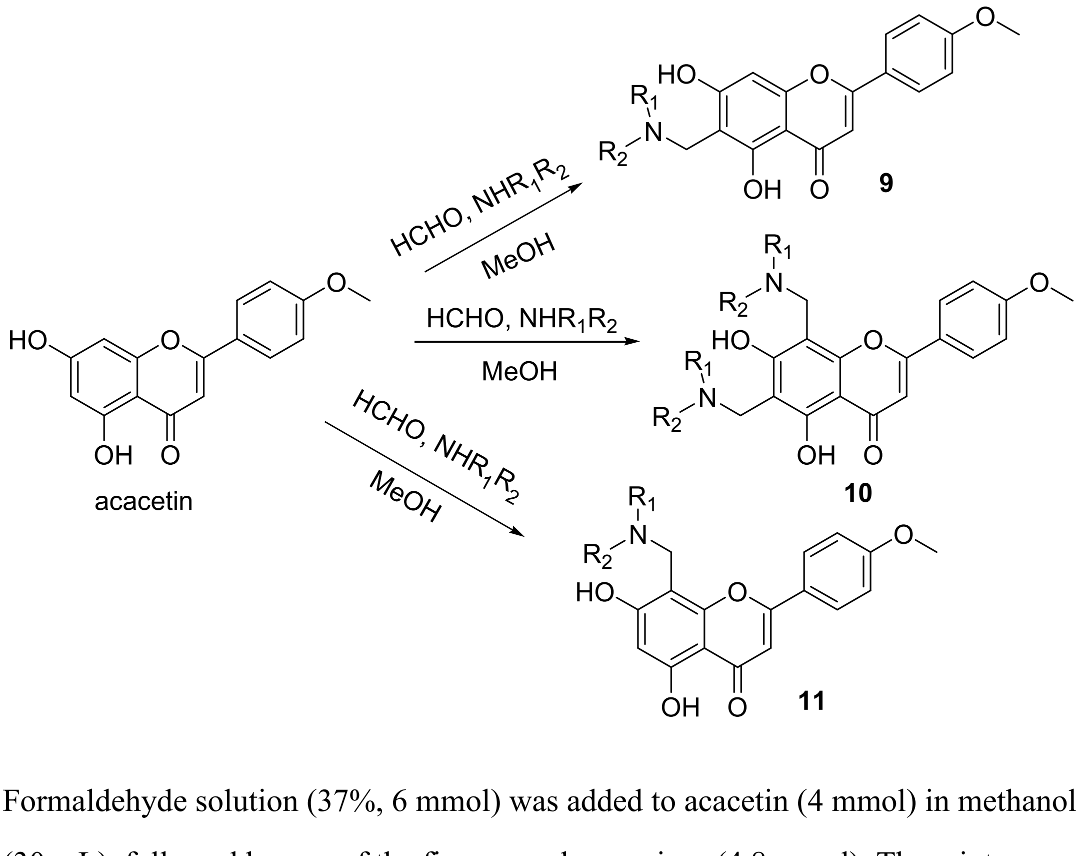

Formaldehyde solution (37%, 6 mmol) was added to acacetin (4 mmol) in methanol (30 mL), followed by one of the five secondary amines (4.8 mmol). The mixture was stirred at 30–70 °C until a large amount of yellow precipitate appeared. The precipitate was filtered under vacuum, washed with a small amount of methanol and dried in a vacuum oven to obtain the crude product. The crude product was purified

by silica gel column chromatography and recrystallization to get compounds **9**, **10**, and **11**.

1.8 Synthesis of compounds 12

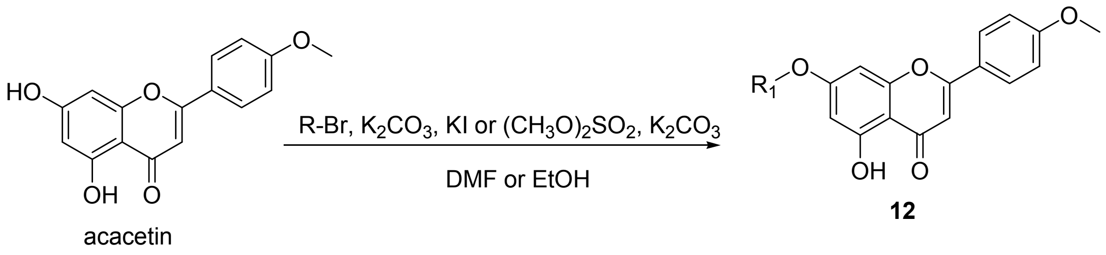

To a solution of acacetin (4 mmol) in 30 mL DMF was added bromobenzyl or 1,2-dibromoethane or 1,4-dibromobutane (5.2 mmol), K_2CO_3 (12 mmol) and KI (12 mmol) successively. The mixture was stirred for 7 h under nitrogen atmosphere at 60-70°C. After that, the mixture was filtered under vacuum and a few drops of formic acid were added. The filtrate was then evaporated under vacuum and dispersed in cold water to form a suspension. The suspension was neutralized and filtered to obtain the crude product. The crude product was purified by silica gel column chromatography and recrystallization to get compounds **12a, c, d**.

To a solution of acacetin (2 mmol) in 100 mL ethanol was added dimethyl sulfate (4 mmol), K_2CO_3 (10 mmol), successively. The mixture was stirred for 4 h under nitrogen atmosphere at 65°C. After that, the mixture was filtered under vacuum and a few drops of formic acid were added. The filtrate was then evaporated under vacuum to obtain the crude product. The crude product was purified by silica gel column chromatography and recrystallization to get compounds **12b**.

2. Analytical data

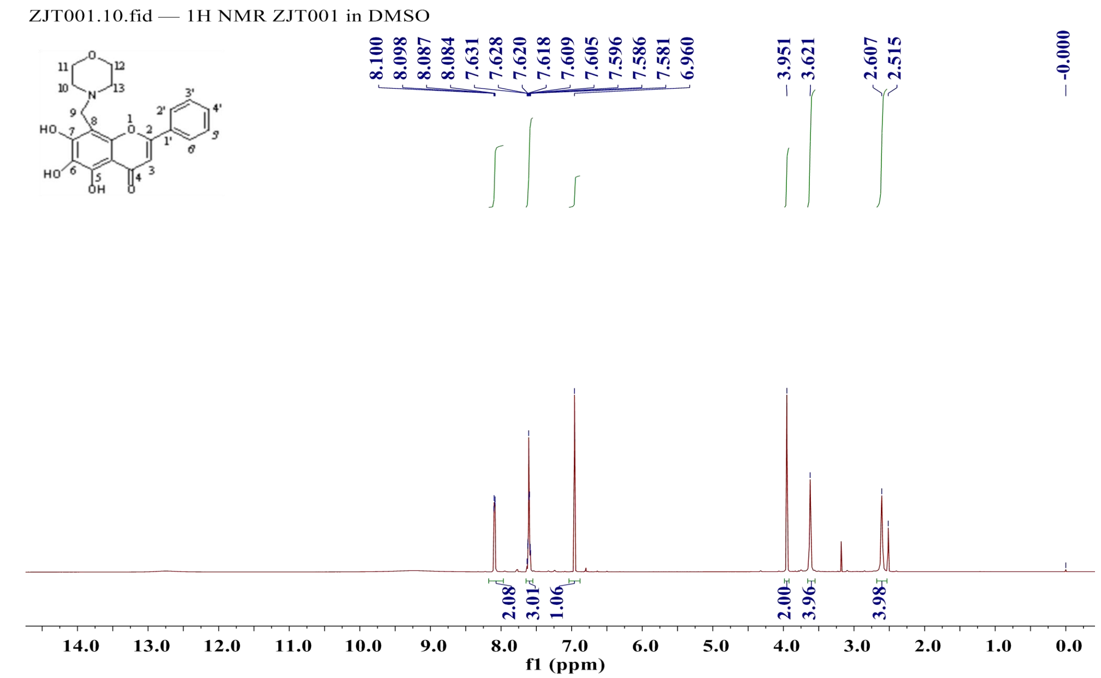

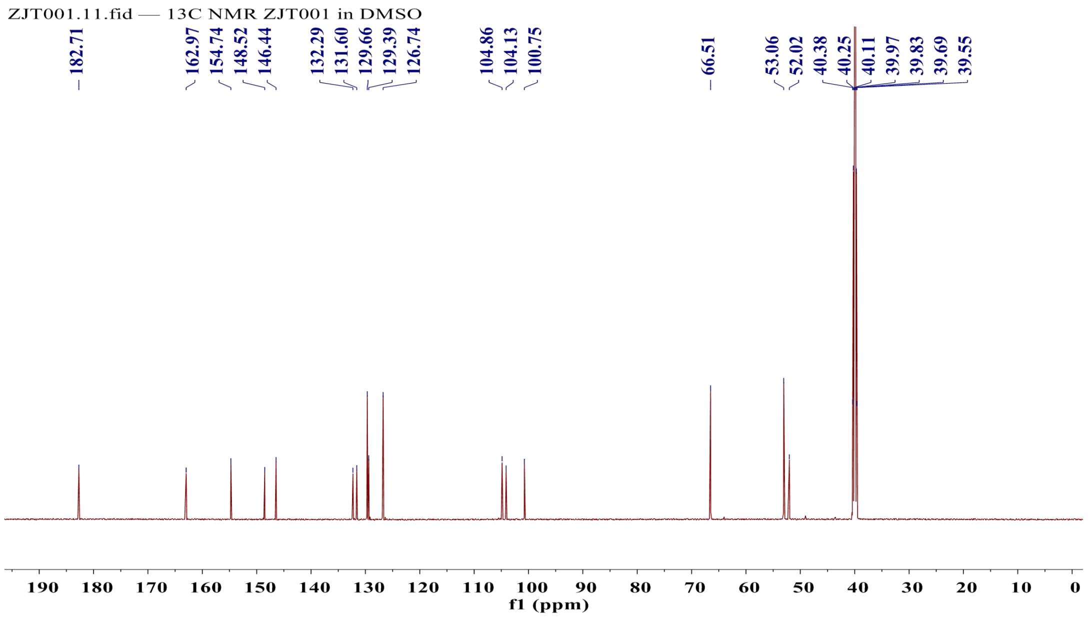

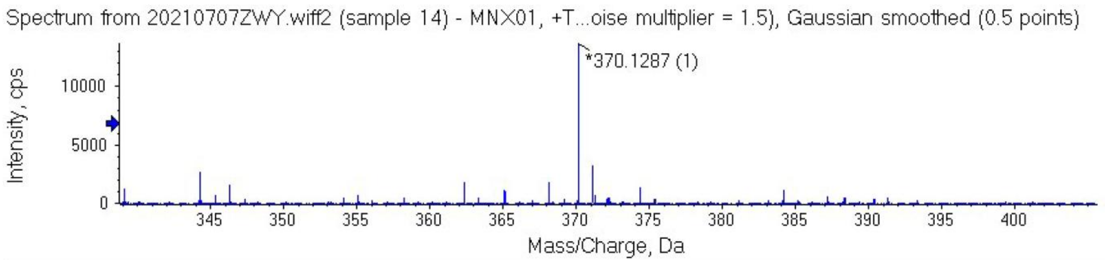

The ^{1}H-NMR, ^{13}C-NMR and HRMS spectra of compound **1a**

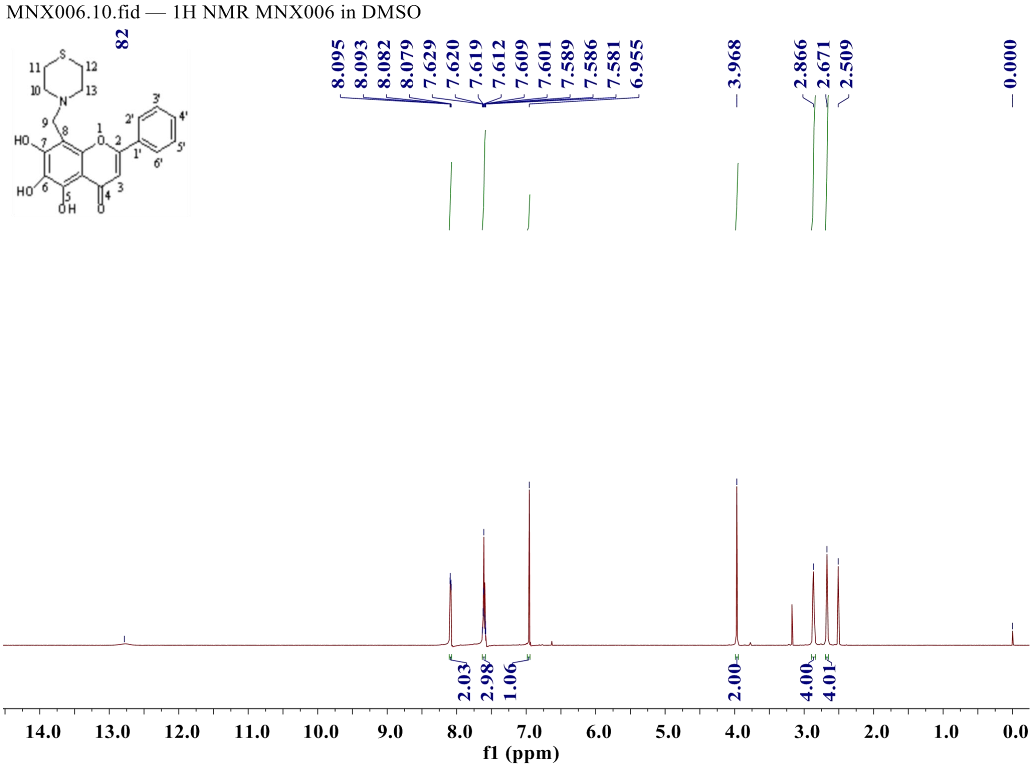

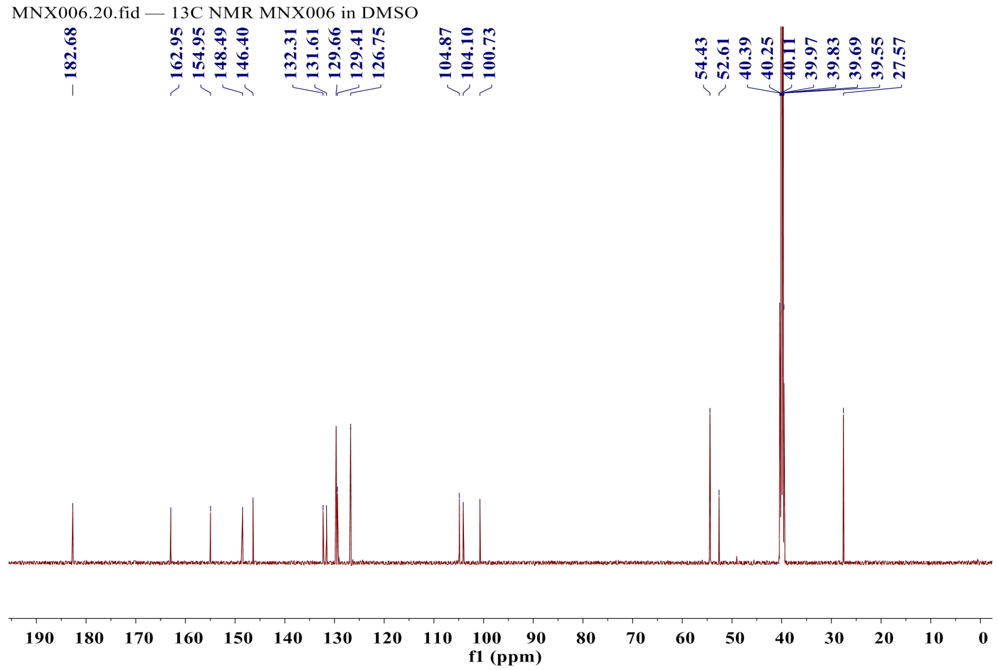

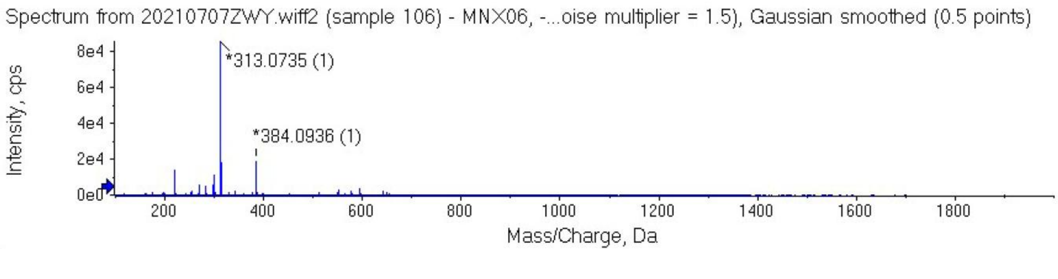

The ^{1}H-NMR, ^{13}C-NMR and HRMS spectra of compound **1b**

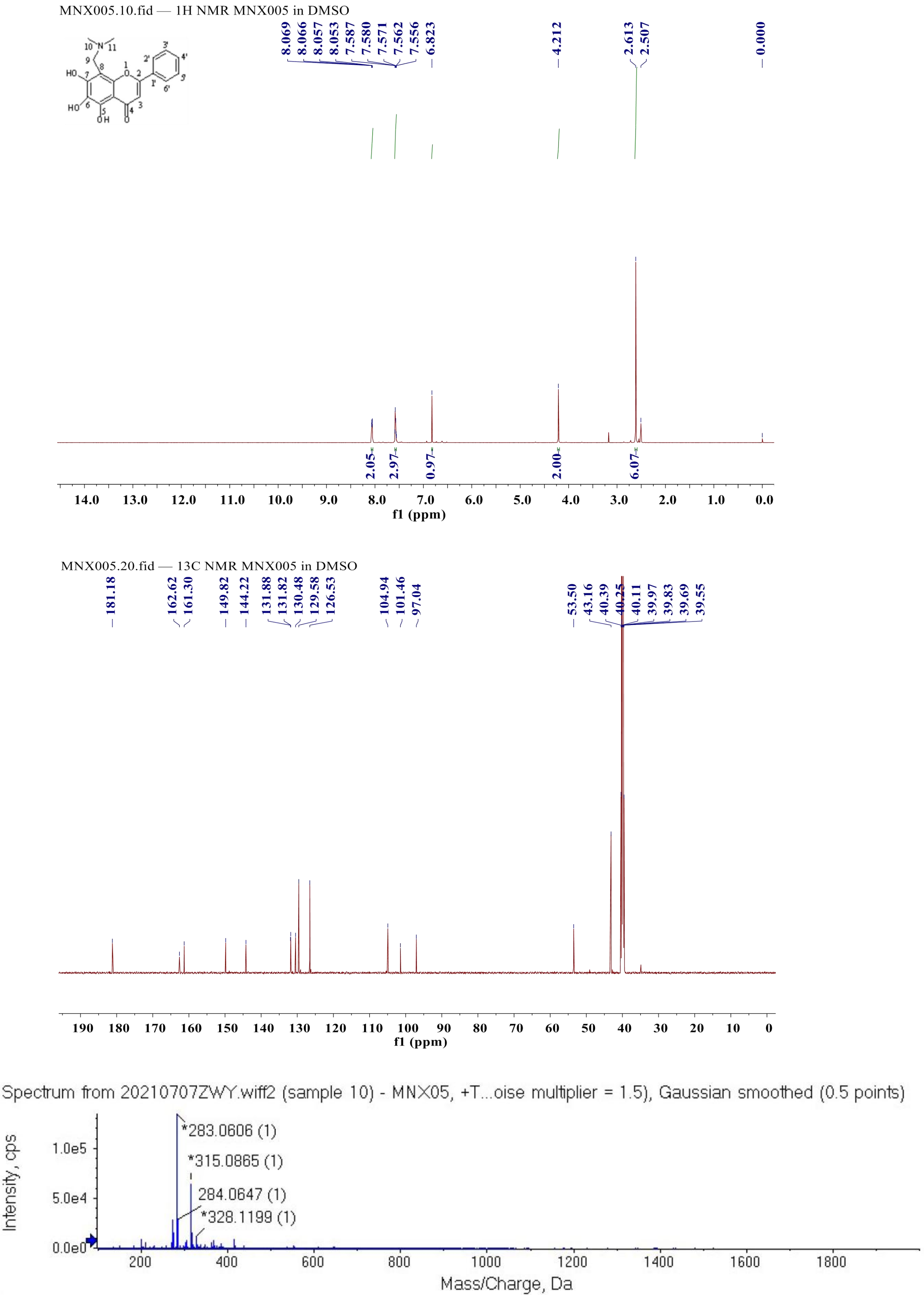

The ^{1}H-NMR, ^{13}C-NMR and HRMS spectra of compound **1c**

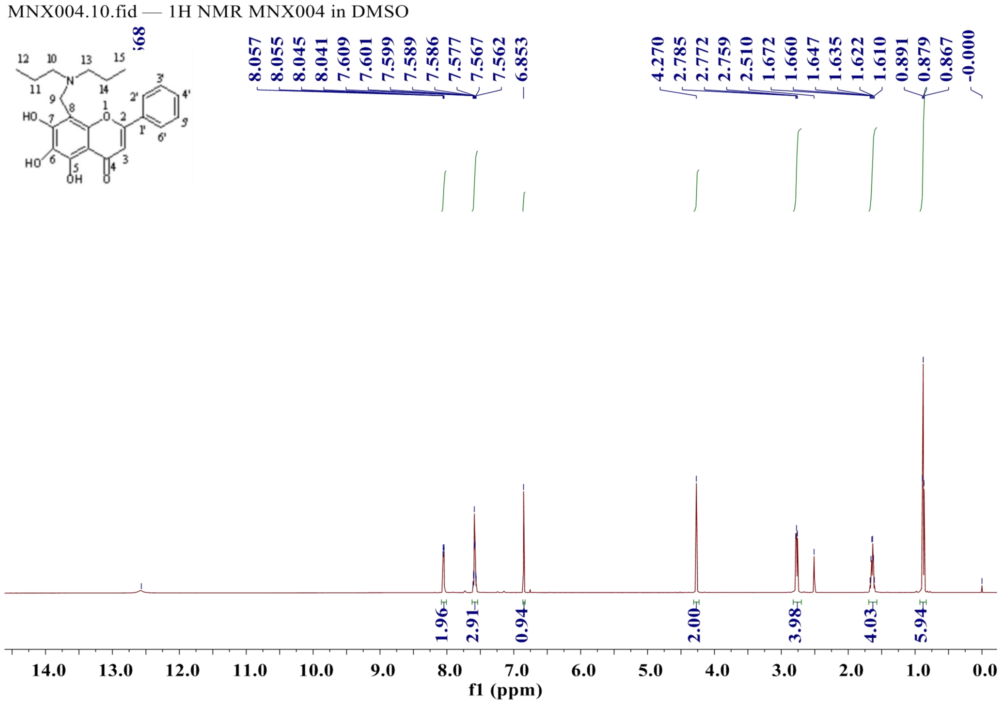

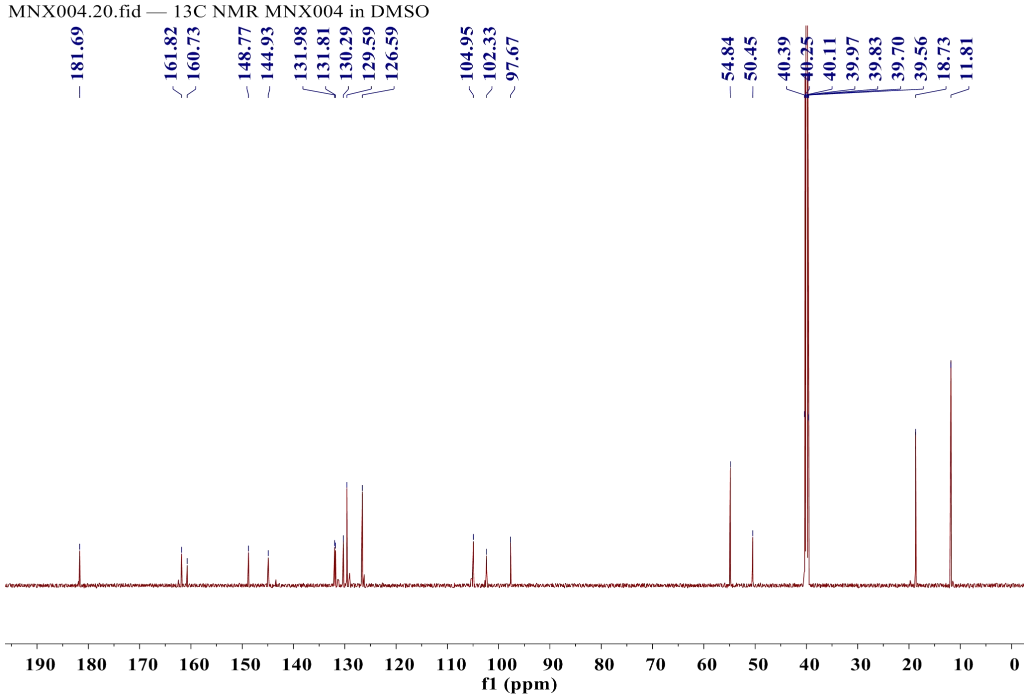

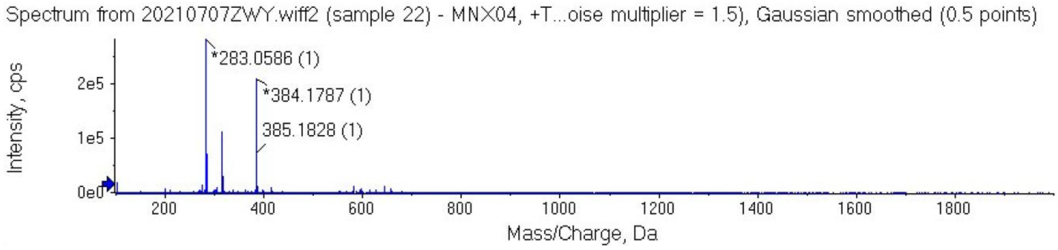

The ^{1}H-NMR, ^{13}C-NMR and HRMS spectra of compound **1d**

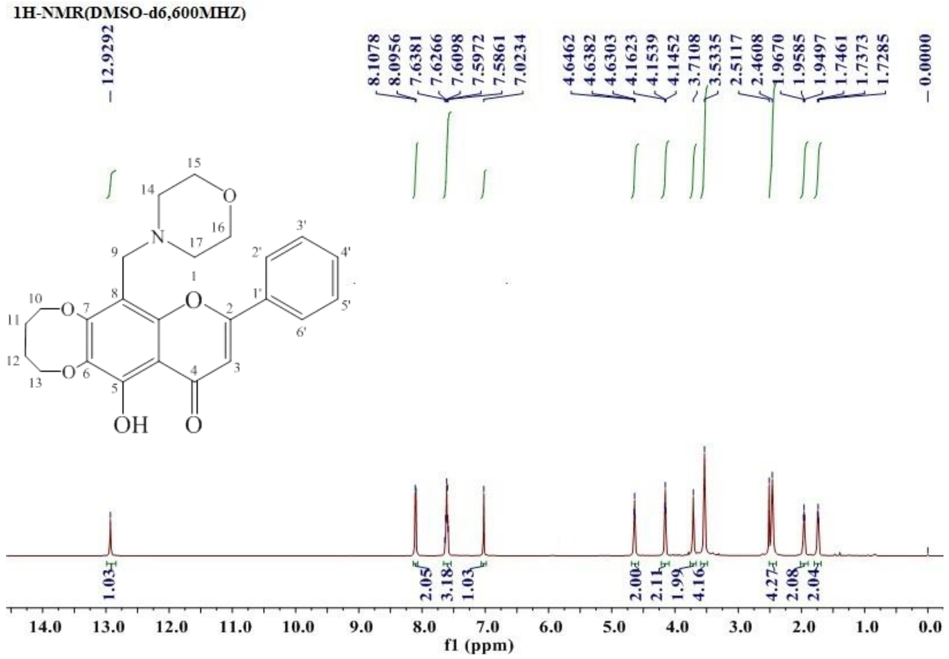

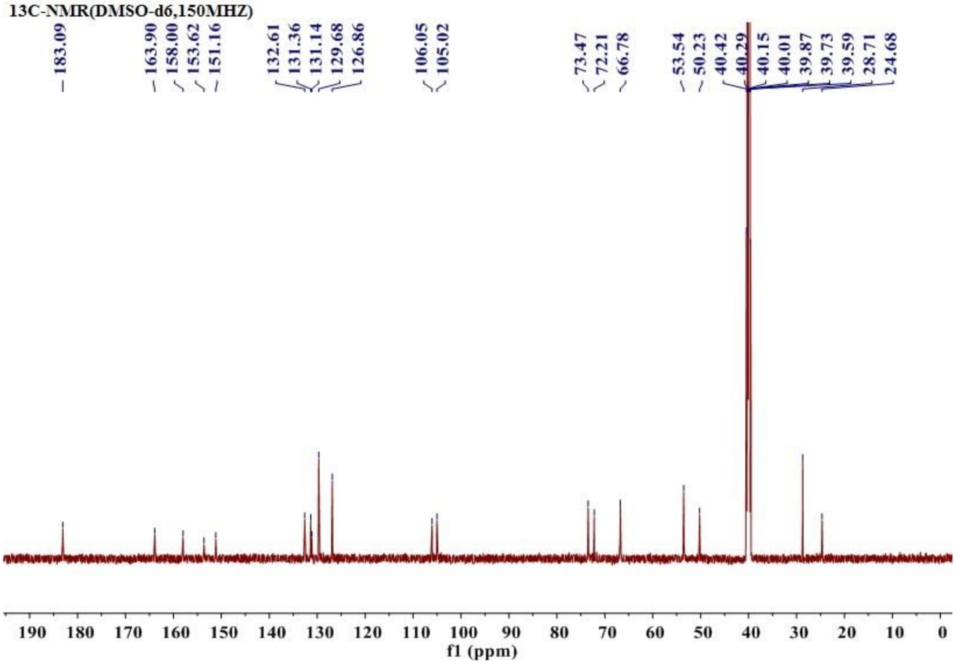

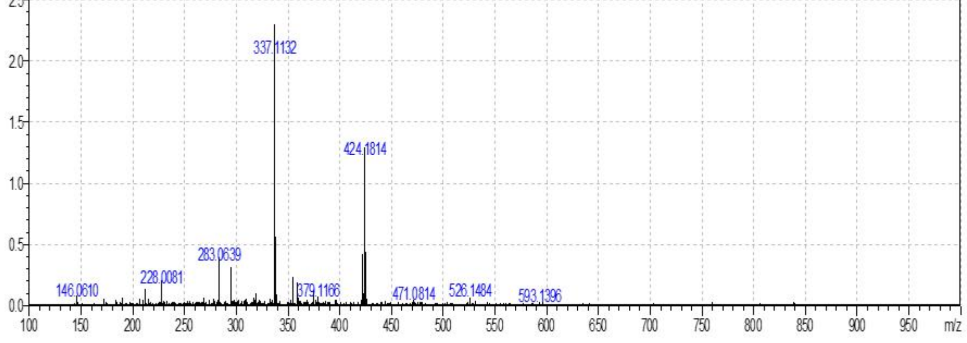

The ^{1}H-NMR, ^{13}C-NMR and HRMS spectra of compound **2a**

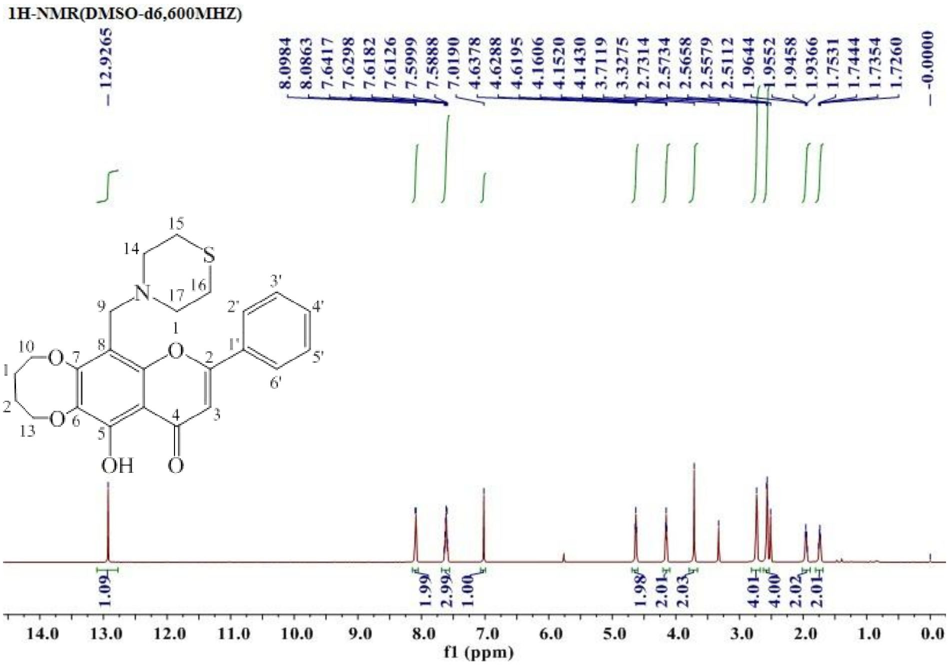

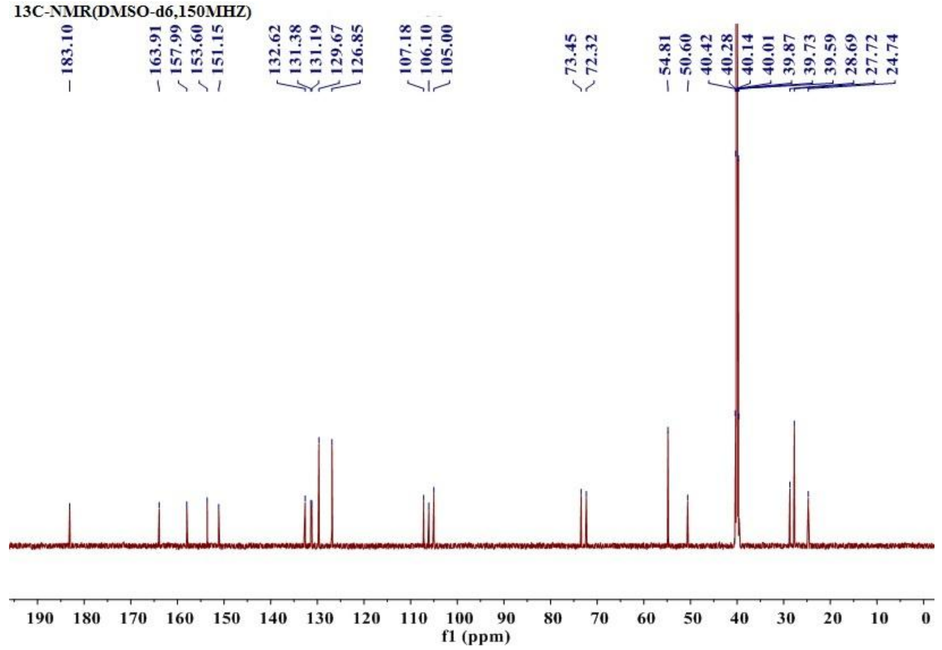

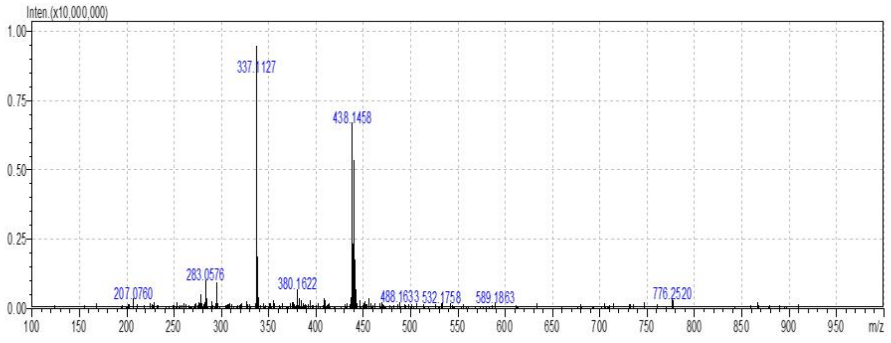

The ^{1}H-NMR, ^{13}C-NMR and HRMS spectra of compound **2b**

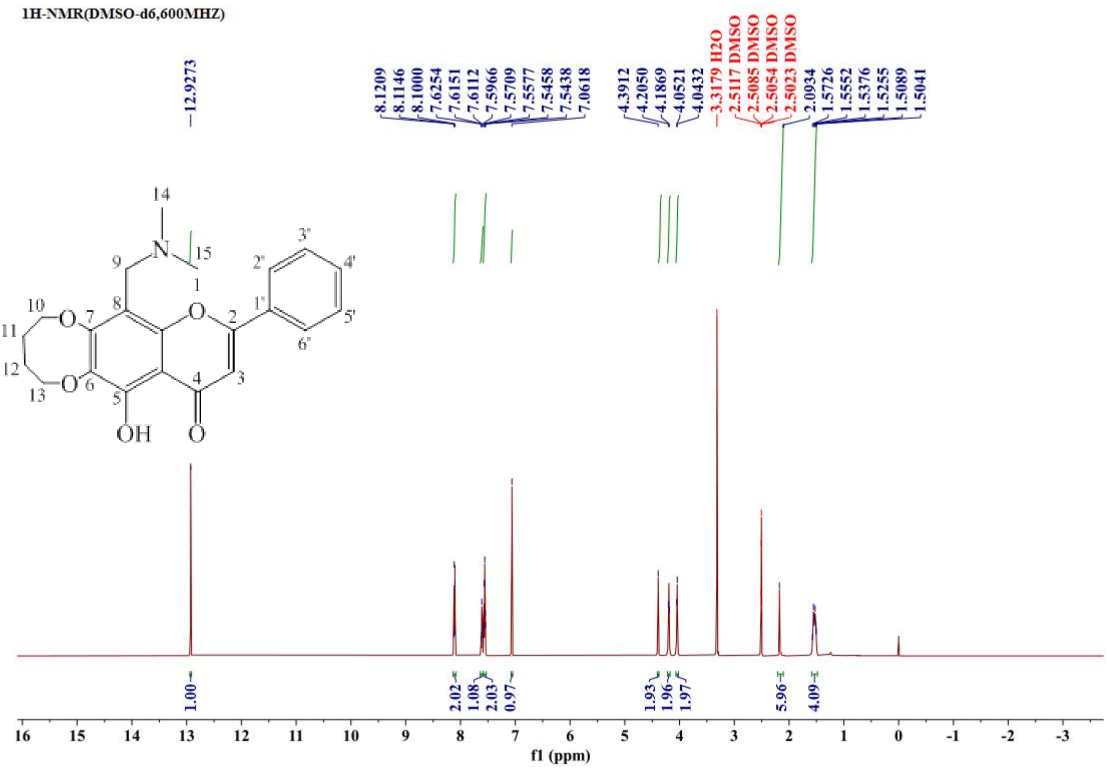

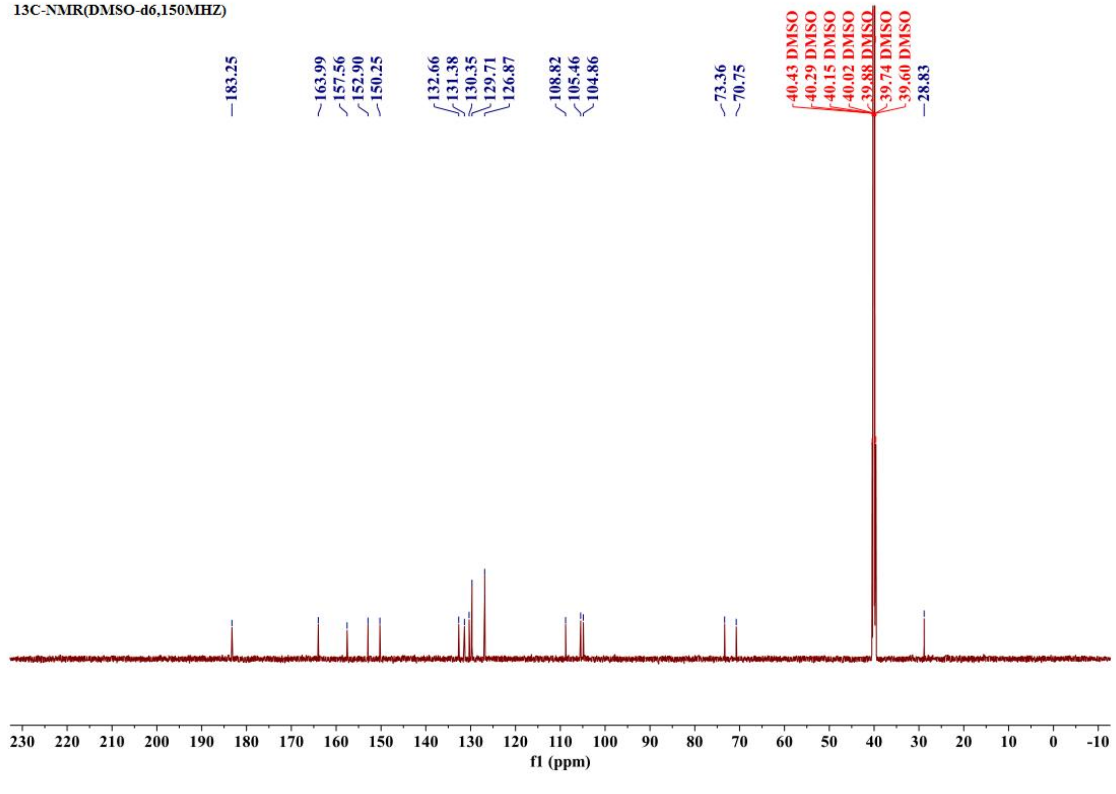

The ^{1}H-NMR and ^{13}C-NMR spectra of compound **2c**

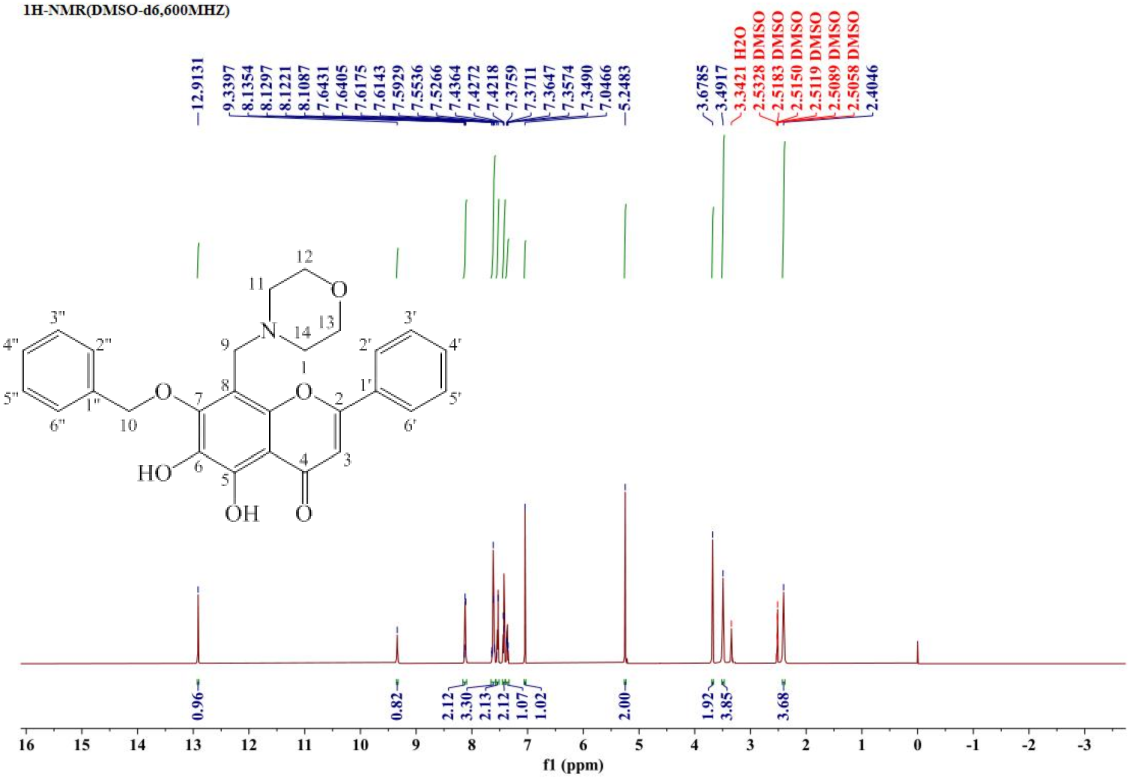

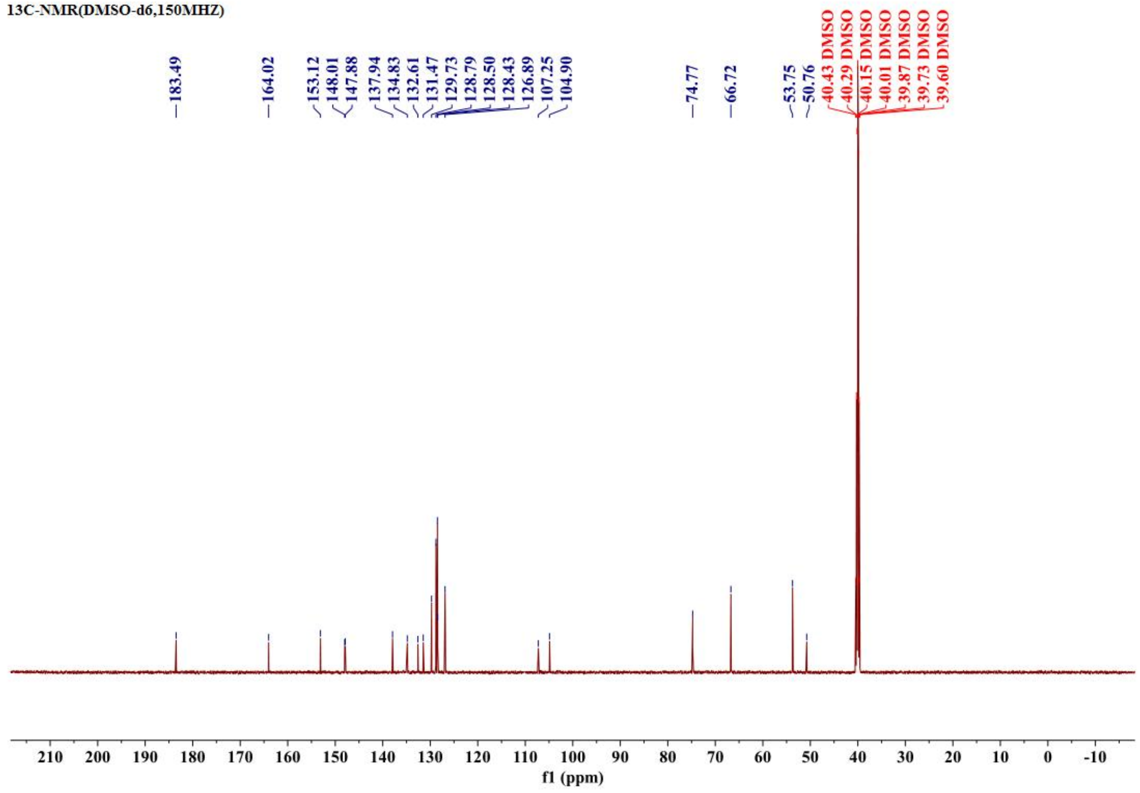

The ^{1}H-NMR and ^{13}C-NMR spectra of compound **3a**

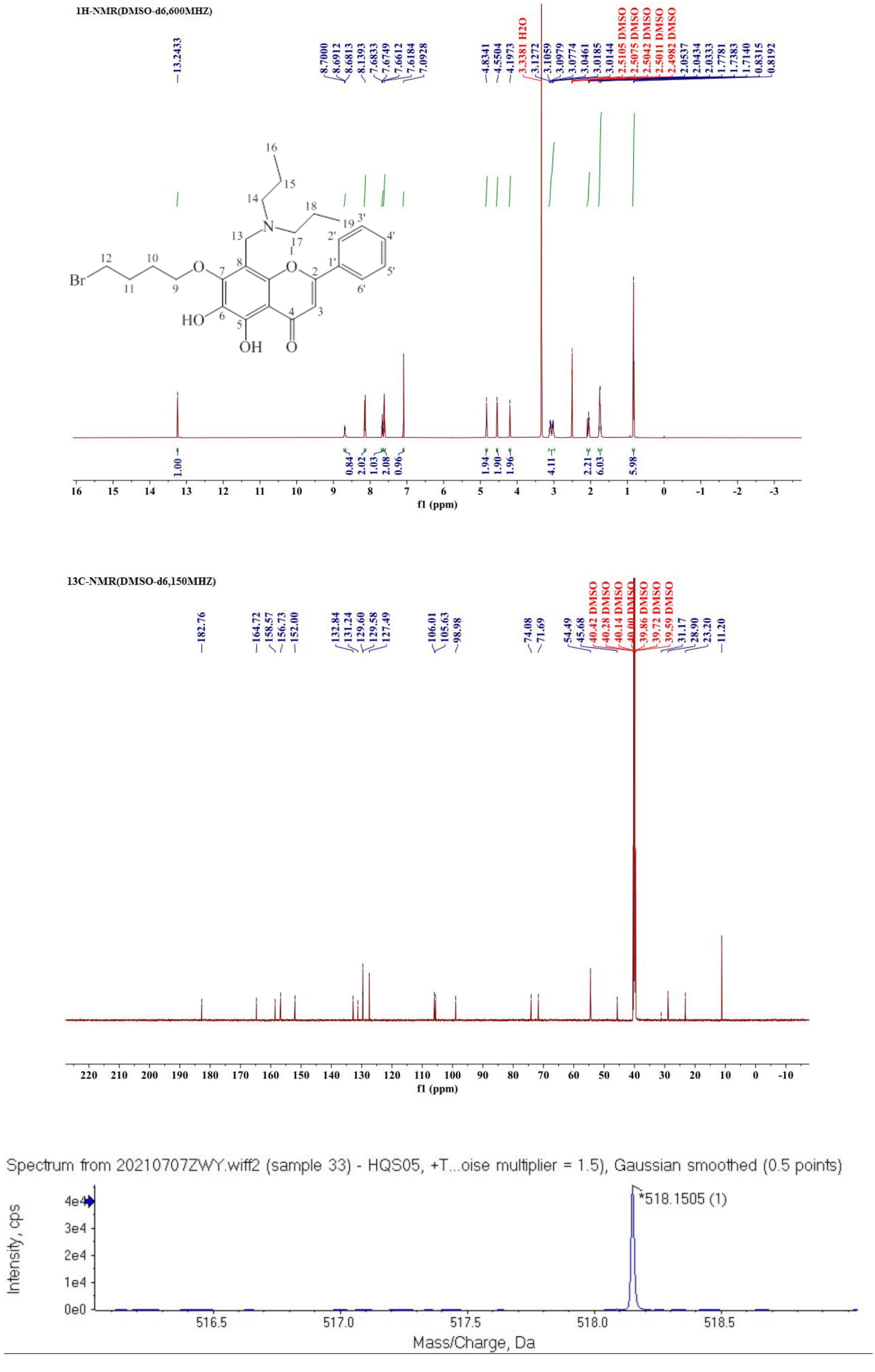

The ^{1}H-NMR, ^{13}C-NMR and HRMS spectra of compound **3b**

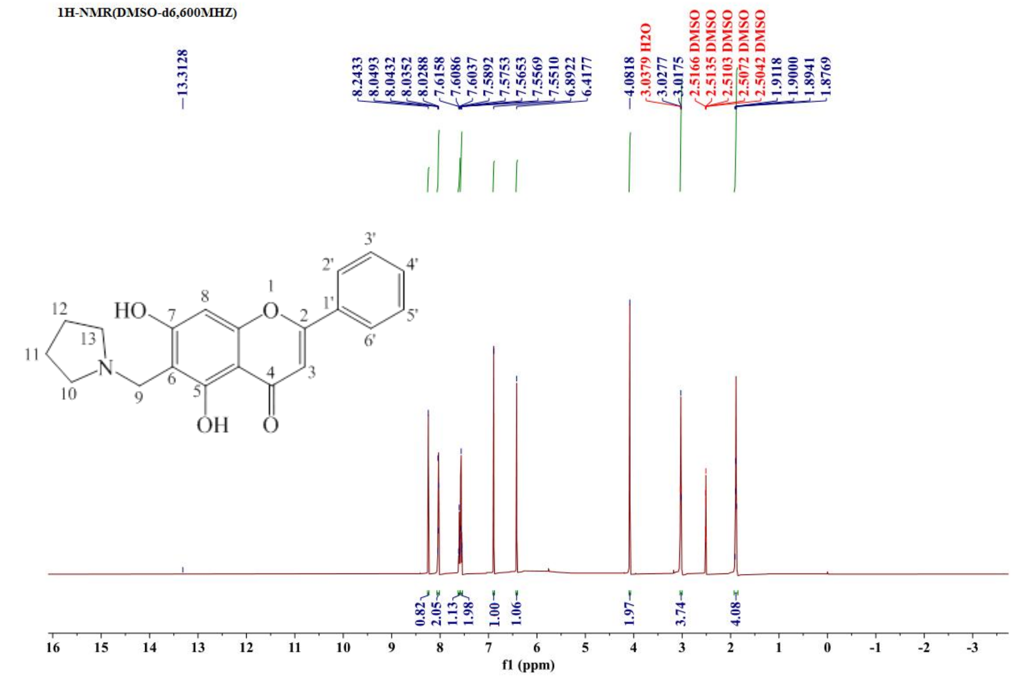

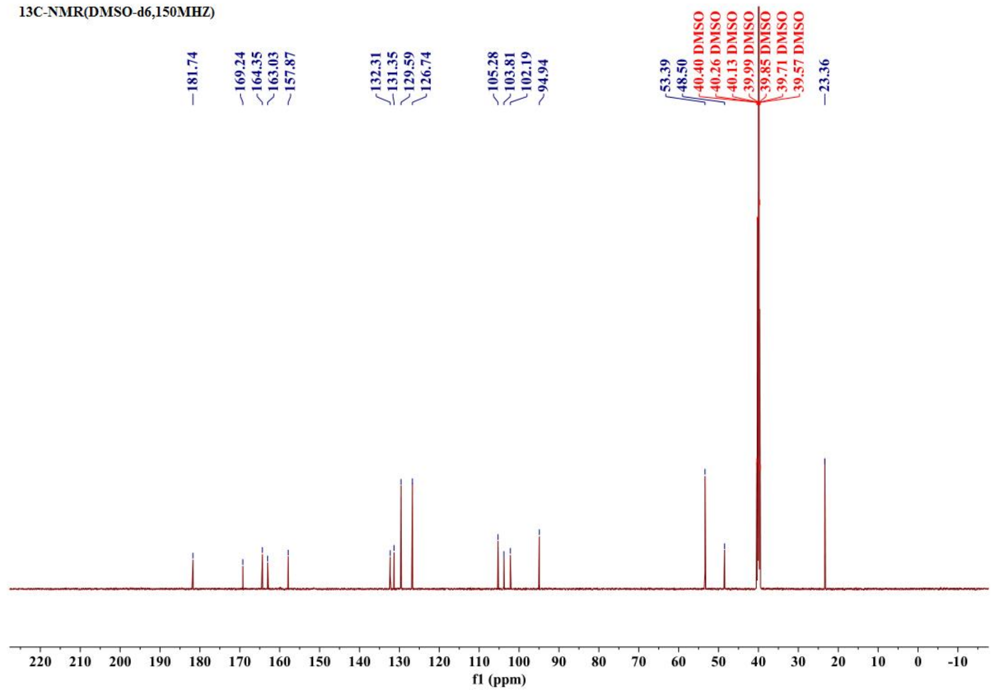

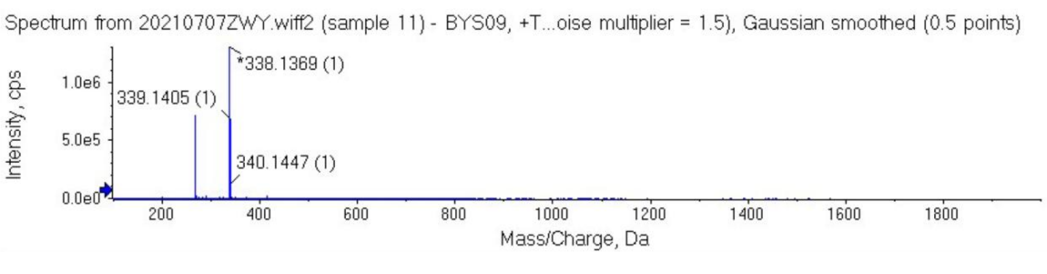

The ^{1}H-NMR, ^{13}C-NMR and HRMS spectra of compound **4a**

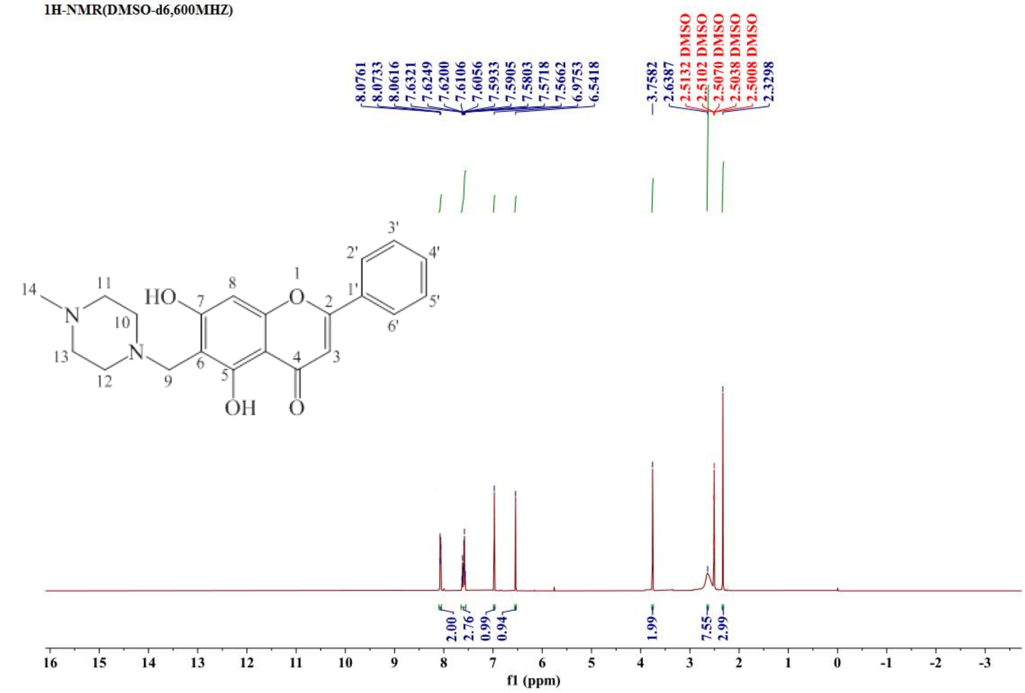

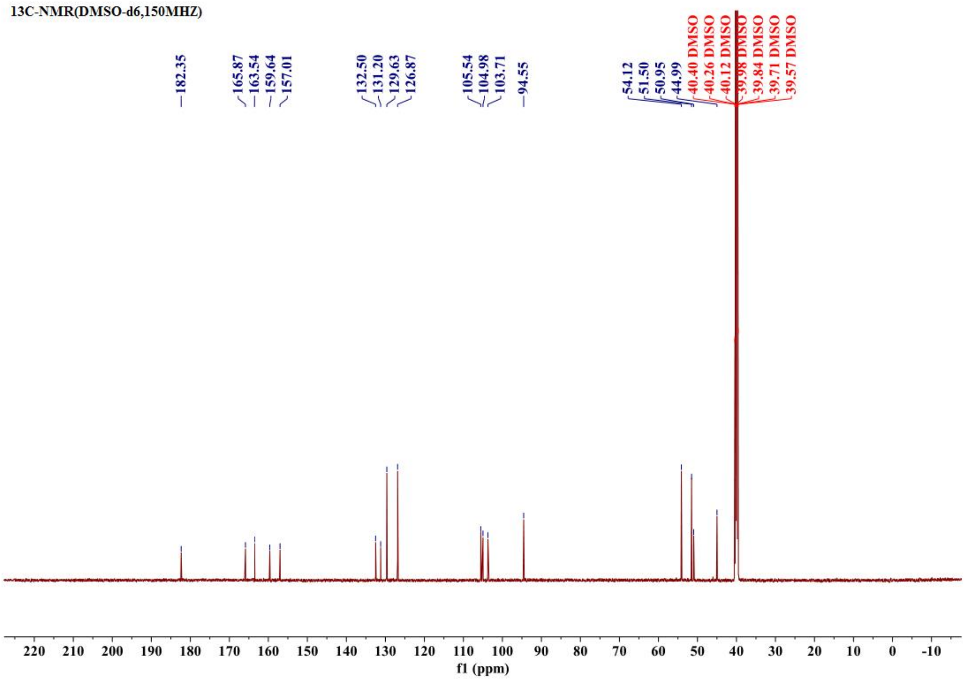

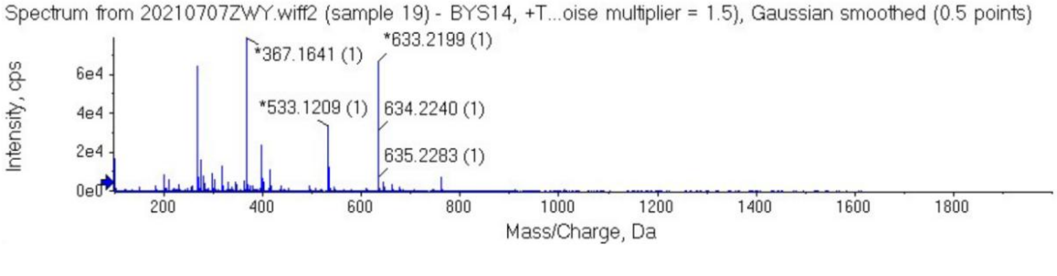

The ^{1}H-NMR, ^{13}C-NMR and HRMS spectra of compound **4b**

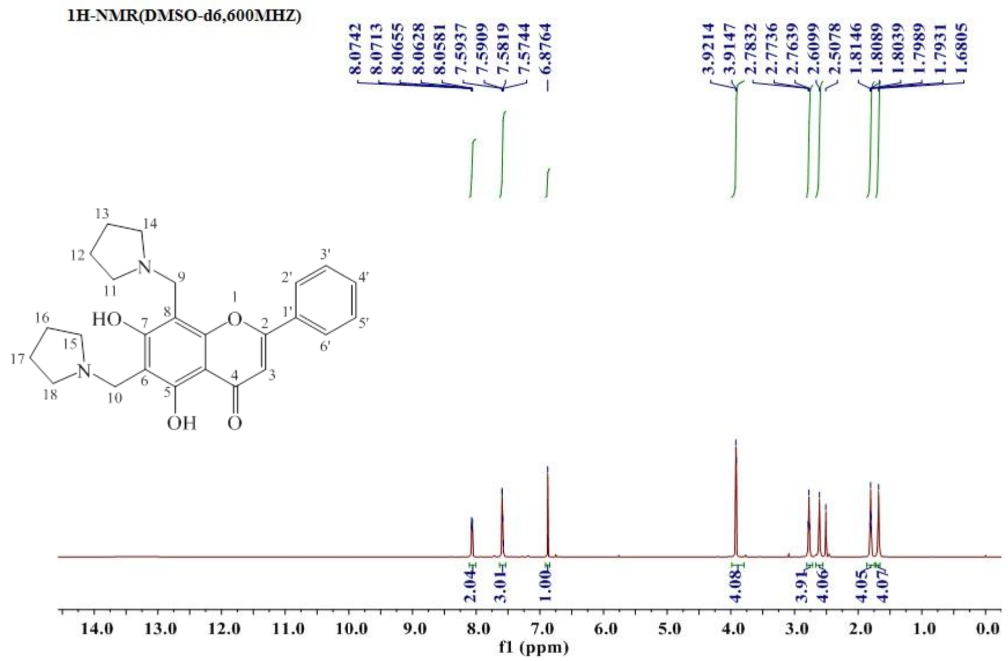

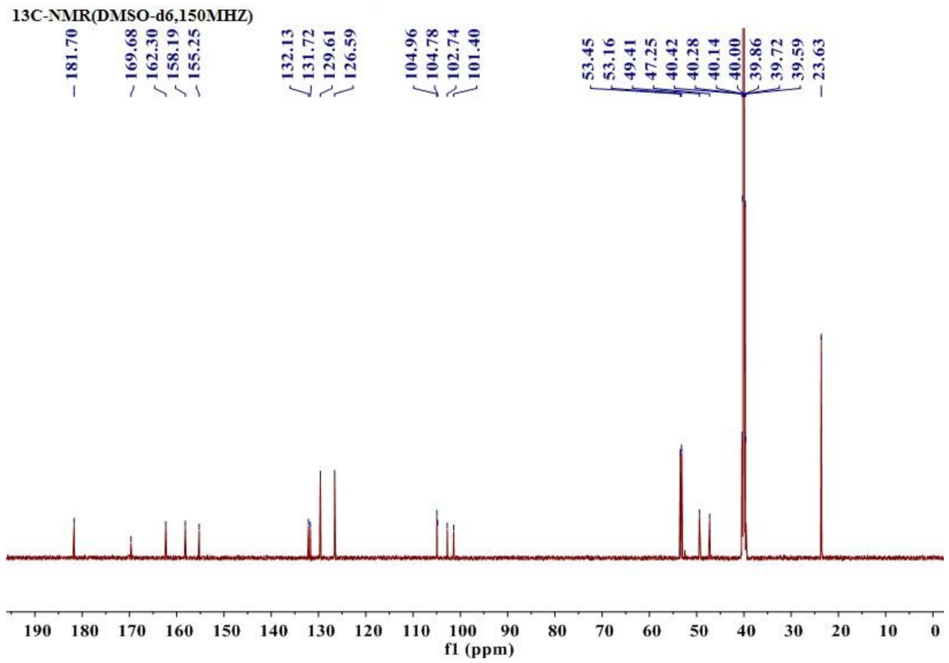

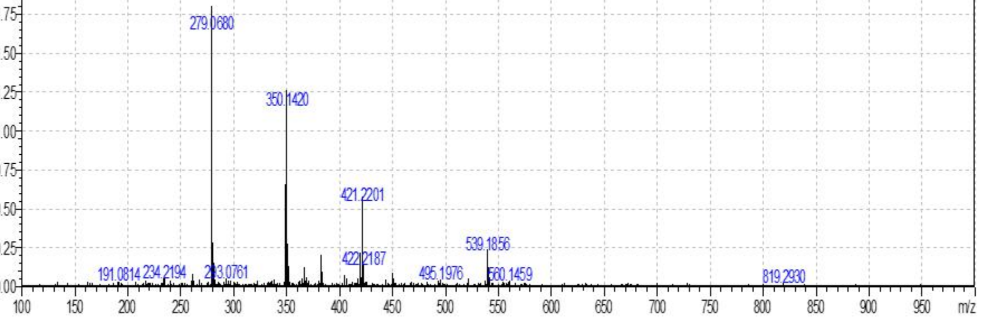

The ^{1}H-NMR, ^{13}C-NMR and HRMS spectra of compound **5a**

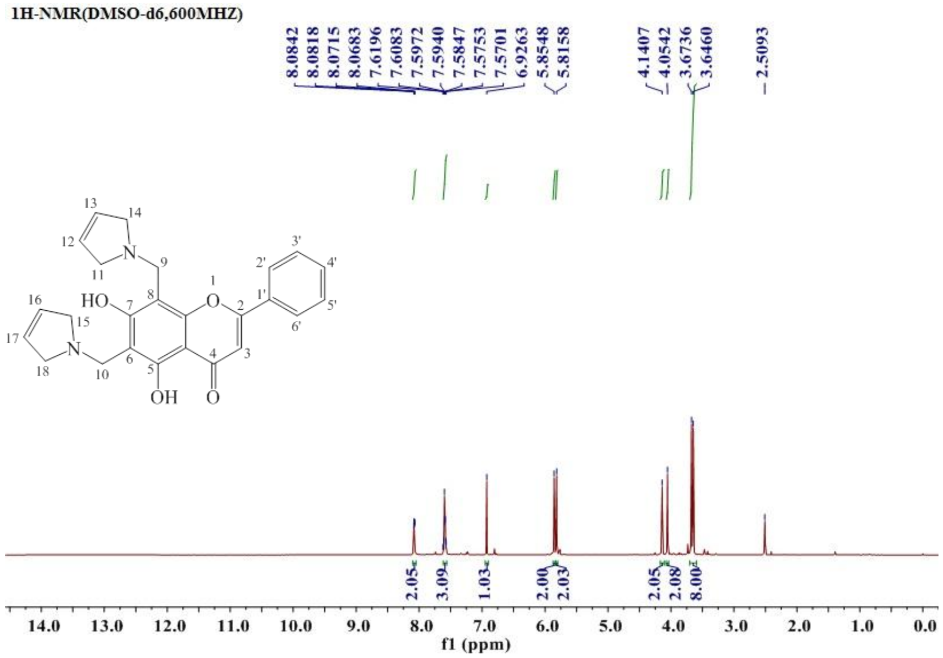

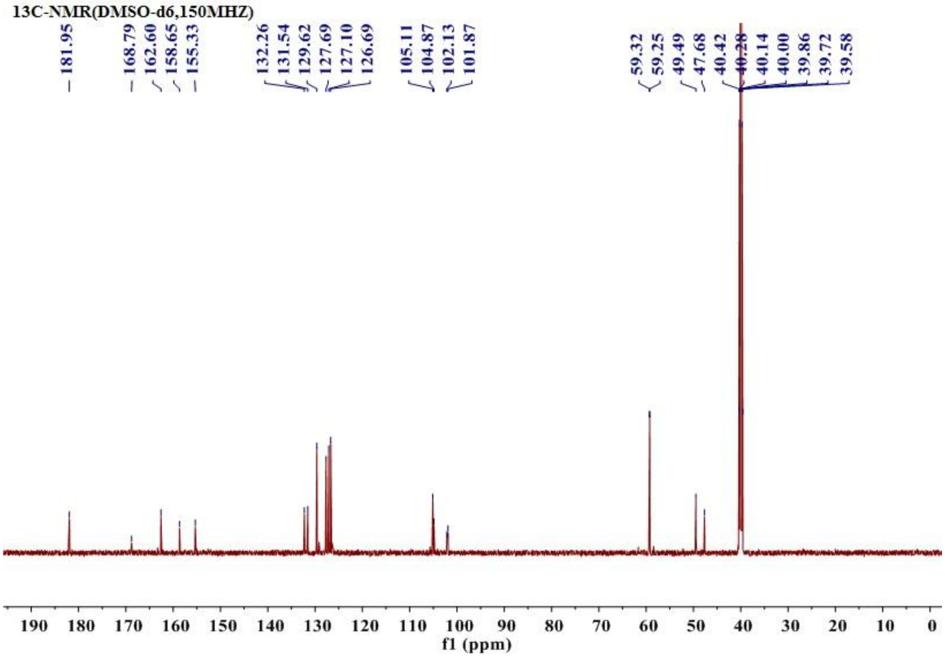

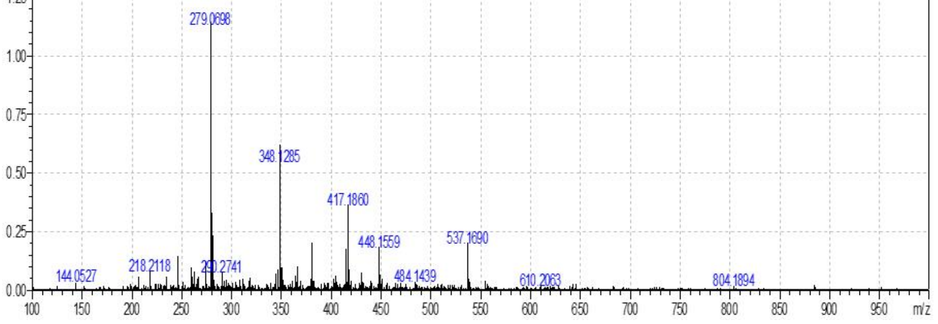

The ^{1}H-NMR, ^{13}C-NMR and HRMS spectra of compound **5b**

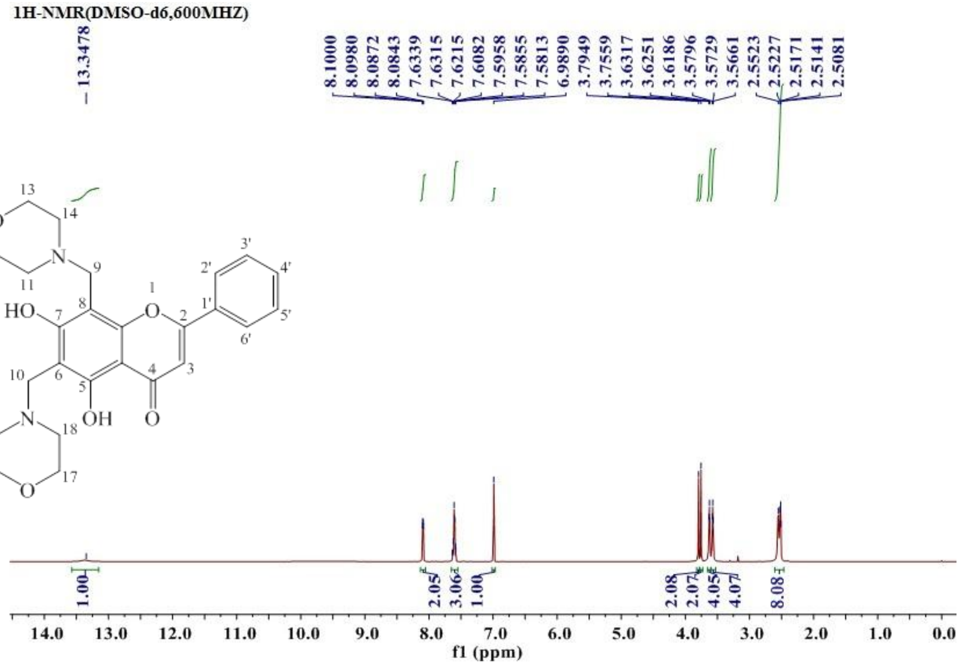

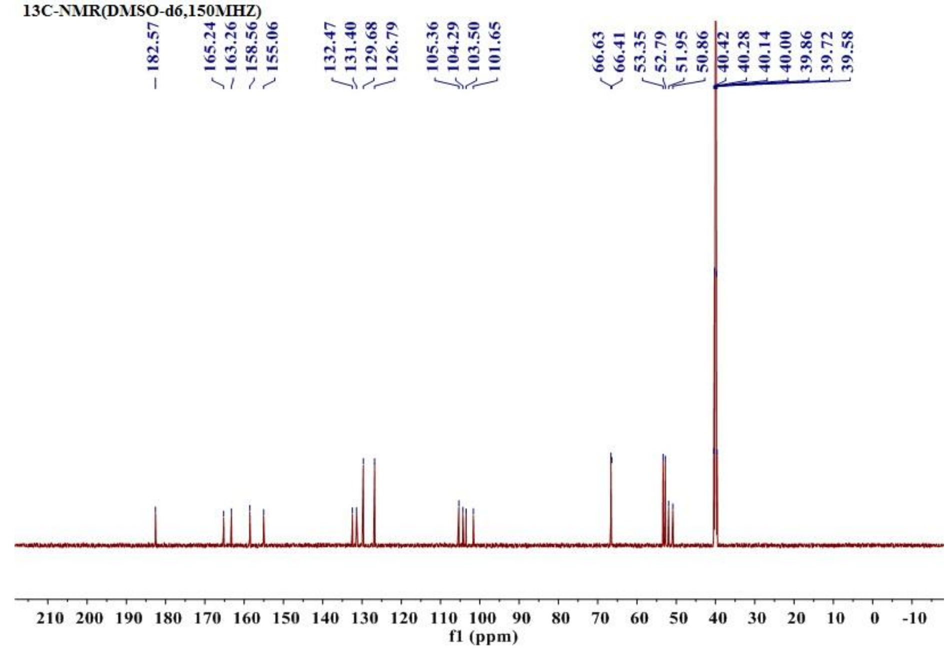

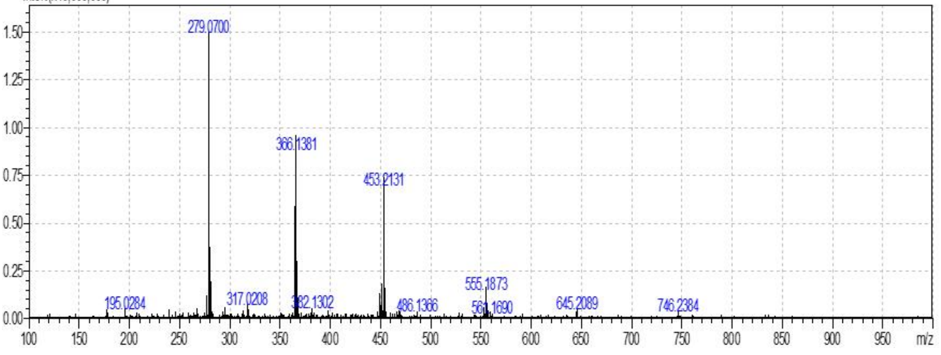

The ^{1}H-NMR, ^{13}C-NMR and HRMS spectra of compound **5c**

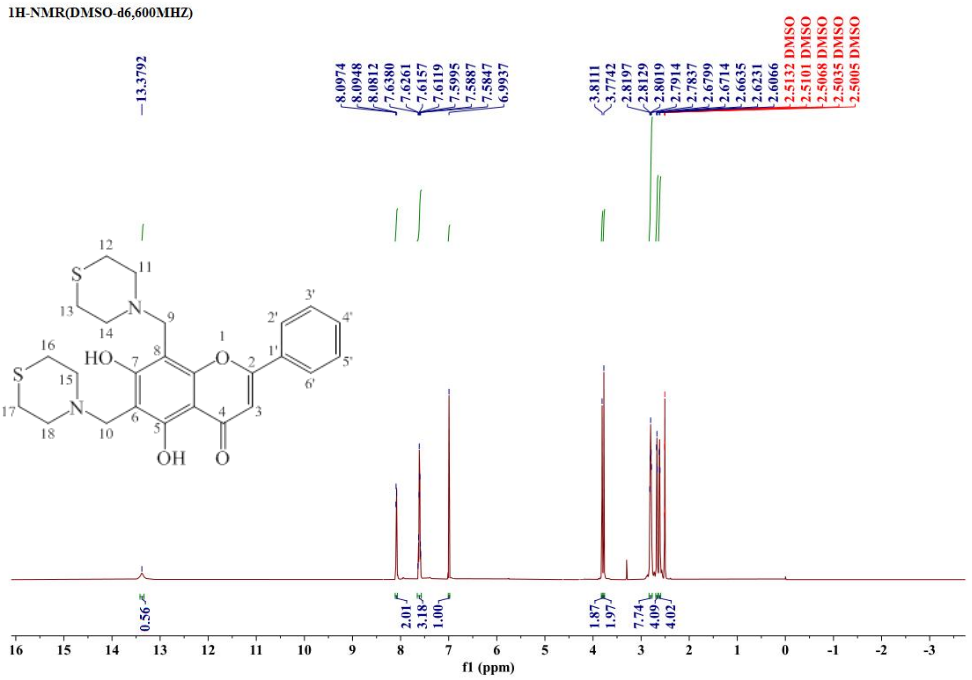

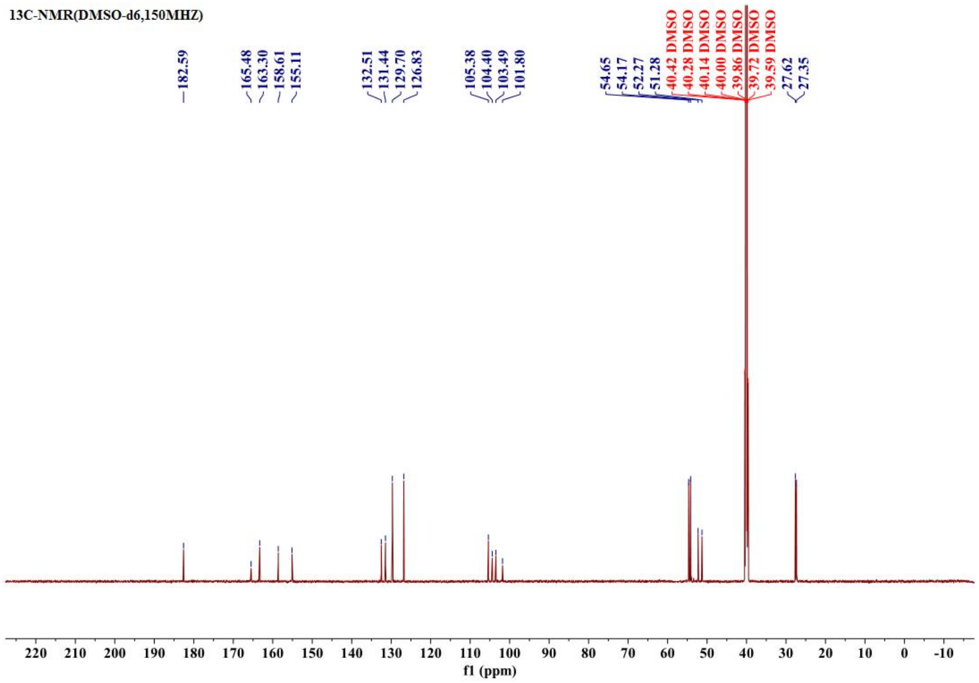

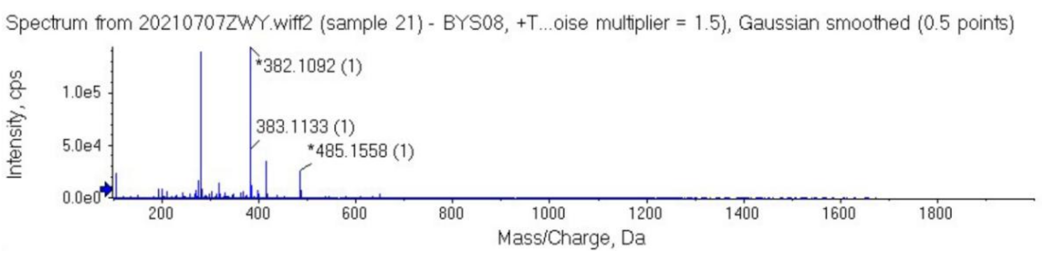

The ^{1}H-NMR, ^{13}C-NMR and HRMS spectra of compound **5d**

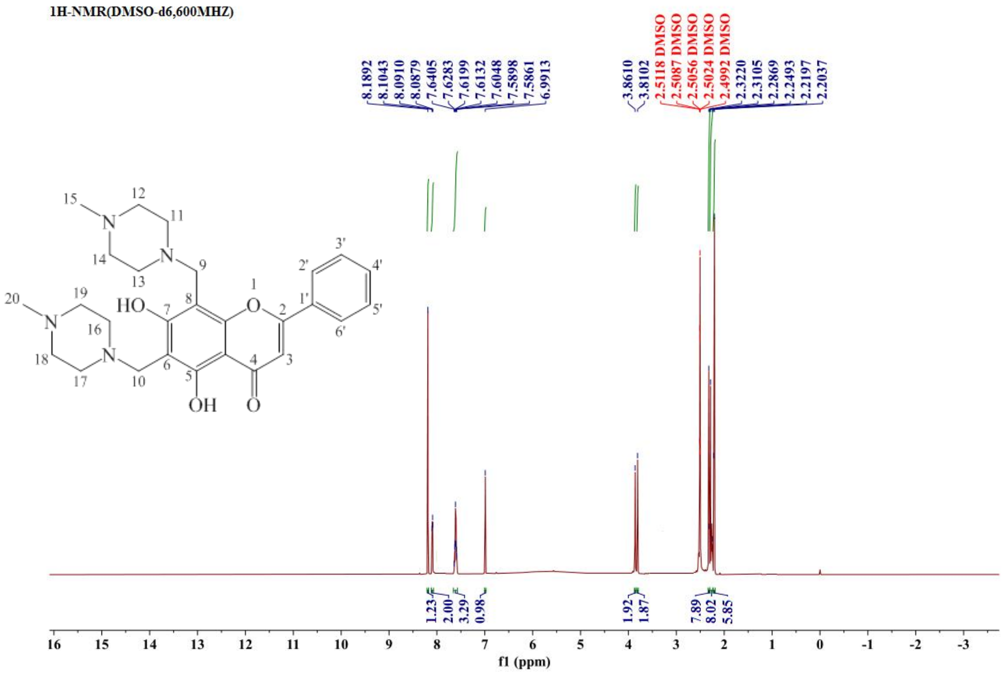

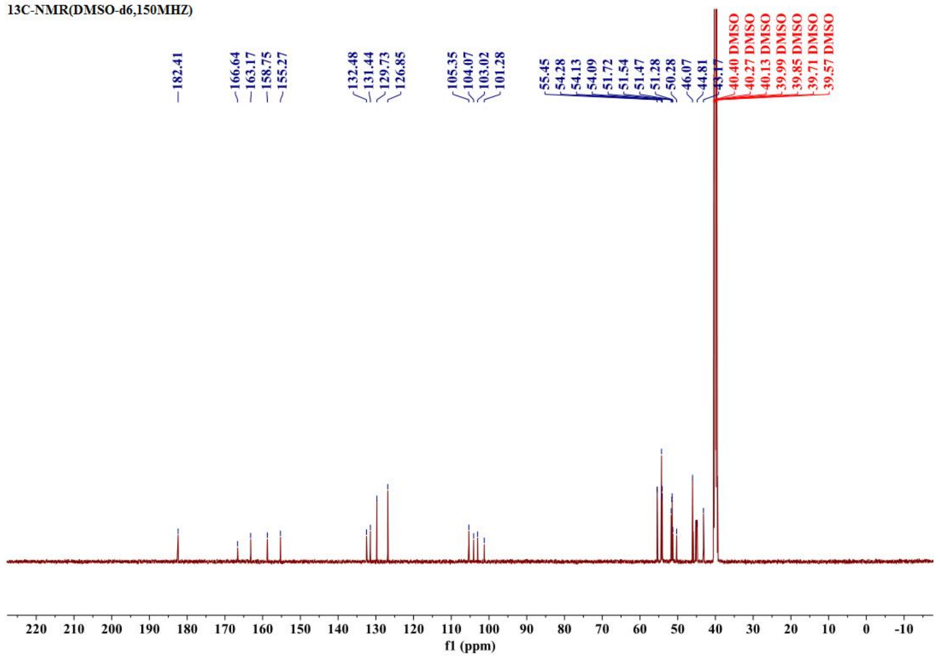

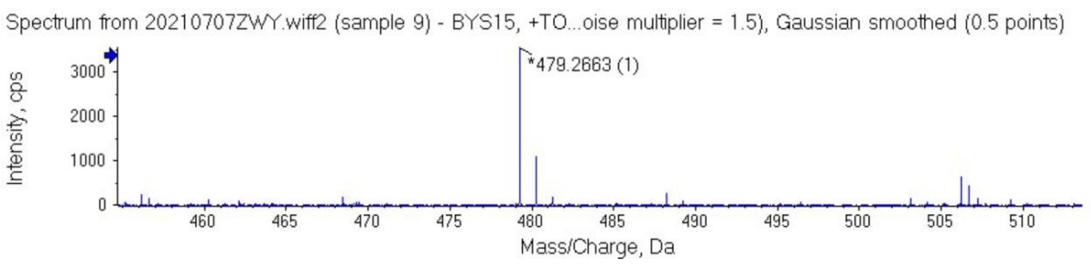

The ^{1}H-NMR, ^{13}C-NMR and HRMS spectra of compound **5e**

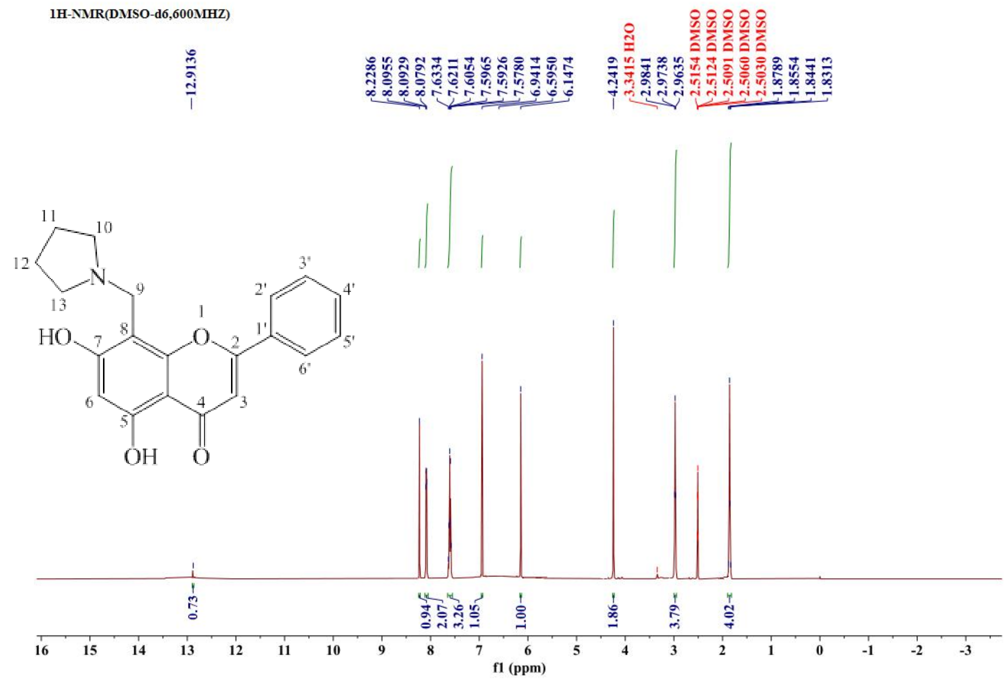

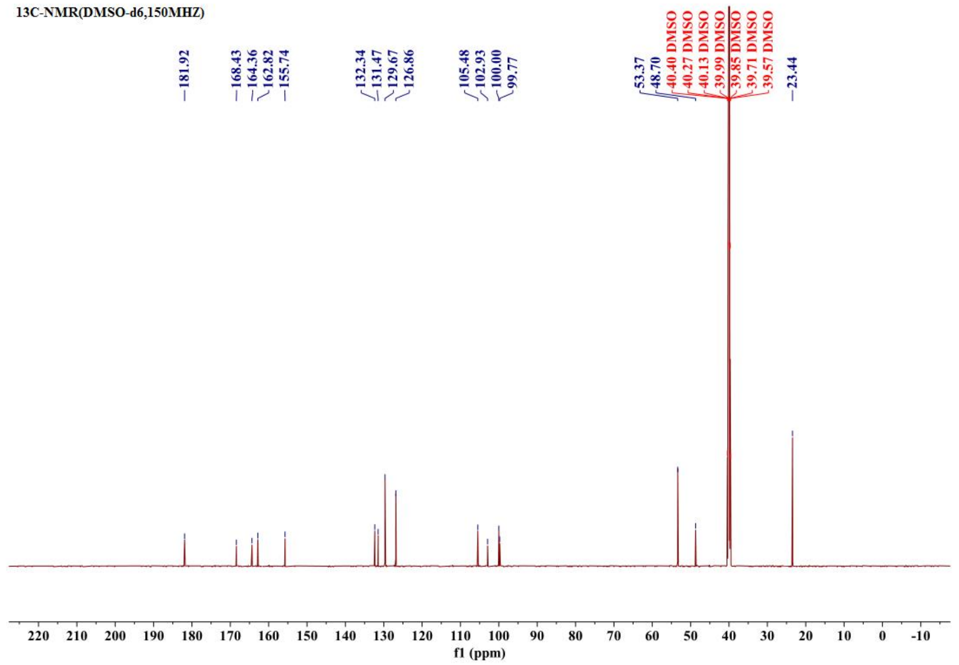

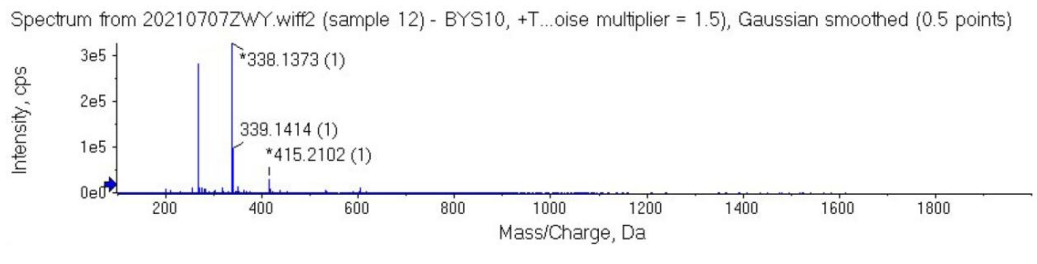

The ^{1}H-NMR, ^{13}C-NMR and HRMS spectra of compound **6a**

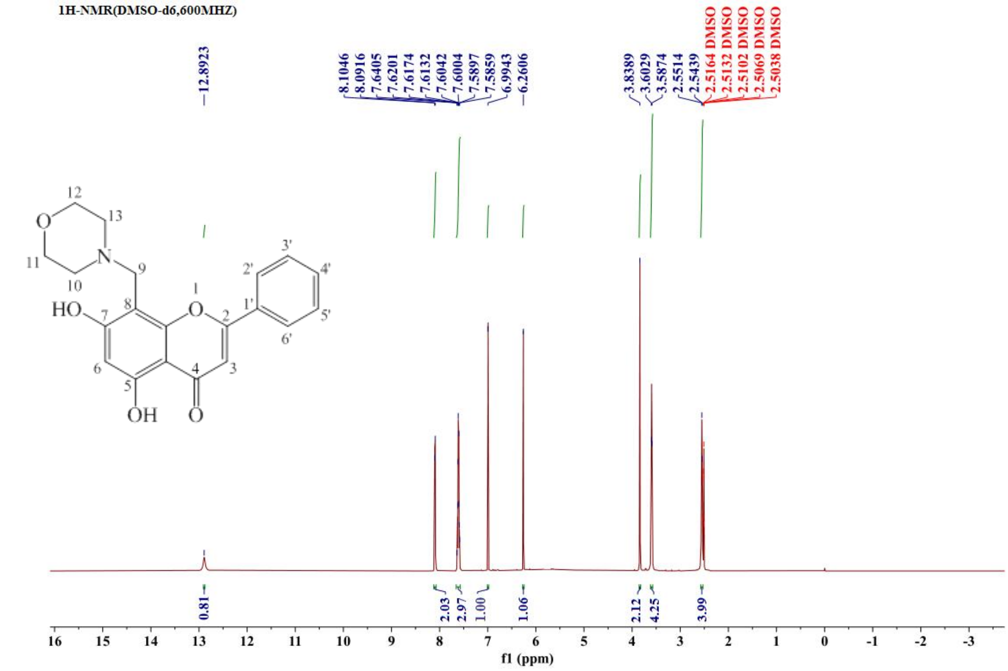

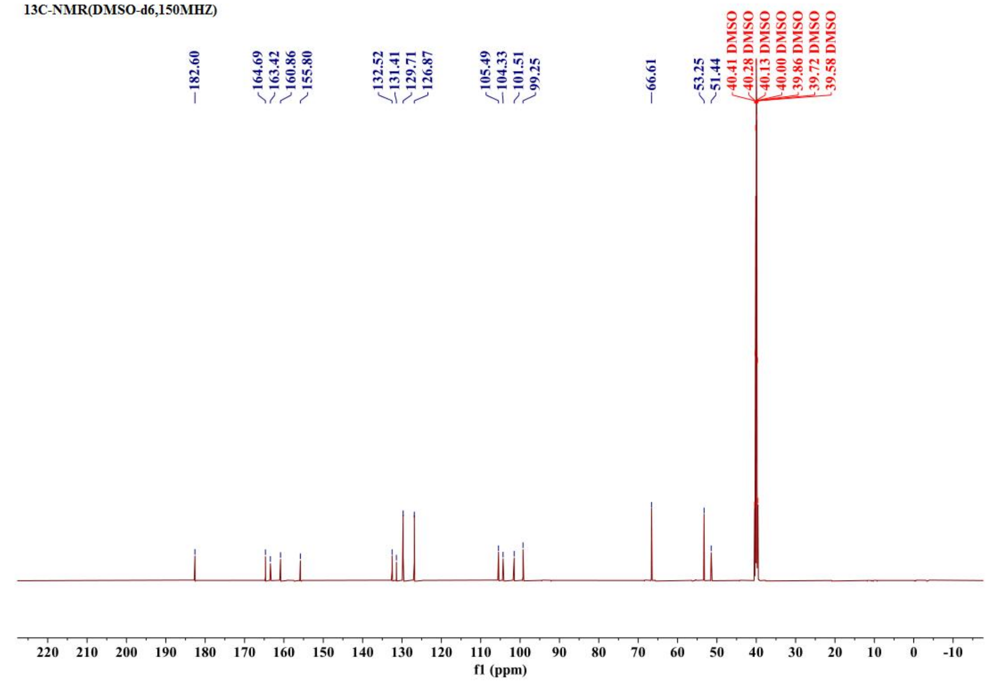

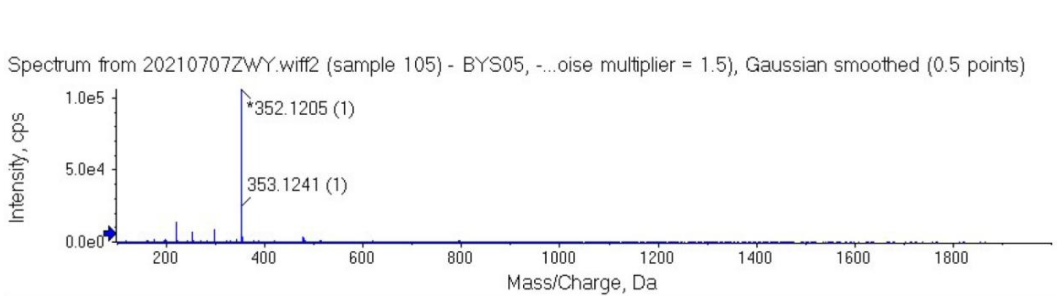

The ^{1}H-NMR, ^{13}C-NMR and HRMS spectra of compound **6b**

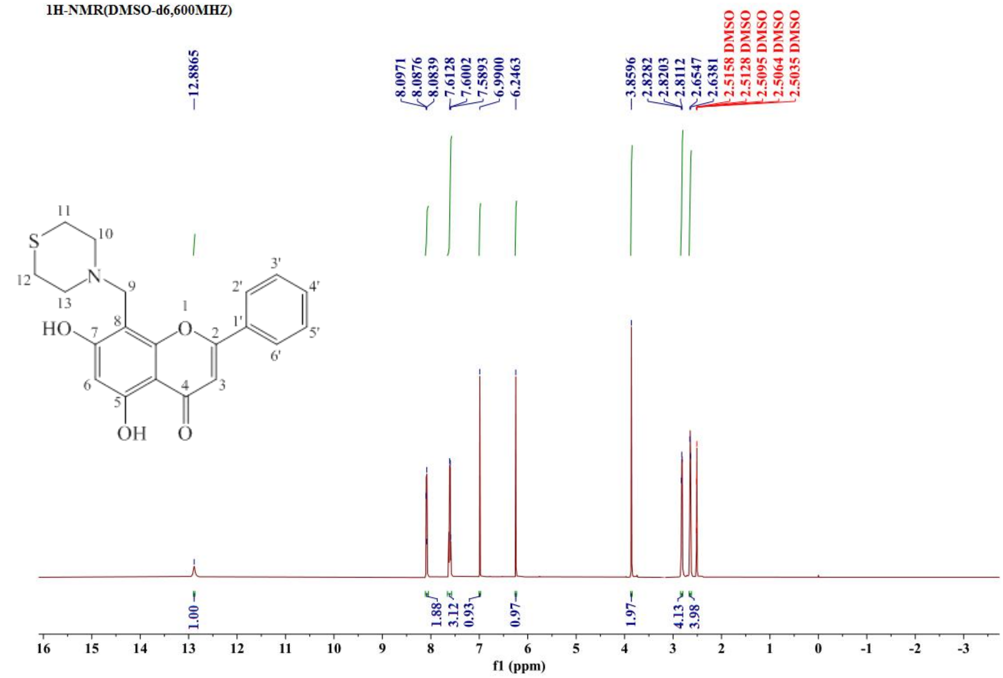

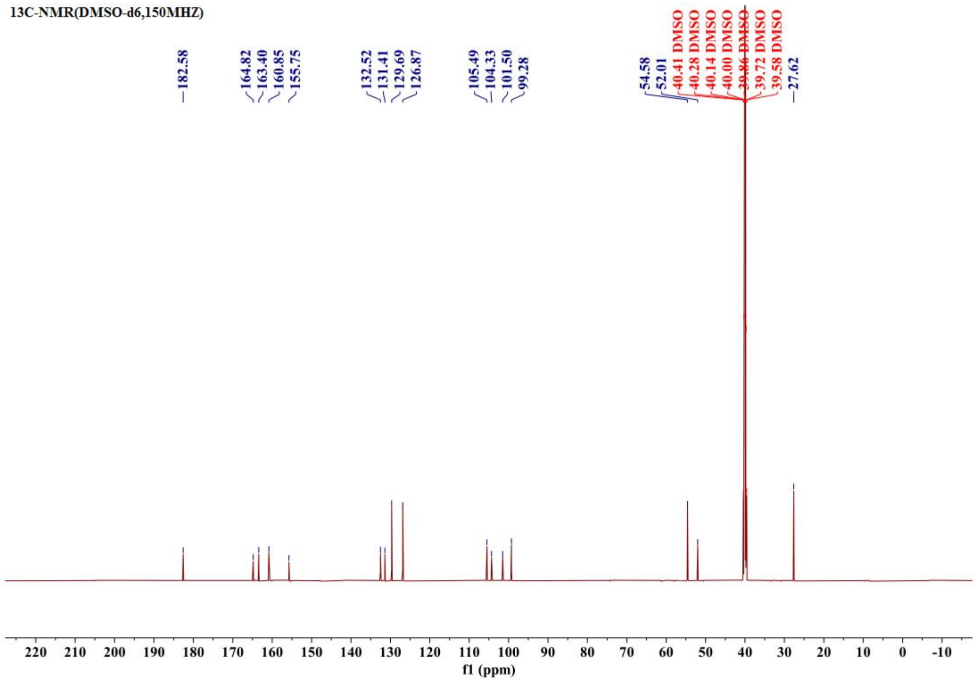

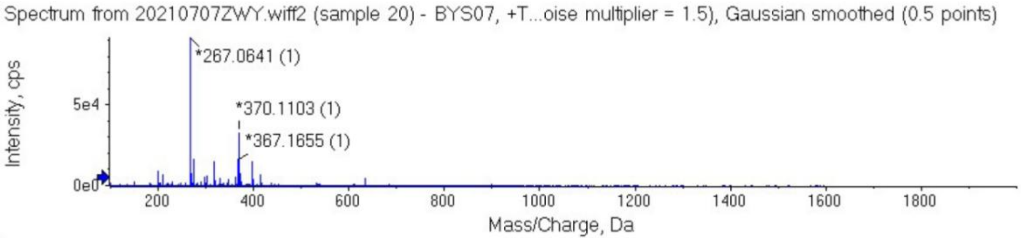

The ^{1}H-NMR, ^{13}C-NMR and HRMS spectra of compound **6c**

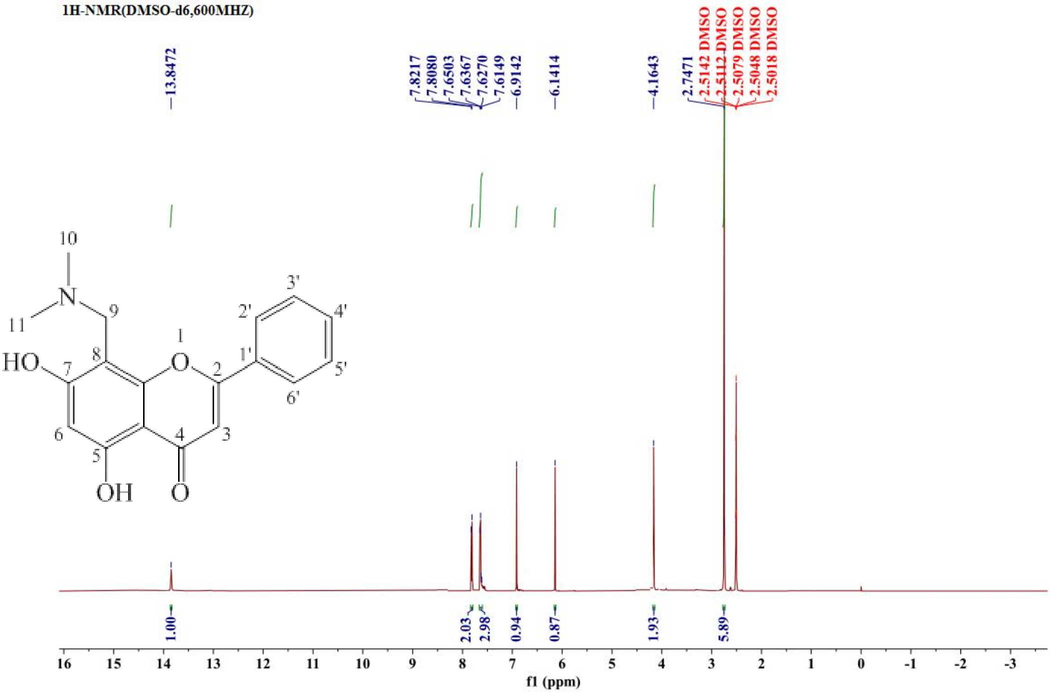

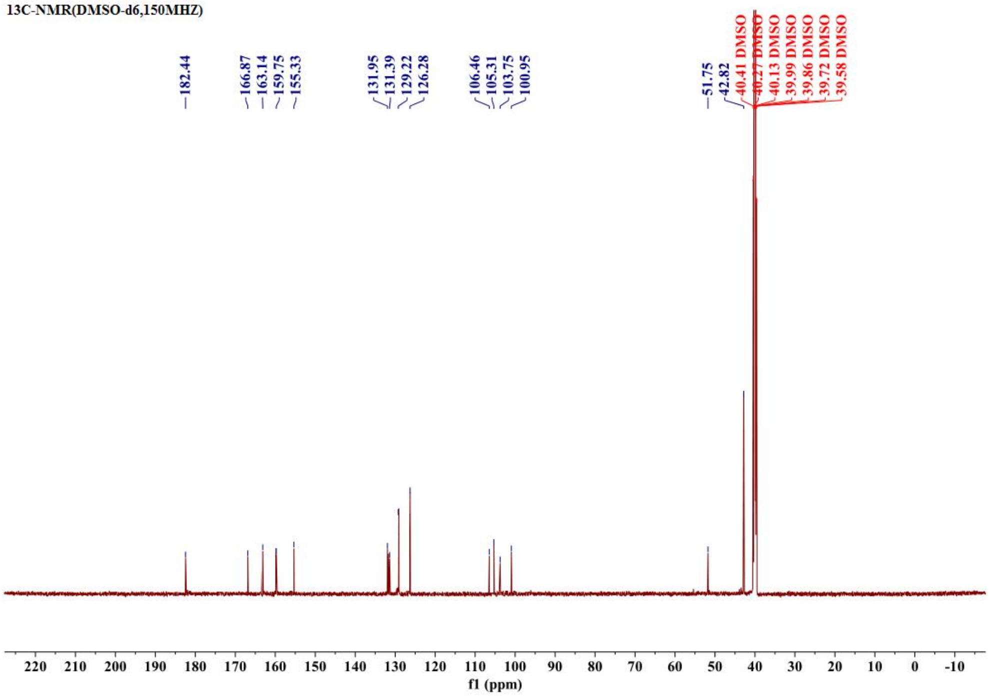

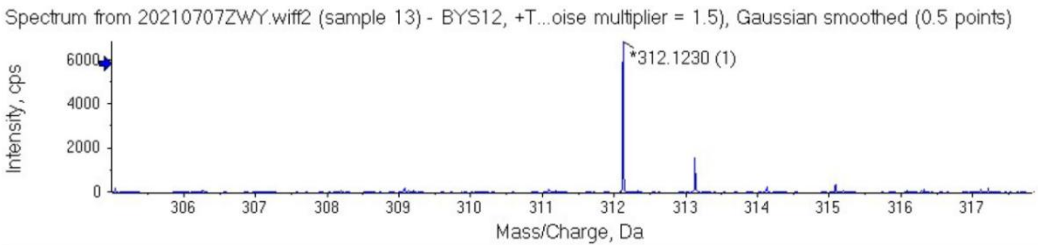

The ^{1}H-NMR, ^{13}C-NMR and HRMS spectra of compound **6d**

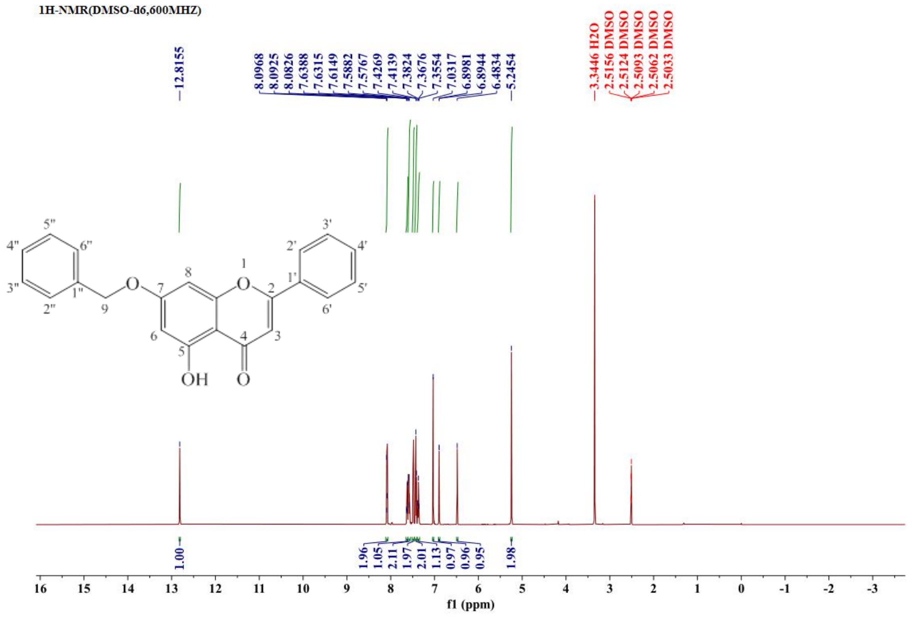

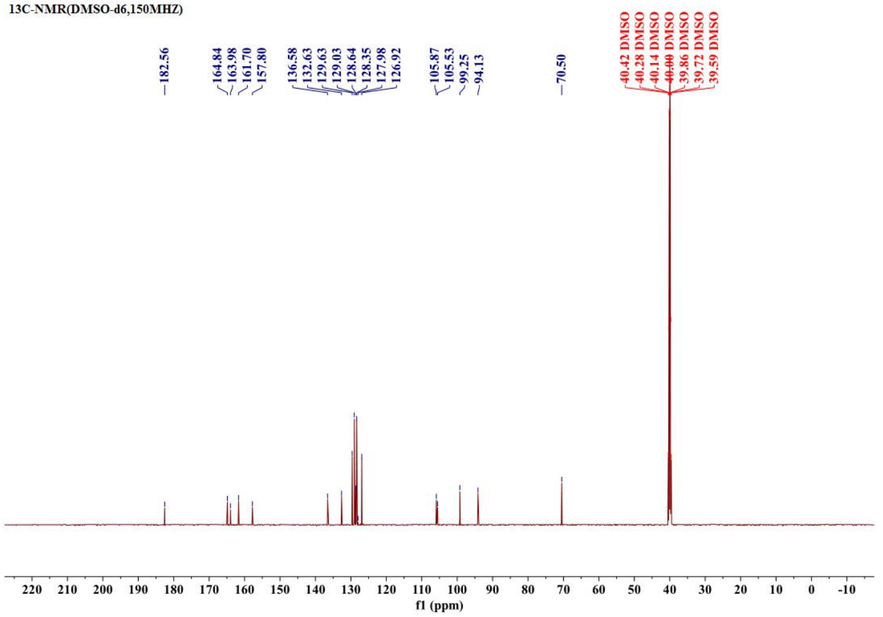

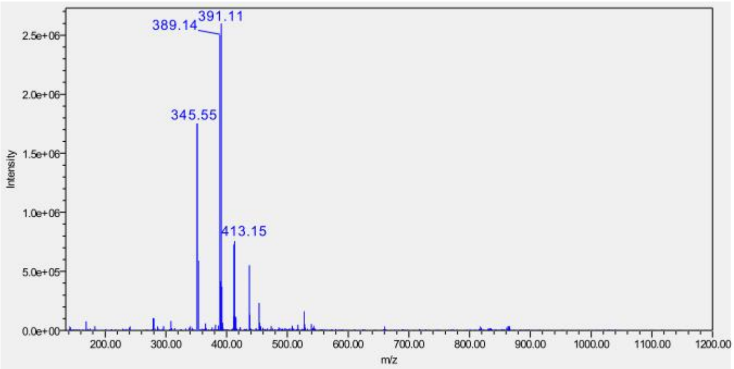

The ^{1}H-NMR, ^{13}C-NMR and MS spectra of compound **7a**

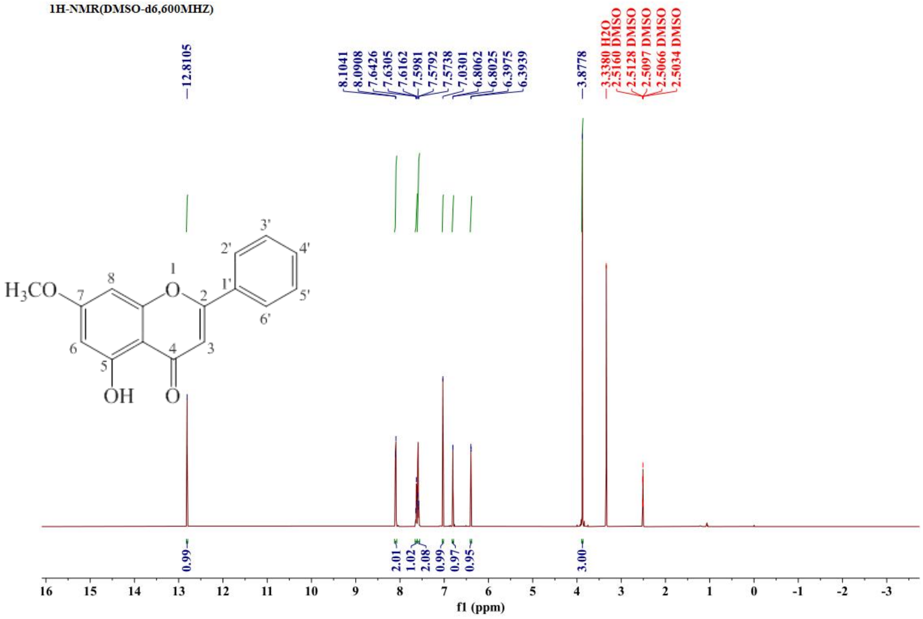

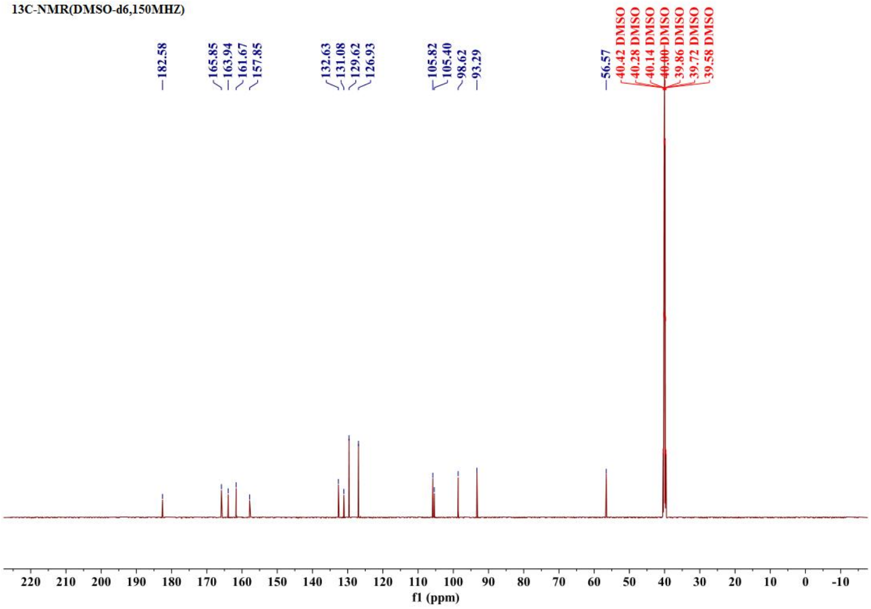

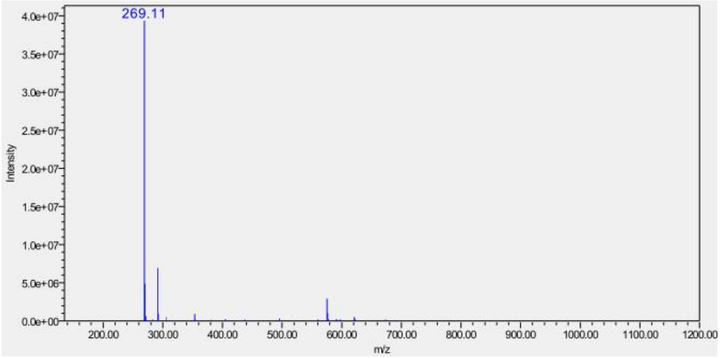

The ^{1}H-NMR, ^{13}C-NMR and MS spectra of compound **7b**

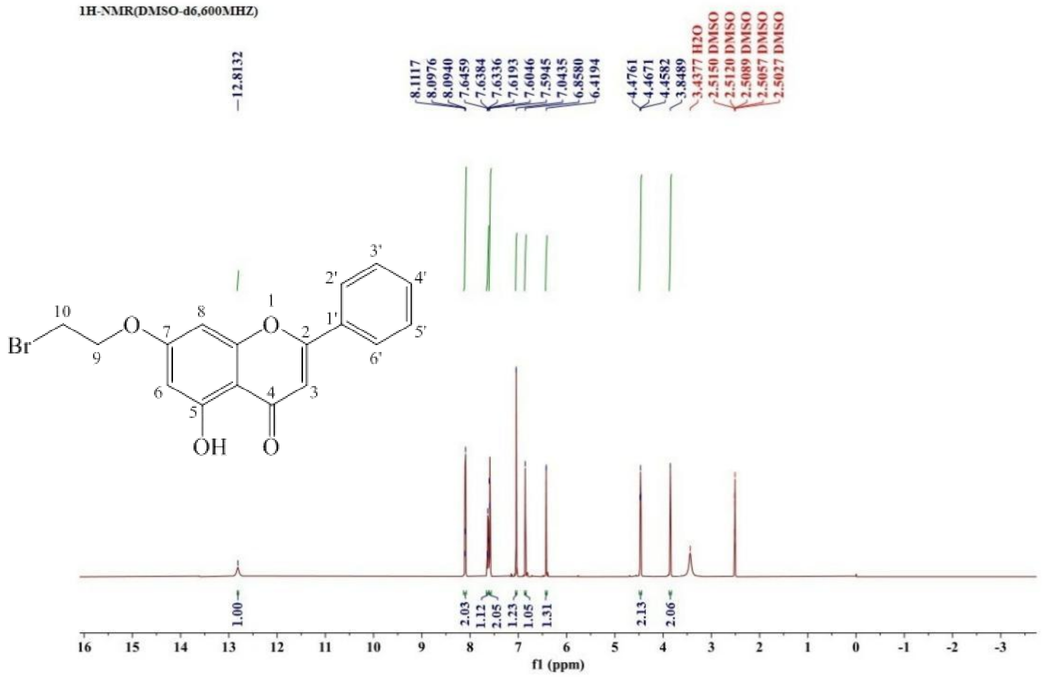

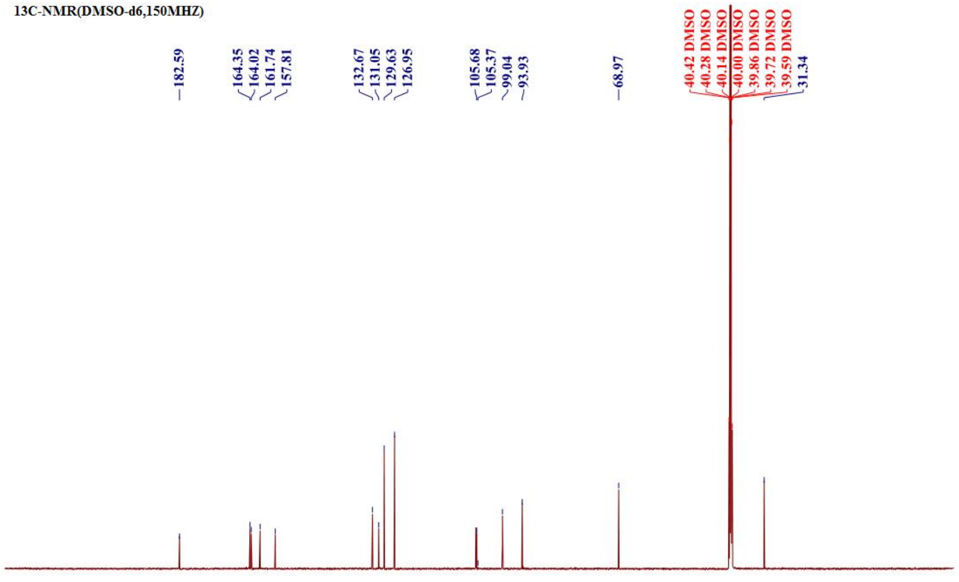

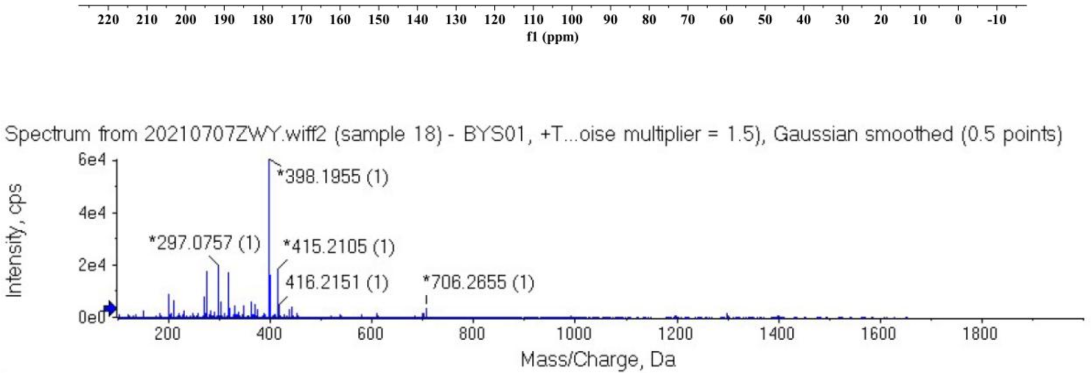

The ^{1}H-NMR, ^{13}C-NMR and HRMS spectra of compound **7c**

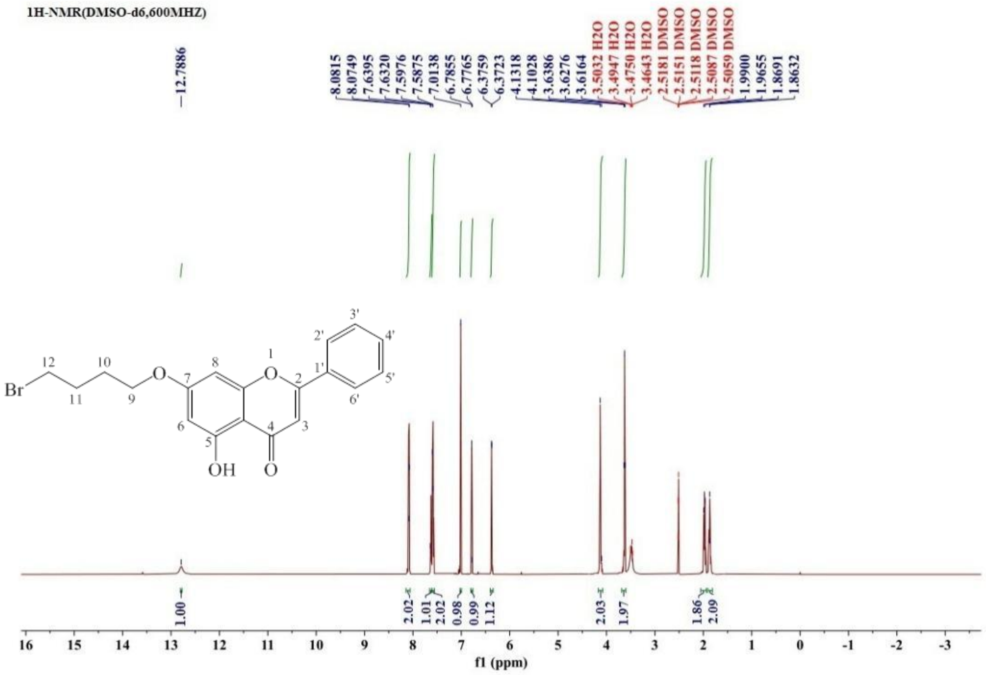

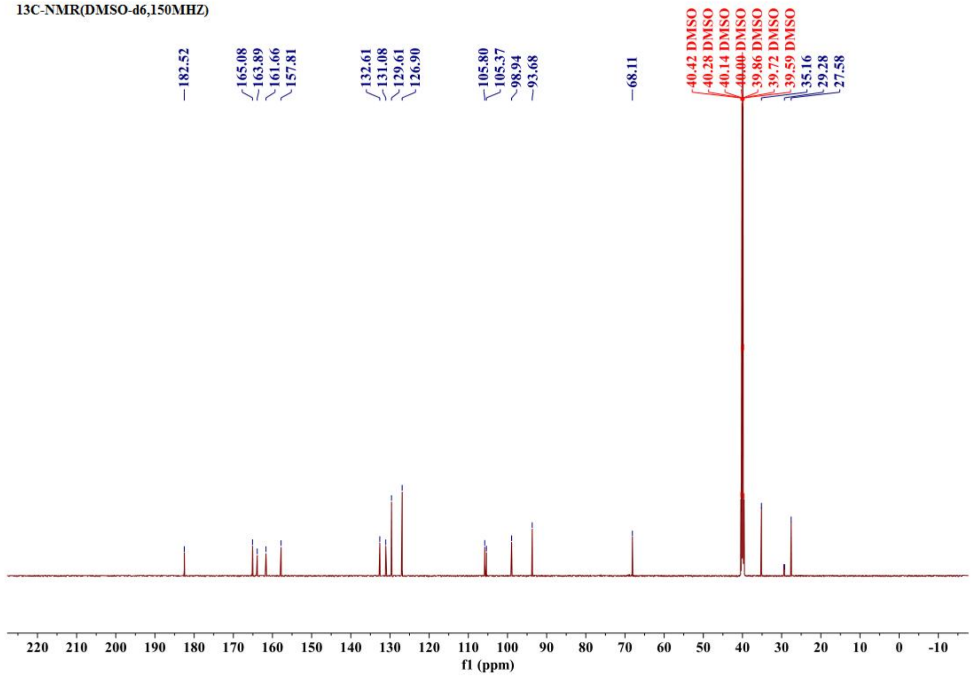

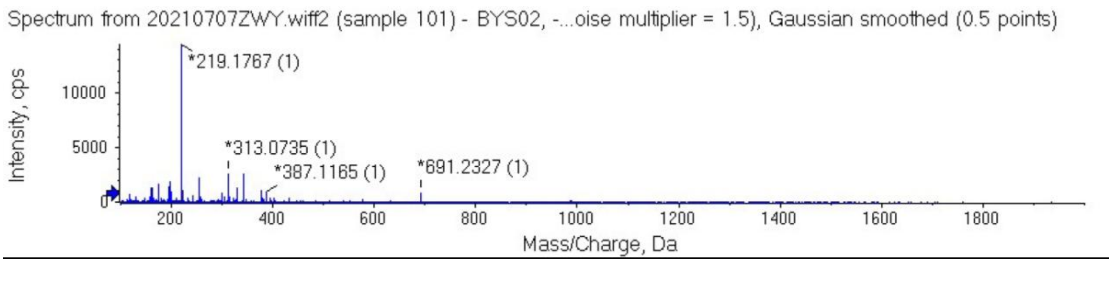

The ^1H-NMR, ^{13}C-NMR and HRMS spectra of compound **7d**

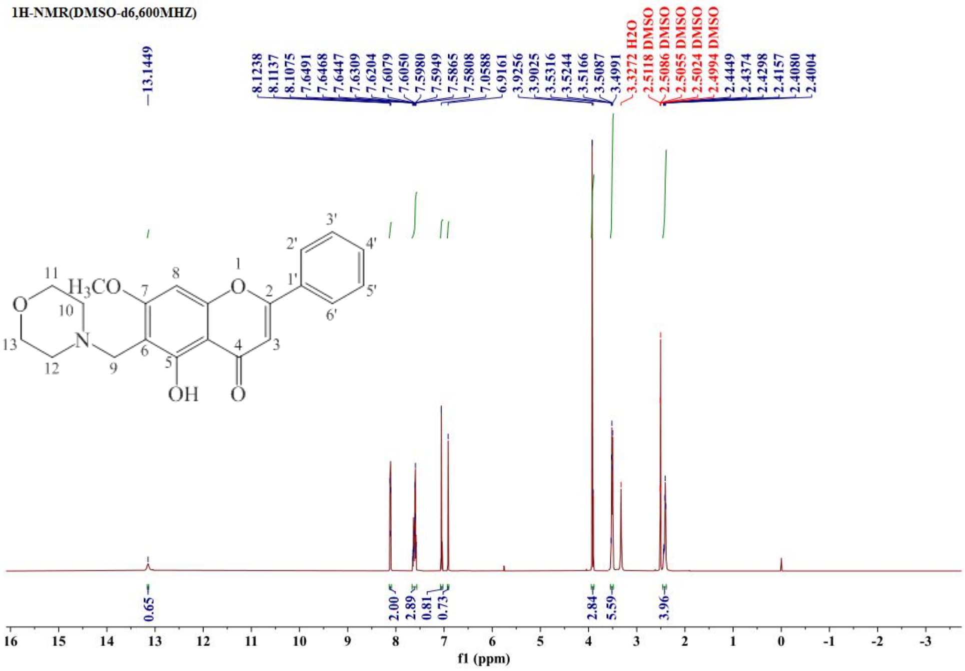

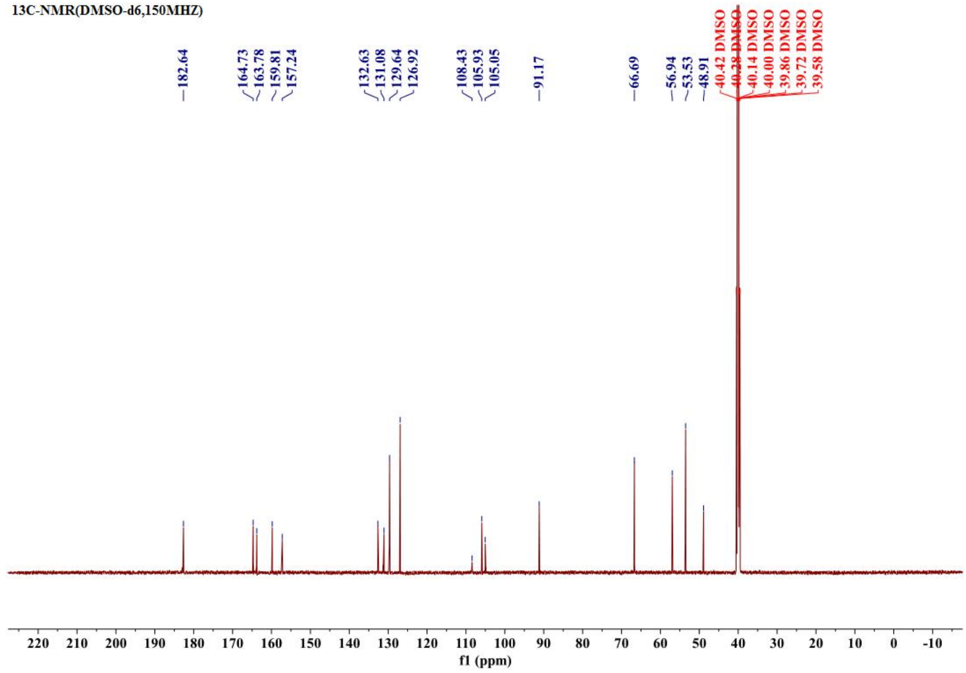

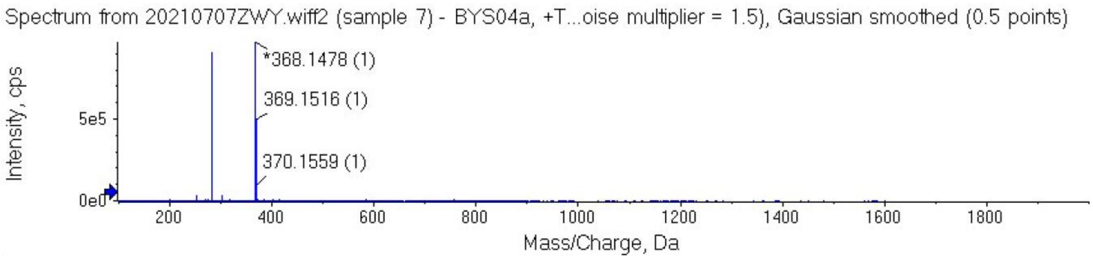

The ^{1}H-NMR, ^{13}C-NMR and HRMS spectra of compound **8a**

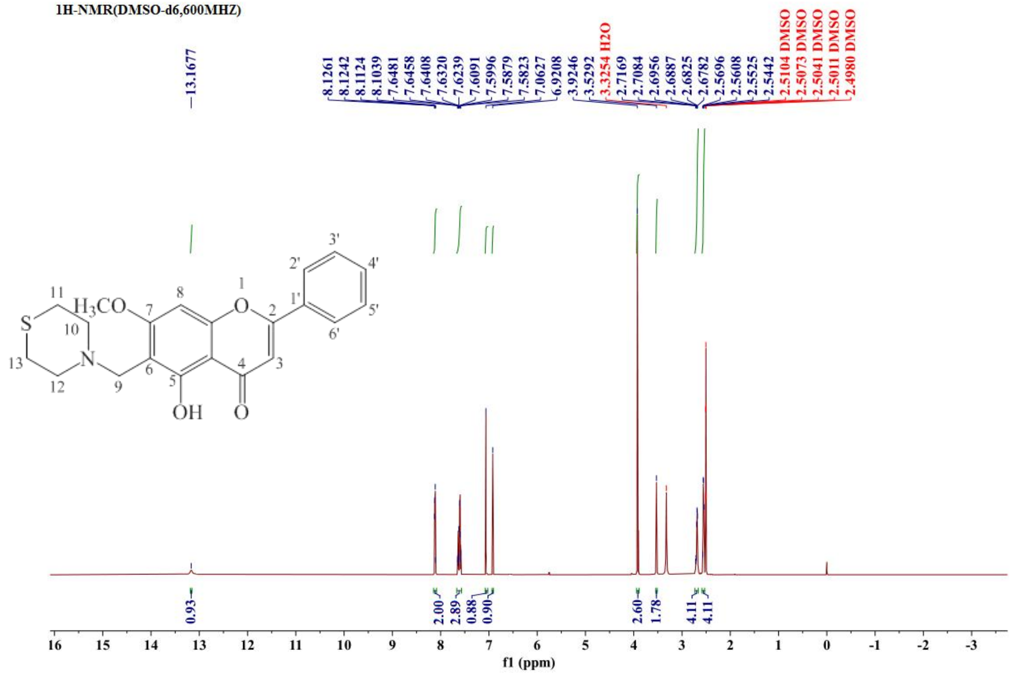

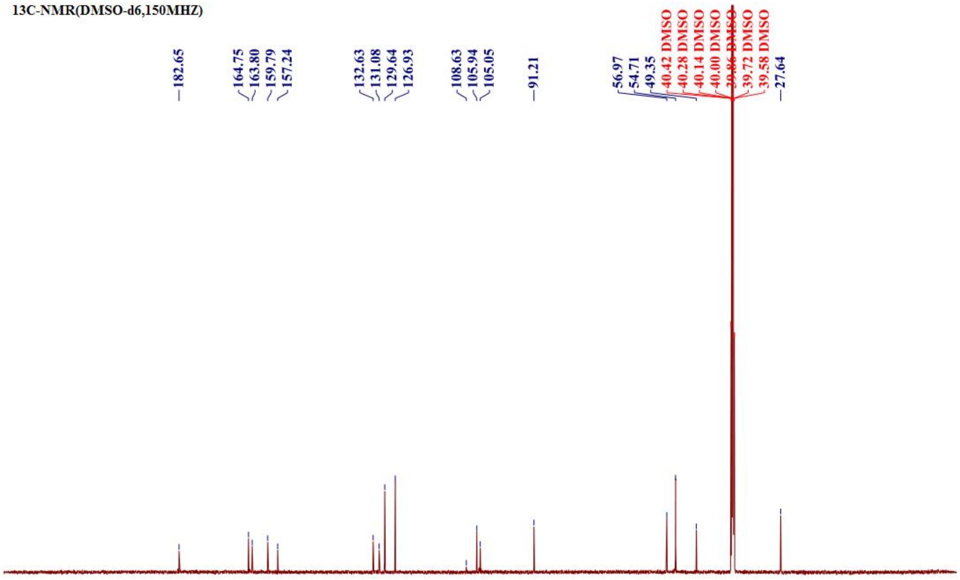

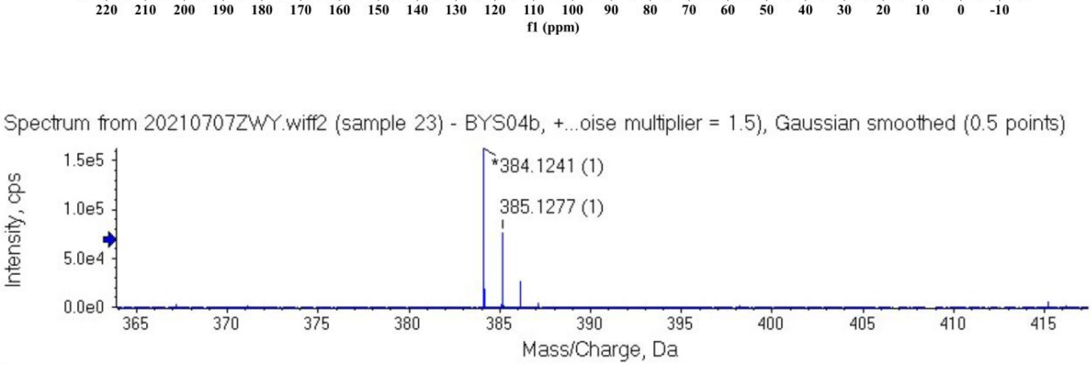

The ^{1}H-NMR, ^{13}C-NMR and HRMS spectra of compound **8b**

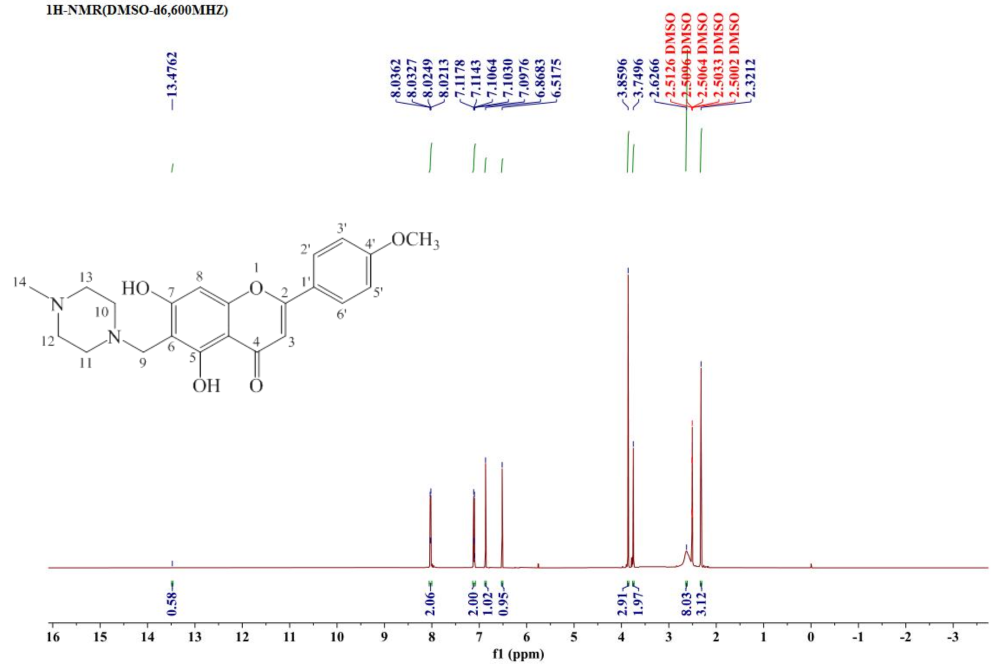

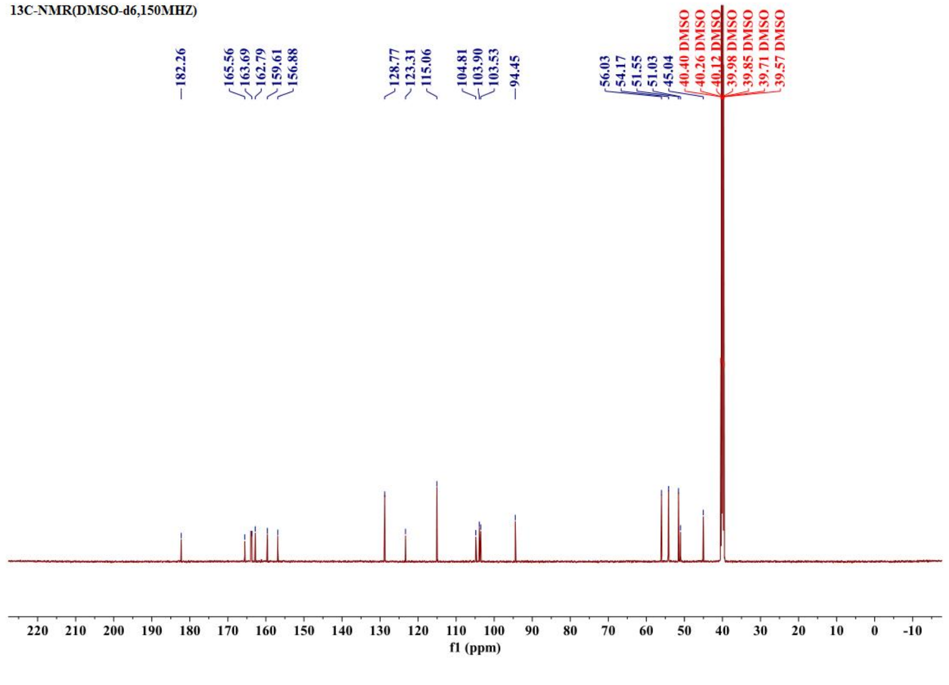

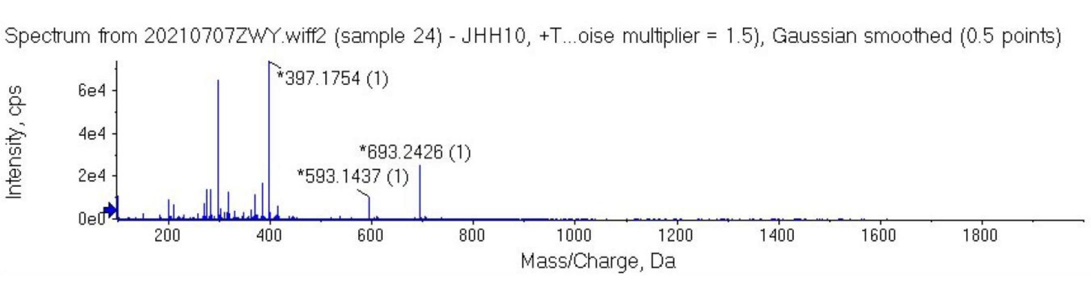

The ^{1}H-NMR, ^{13}C-NMR and HRMS spectra of compound **9a**

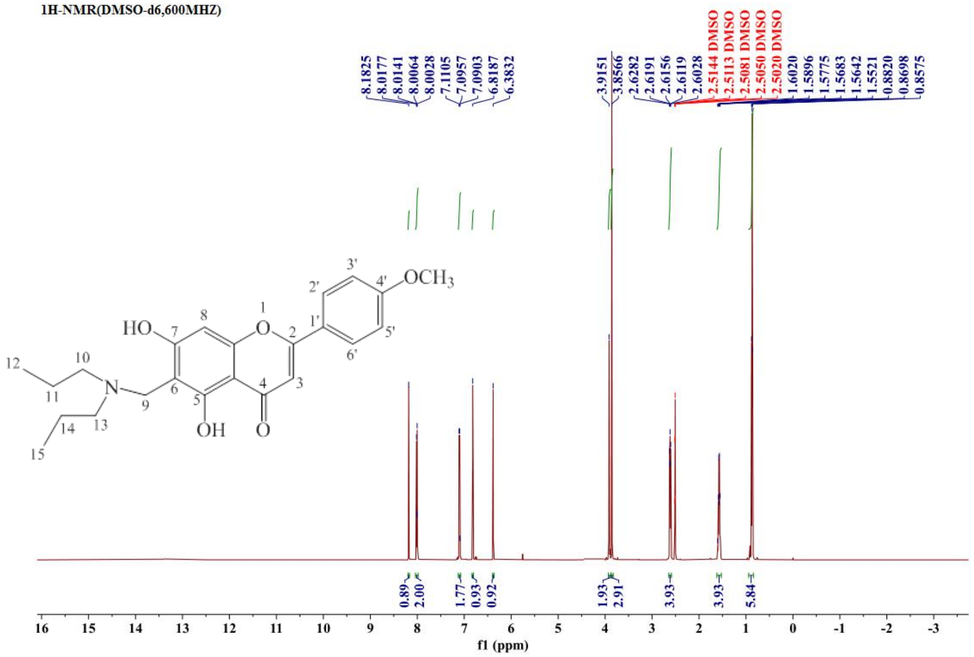

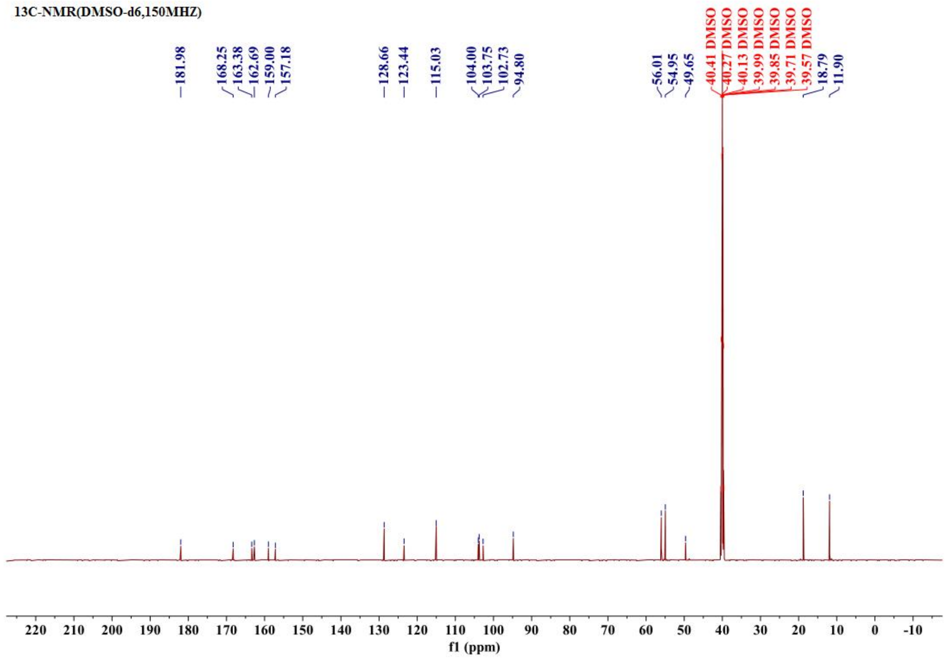

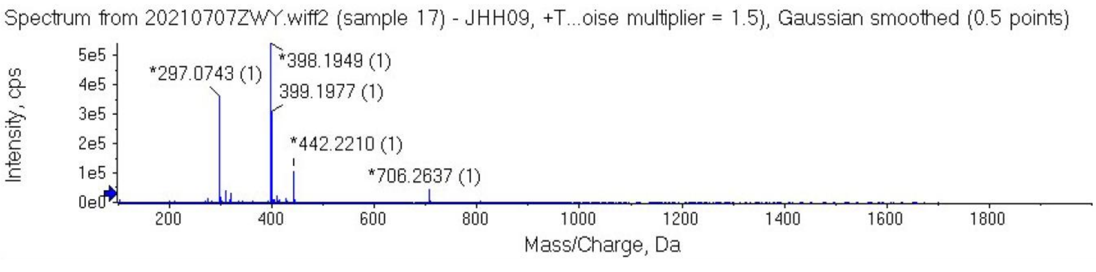

The ^{1}H-NMR, ^{13}C-NMR and HRMS spectra of compound **9b**

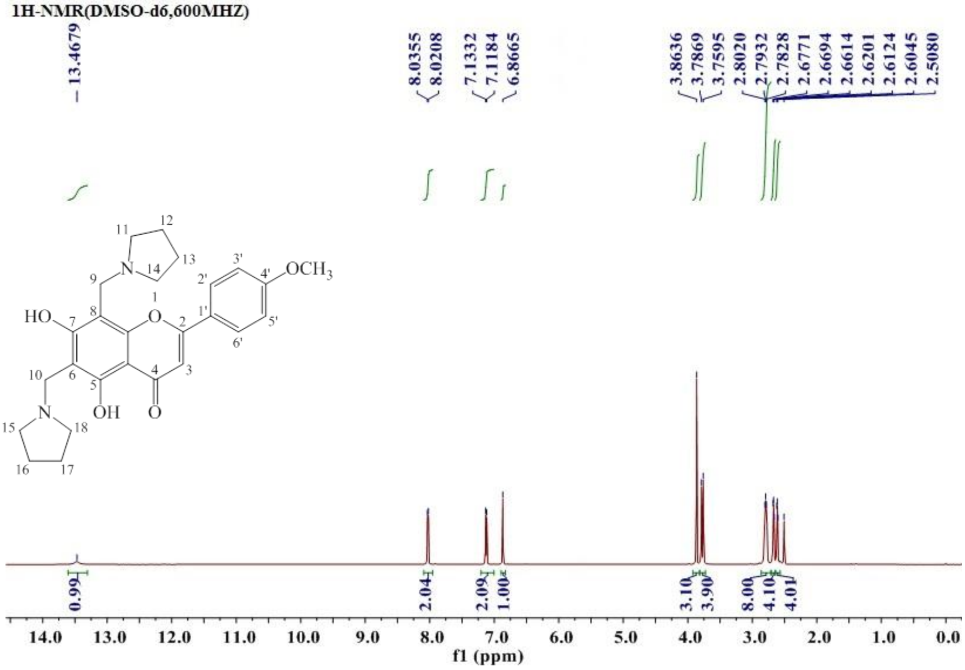

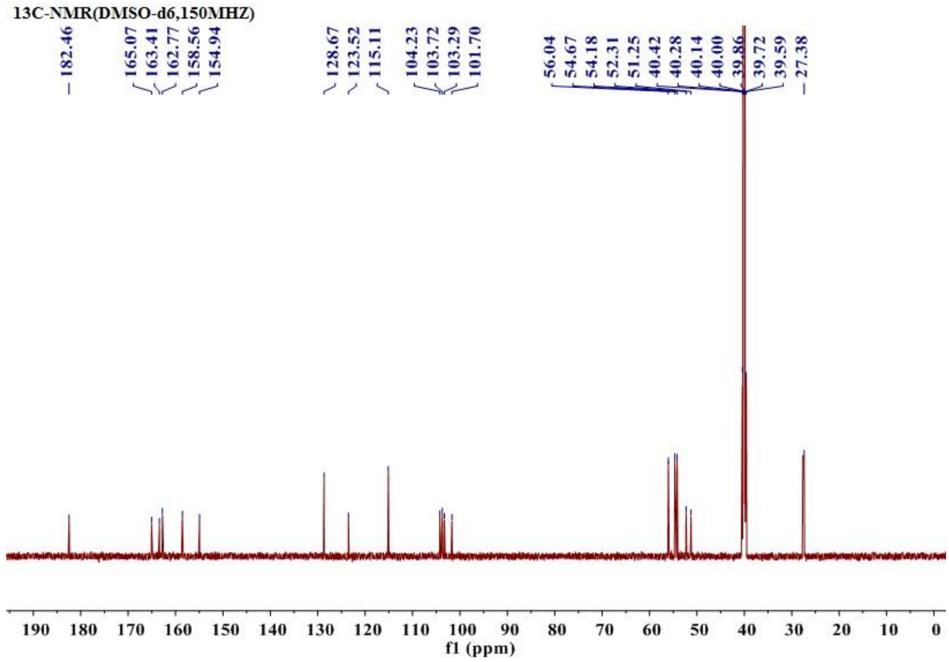

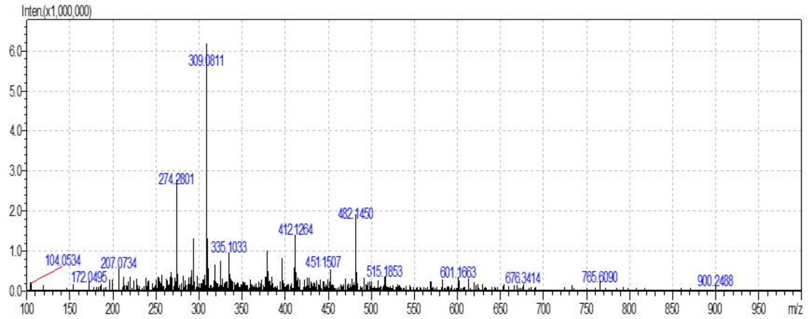

The ^{1}H-NMR, ^{13}C-NMR and HRMS spectra of compound **10a**

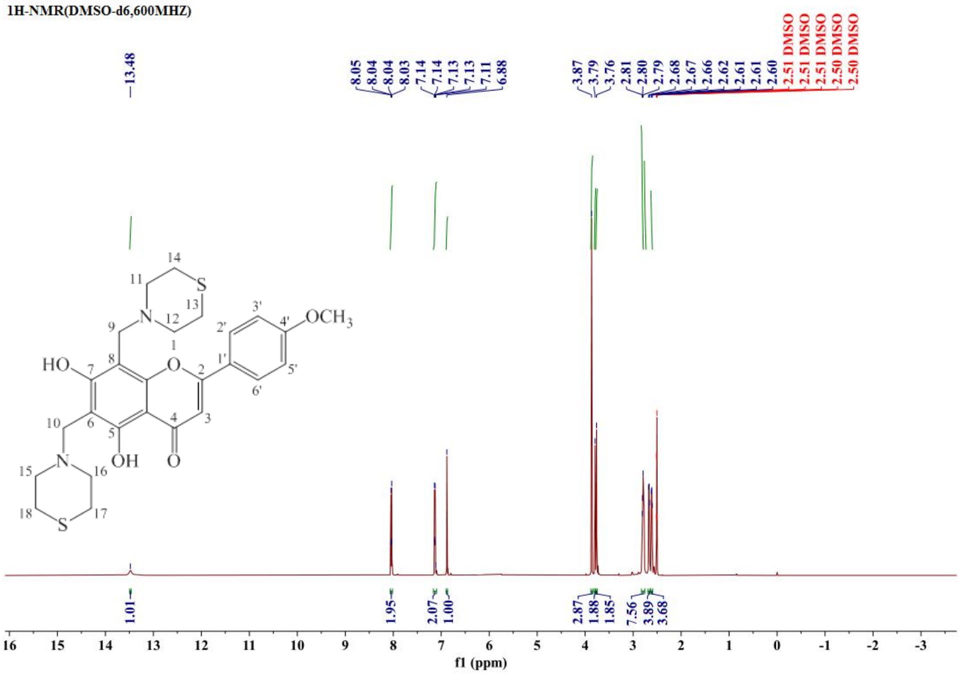

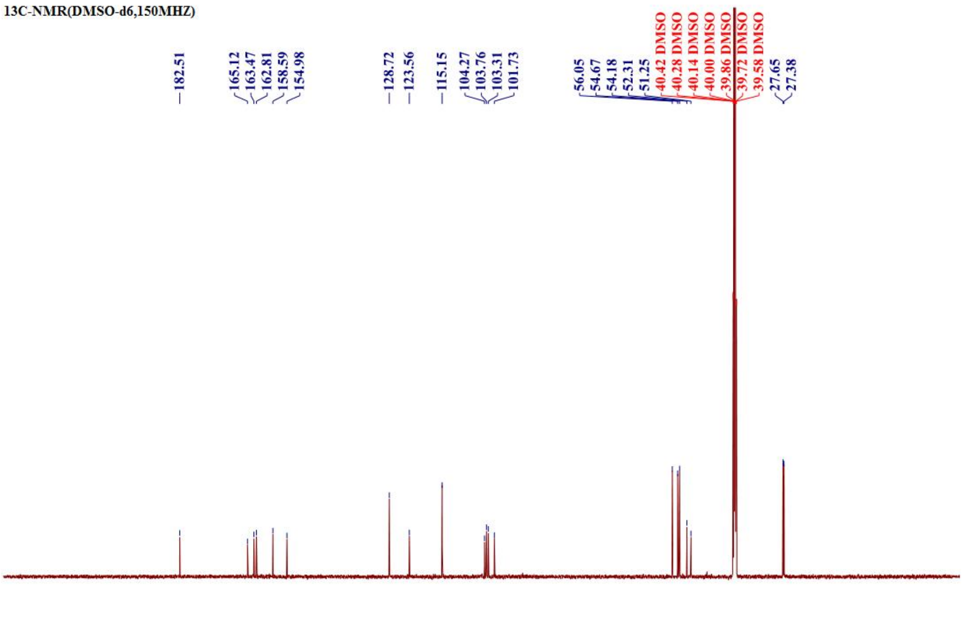

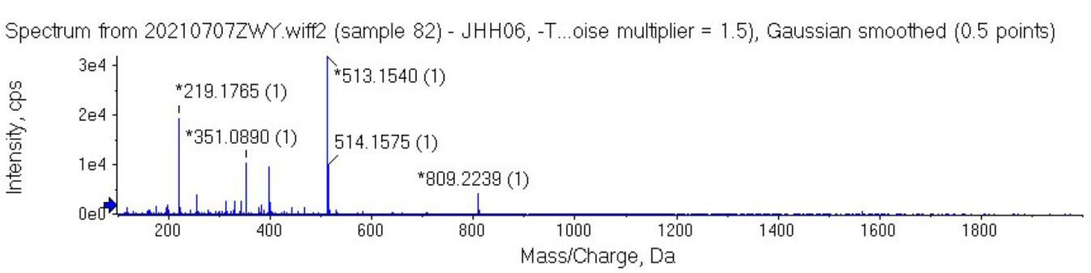

The ^{1}H-NMR, ^{13}C-NMR and HRMS spectra of compound **10b**

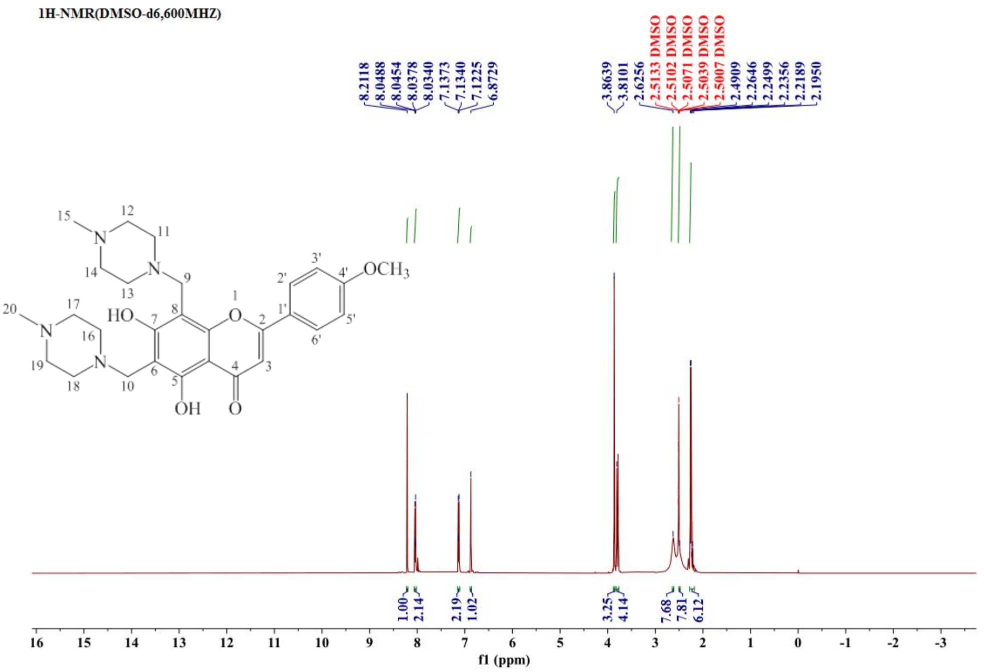

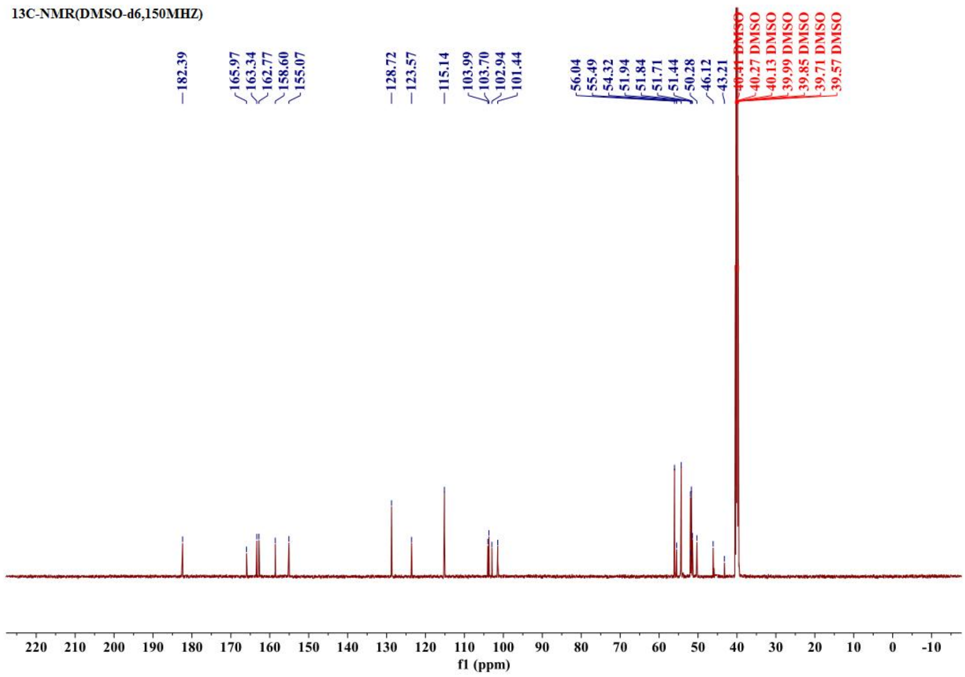

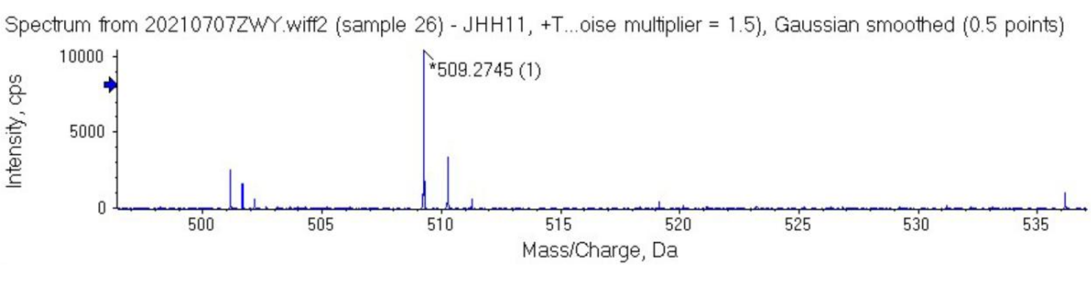

The ^{1}H-NMR, ^{13}C-NMR and HRMS spectra of compound **10c**

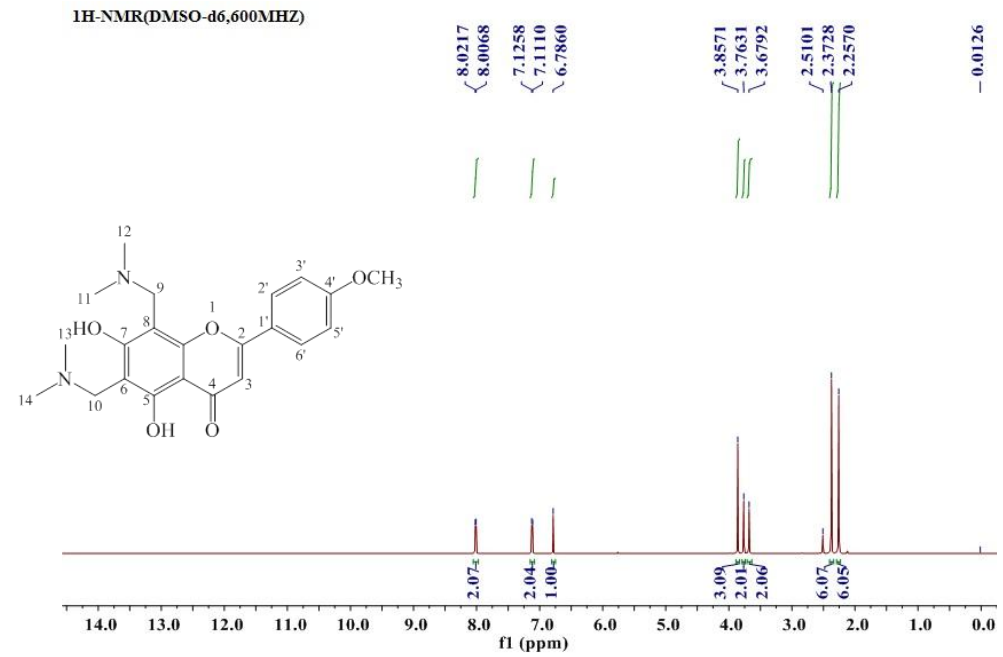

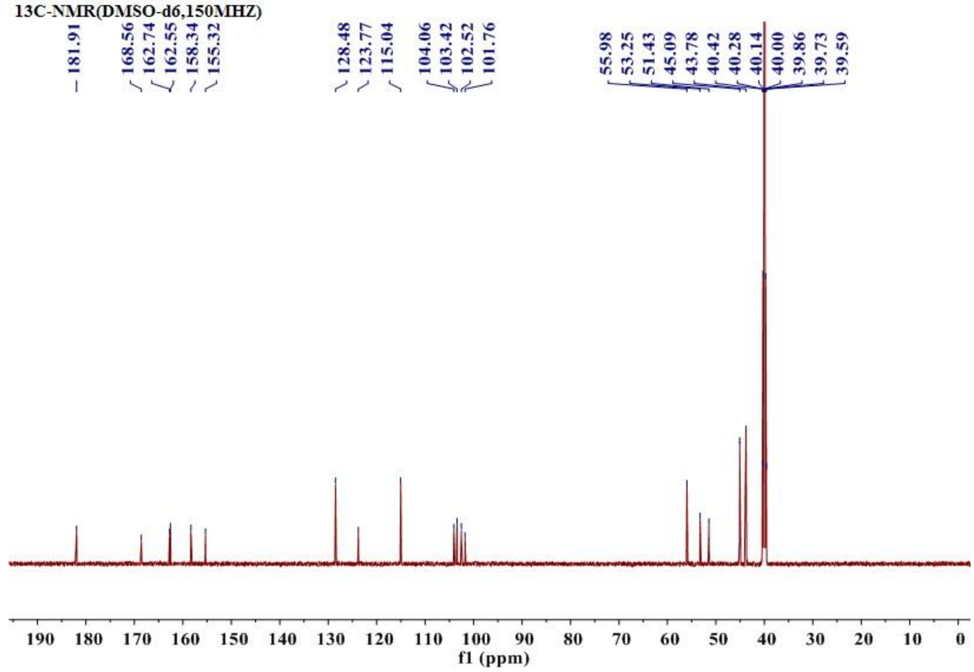

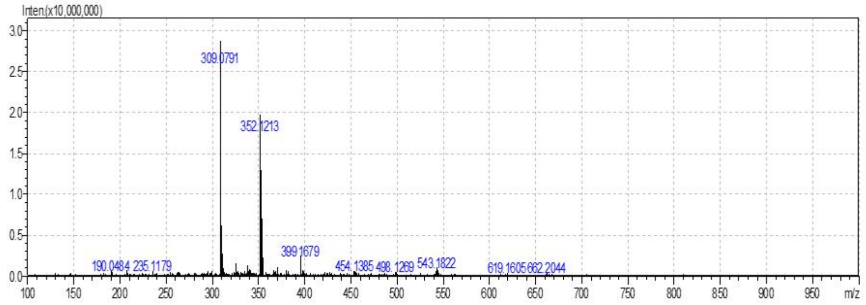

The ^{1}H-NMR, ^{13}C-NMR and HRMS spectra of compound **10d**

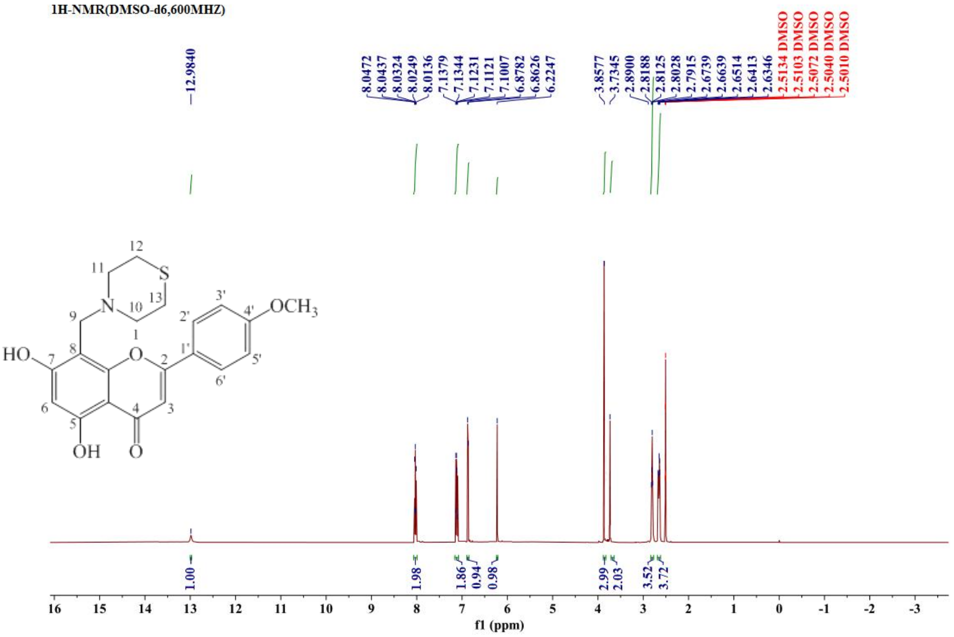

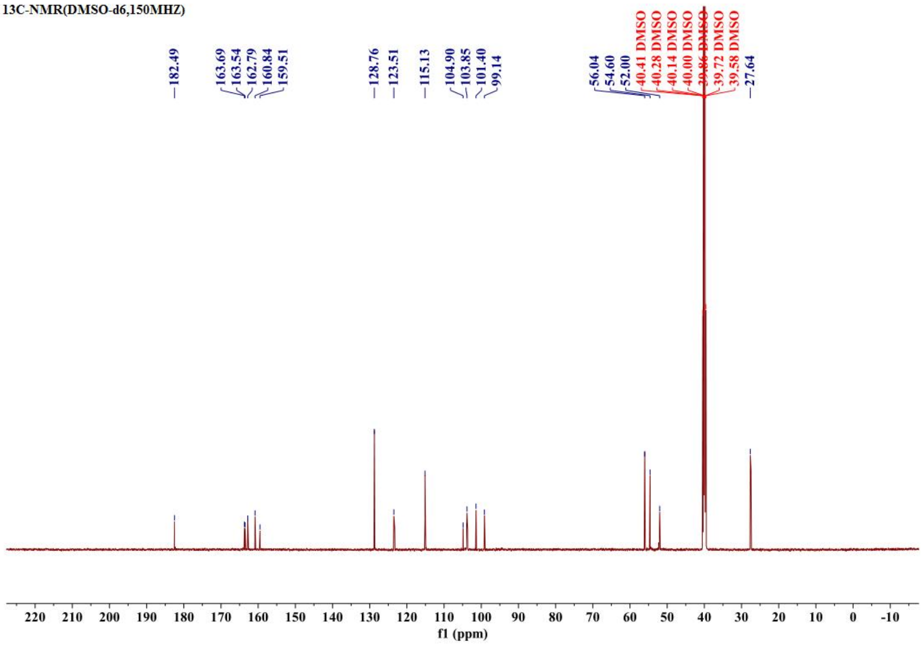

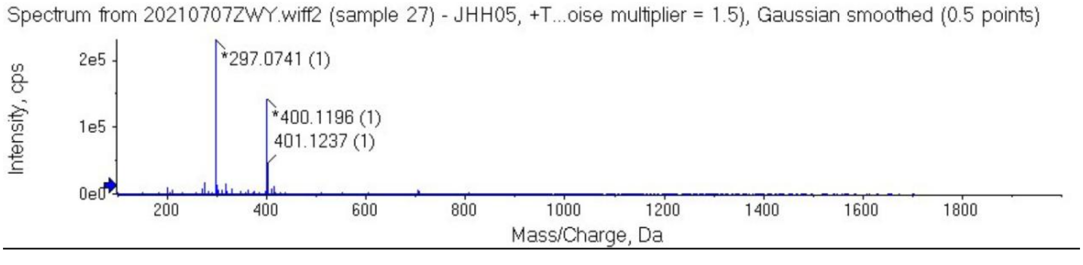

The ^{1}H-NMR, ^{13}C-NMR and HRMS spectra of compound **11a**

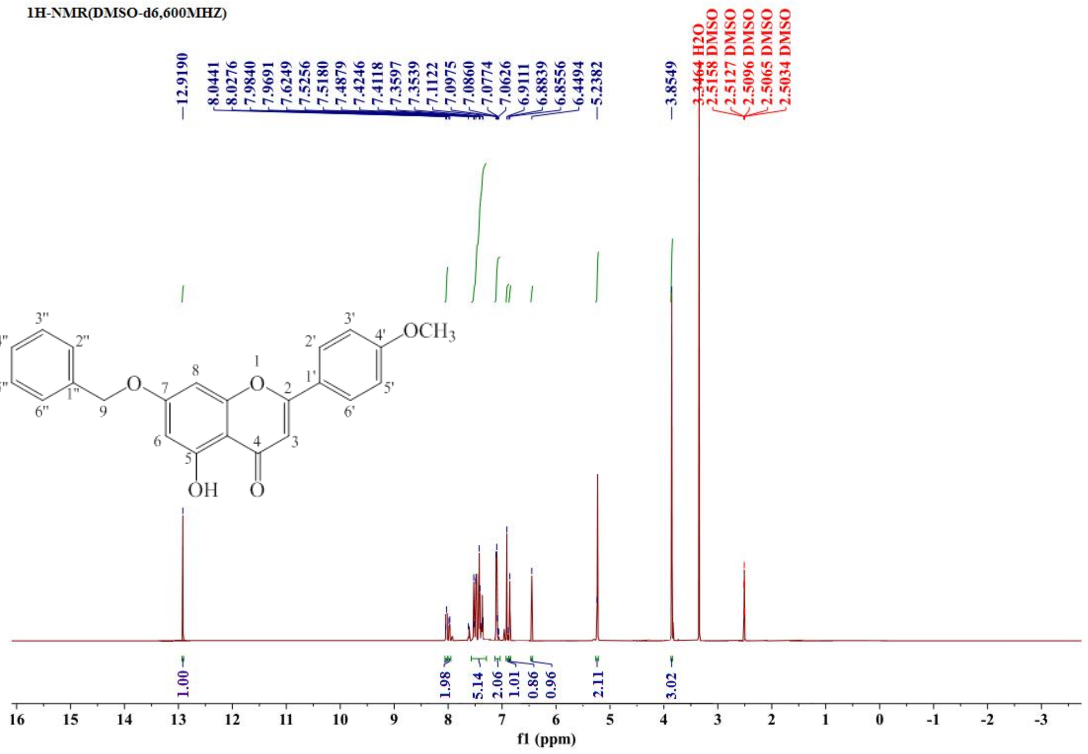

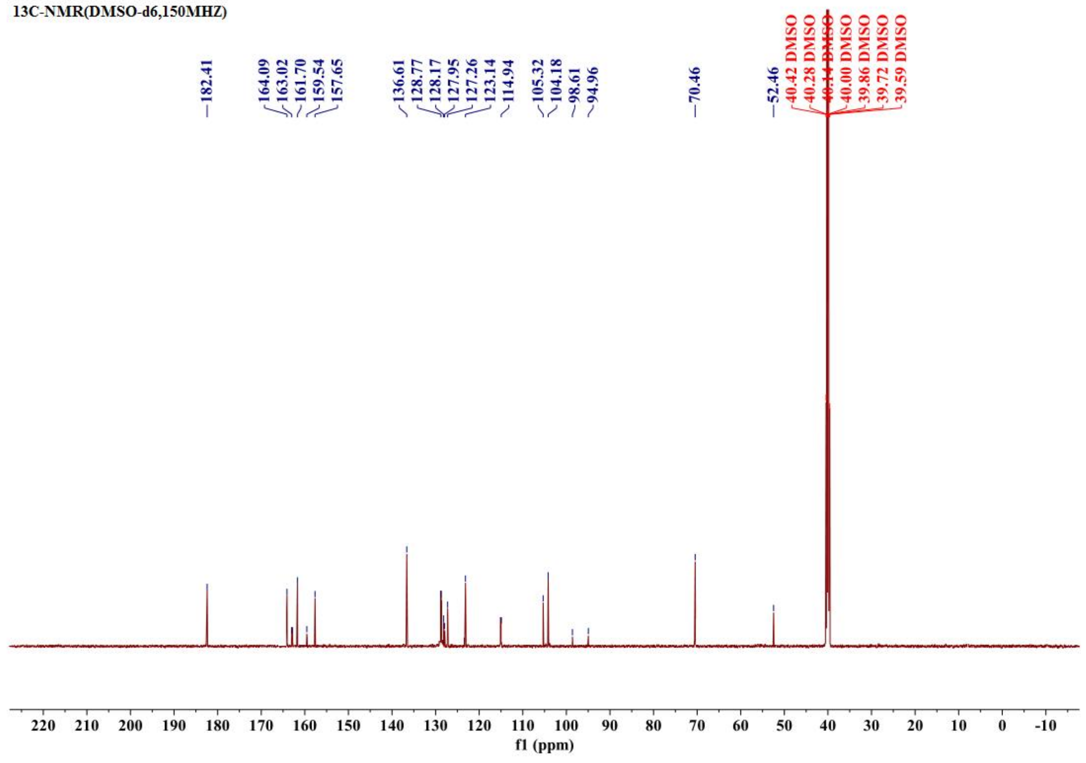

The ^{1}H-NMR and ^{13}C-NMR spectra of compound **12a**

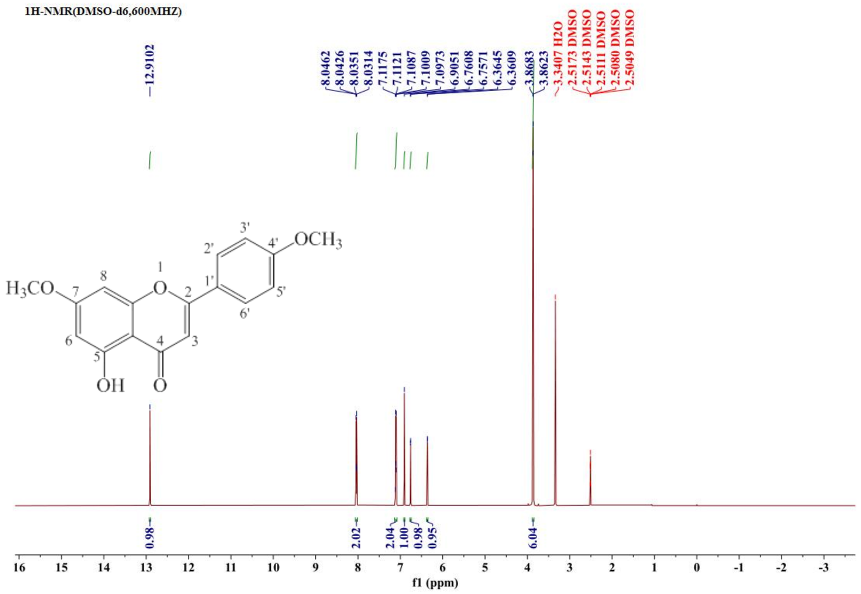

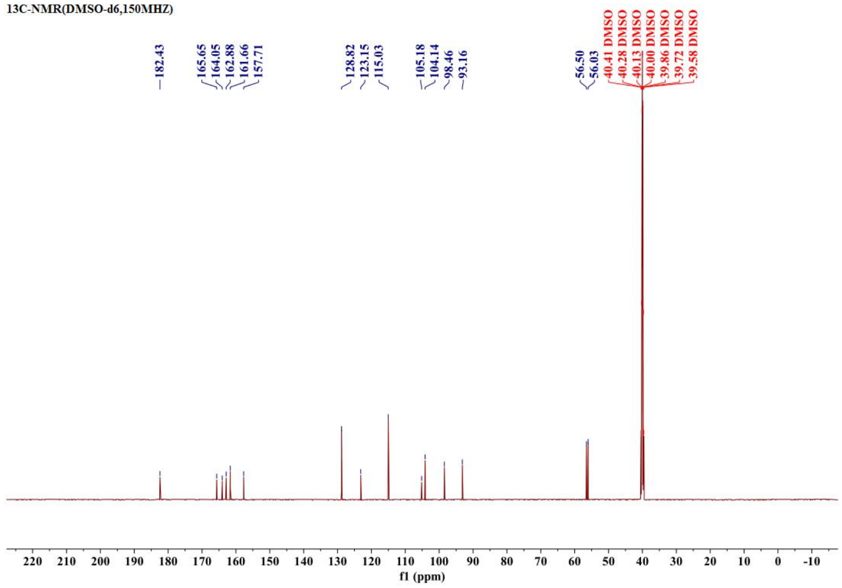

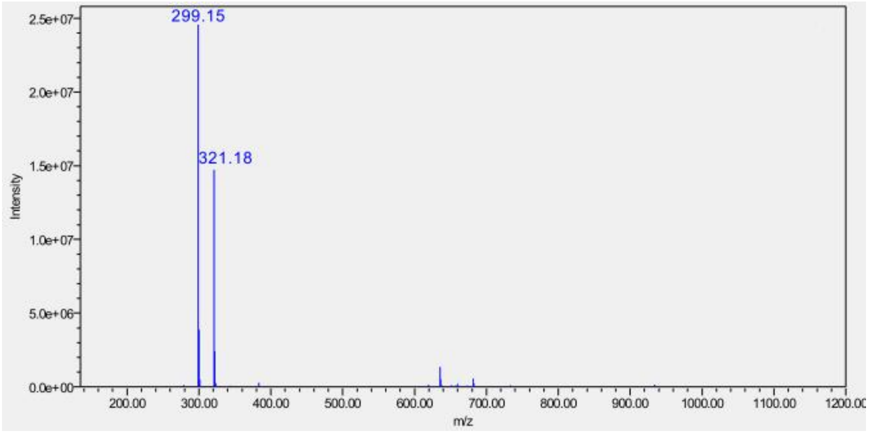

The ^{1}H-NMR, ^{13}C-NMR and MS spectra of compound **12b**

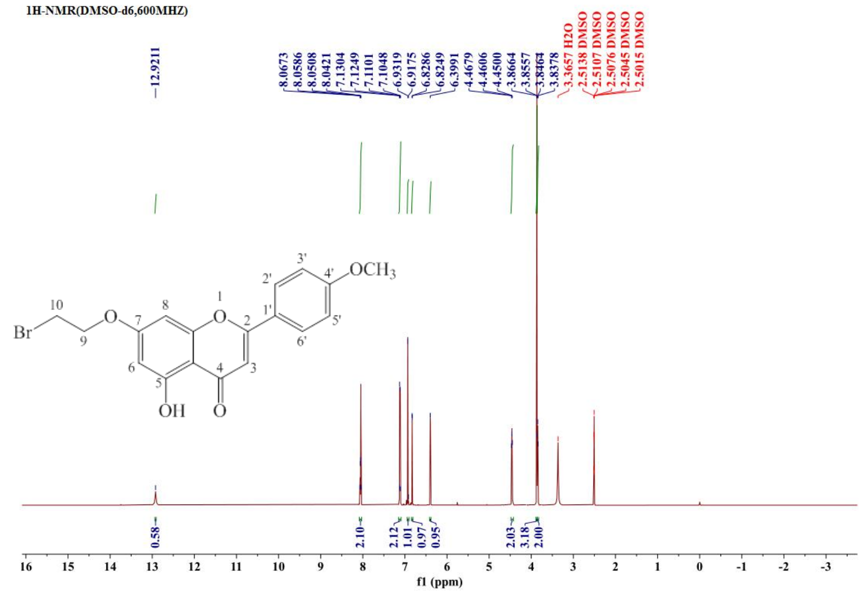

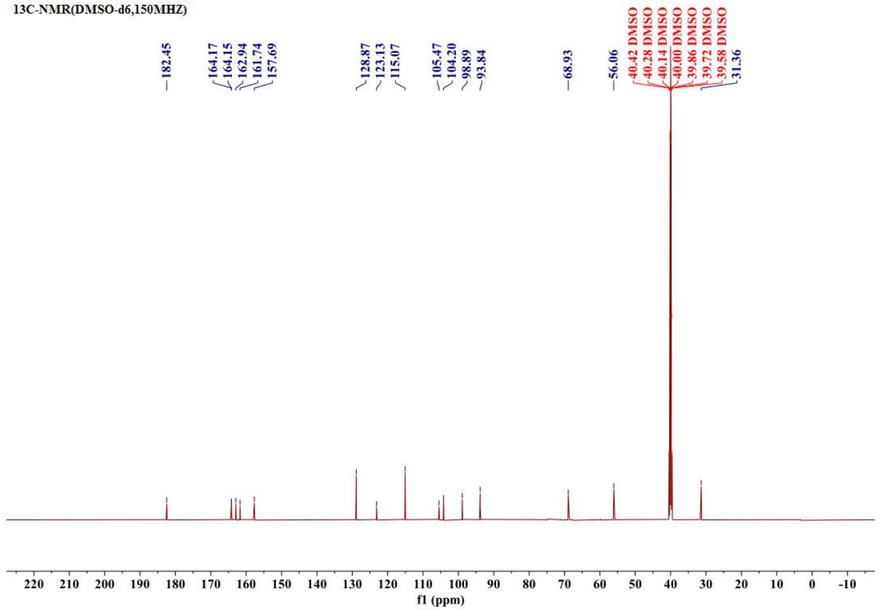

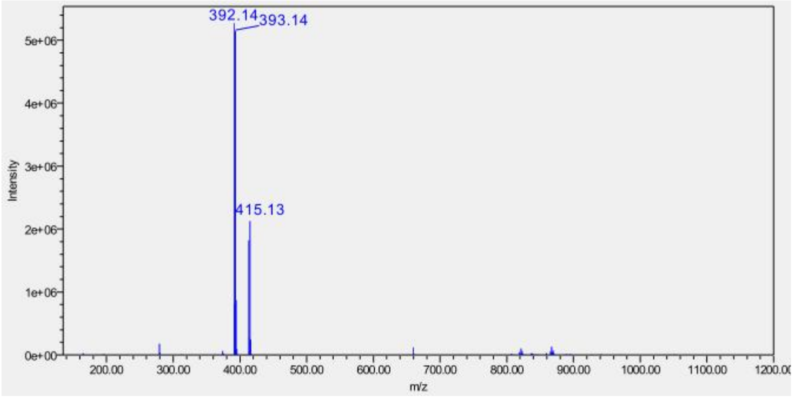

The ^{1}H-NMR, ^{13}C-NMR and MS spectra of compound **12c**

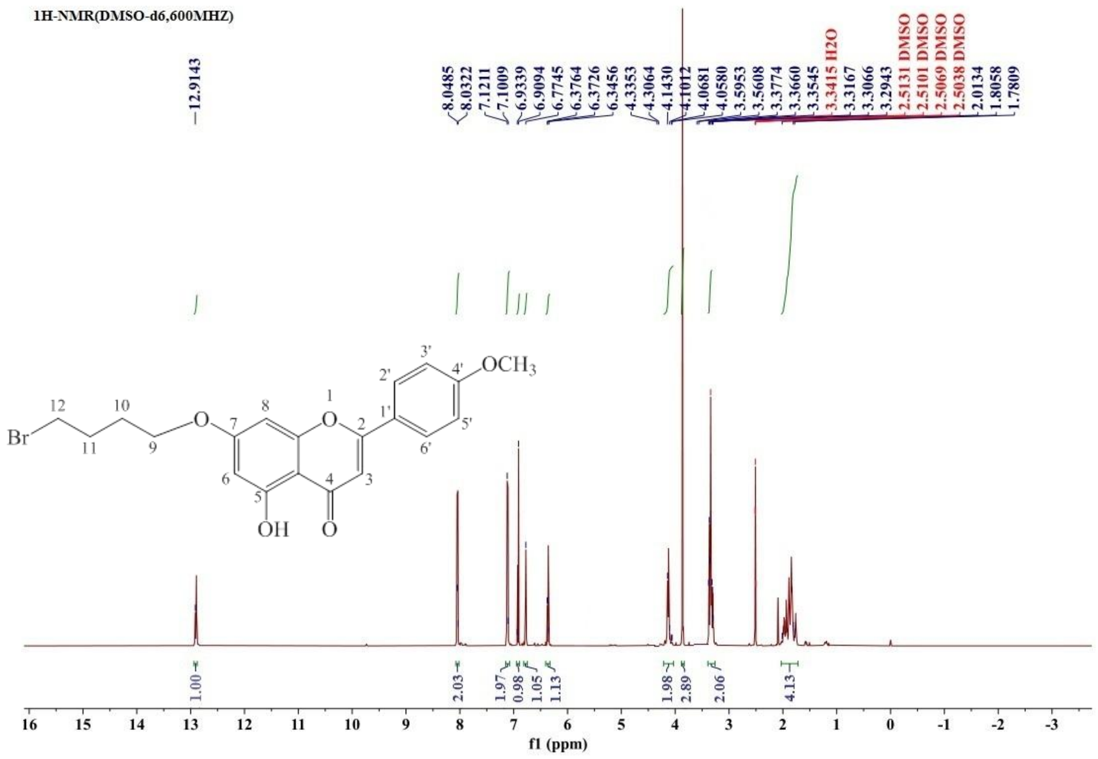

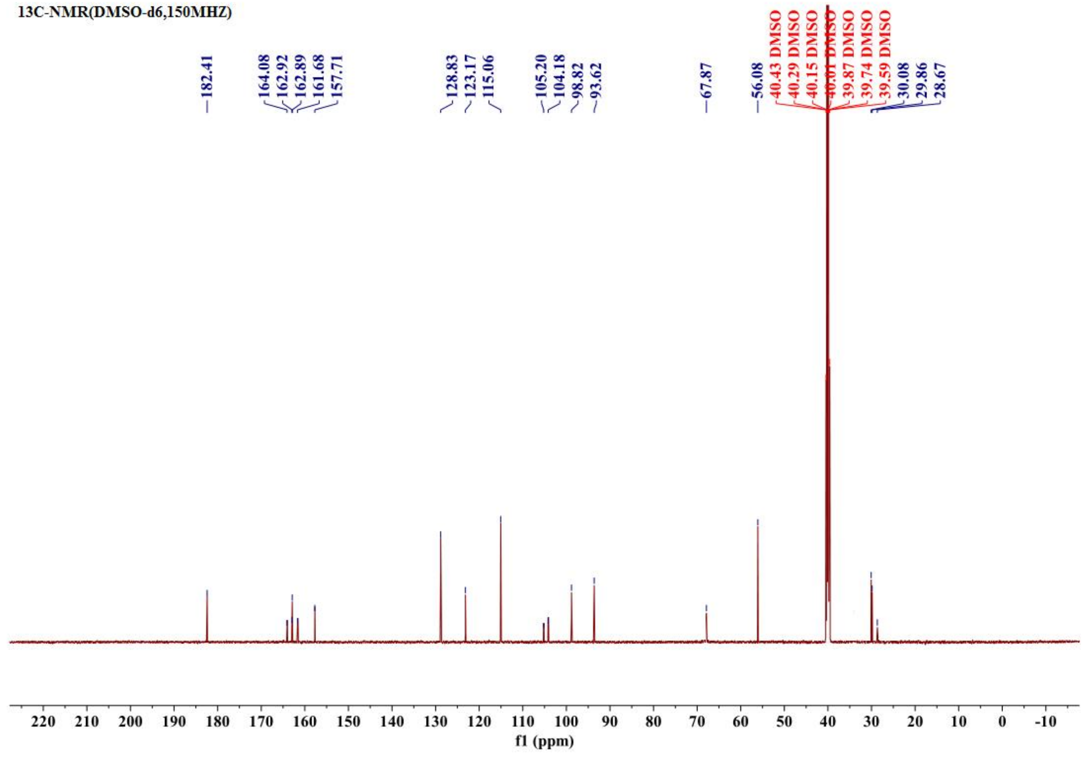

The ^{1}H-NMR and ^{13}C-NMR spectra of compound **12d**
